# Post‐Translational Modifications of TOE3 Regulate Antiviral Defense in Tobacco

**DOI:** 10.1002/advs.202506243

**Published:** 2025-08-14

**Authors:** Bolei Jiao, Baijun Wu, Honghui Lin, Dehui Xi

**Affiliations:** ^1^ Key Laboratory of Bio‐Resource and Eco‐Environment of Ministry of Education College of Life Sciences Sichuan University Chengdu Sichuan 610064 China; ^2^ Southwest Bio‐resources R&D Key Laboratory of Sichuan Province Sichuan University Chengdu Sichuan 610064 China

**Keywords:** phosphorylation, resistance, tobacco mosaic virus, TOE3, ubiquitination

## Abstract

Post‐translational modifications (PTMs) affect the function of transcription factors and regulate plant immune responses. APETALA2 (AP2) transcription factor TARGET OF EAT3 (TOE3) plays a pivotal role in plant antiviral immunity. However, little is known about the impact of PTMs on TOE3 function. Here, that casein kinase II α subunit‐like protein (CK2αL) is identified to interact with TOE3, phosphorylating it at serine 58 (S58) and threonine 128 (T128). Overexpression of *CK2αL* enhances the stability of TOE3 to upregulate the expression of defense‐related genes, thereby improving tobacco resistance to tobacco mosaic virus (TMV). Additionally, the F‐box protein FBXL1 interacts with TOE3 and promotes the ubiquitination‐degradation of TOE3 through the 26S proteasome, and overexpression of *FBXL1* facilitates TMV infection in tobacco. Importantly, CK2αL‐phosphorylated TOE3 exhibits a lower binding affinity to FBXL1 compared to the nonphosphorylated TOE3, thereby protecting TOE3 from FBXL1‐mediated degradation by the 26S proteasome. The stable TOE3 activates the expression of defense‐related genes to enhance the resistance of plants to viral infection. Taken together, the findings demonstrate a mechanism by which phosphorylation and ubiquitination cooperatively regulate the stability of TOE3 to fine‐tune antiviral immunity in tobacco.

## Graphical abstract

CK2αL phosphorylates TOE3 and enhances its stability, while FBXL1 promotes the ubiquitination‐degradation of TOE3 via the 26S proteasome. Compared to CK2αL‐phosphorylated TOE3, nonphosphorylated TOE3 shows a much higher affinity for FBXL1 and is more susceptible to ubiquitination‐degradation, thus facilitating viral infection. The regulatory mechanisms provide insights into the roles of TOE3's PTMs in tobacco antiviral defense.

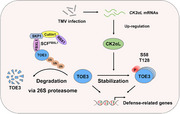

## 1 Introduction

Viral diseases pose a serious threat to plant growth and crop production, leading to severe losses of both crop yield and quality worldwide.^[^ ^1^, ^2^ ^]^ Among the most destructive viruses causing these plant diseases is tobacco mosaic virus (TMV), which is capable of infecting ≈400 species across ≈36 families.^[^ ^3^, ^4^ ^]^ During the process of viral infection, plants deploy many intermediate transcription factors (TFs) as molecular switches that transduce immune signals into the changes in defense gene expression.^[^ ^5^, ^6^ ^]^ The key TFs involved in antiviral responses include APETALA2/ETHYLENE‐RESPONSIVE ELEMENT BINDING FACTOR (AP2/ERF), WRKY, bZIP, bHLH, MYB, and NAC families, which orchestrate immune outputs through dynamic regulatory networks.^[^ ^7^ ^]^ The activities of these TFs are tightly modulated by post‐translational modifications (PTMs), such as phosphorylation, ubiquitination, and SUMOylation, which affect their stability, localization, and DNA‐binding capacity.^[^ ^8^ ^]^ Our recent research found that the AP2 TF TARGET OF EAT3 (TOE3) acts as a critical antiviral regulator by activating the expression of defense‐related genes.^[^ ^9^ ^]^ However, the PTMs of TOE3 in response to viral infection remain largely unknown.

Casein kinase II (CK2) is a conserved serine/threonine protein kinase in eukaryotes.^[^ ^10^ ^]^ The CK2 holoenzyme, showing a typical tetrameric structure in tobacco, is composed of two α catalytic subunits and two β regulatory subunits.^[^ ^11^ ^]^ CK2 plays an essential role in orchestrating plant defense responses and developmental processes. For example, the coat protein (CP) of bamboo mosaic virus (BaMV) is phosphorylated by CK2α in the plasmodesmata, thus affecting viral RNA‐binding affinity and cell‐to‐cell movement.^[^ ^12^ ^]^ During pathogen infection, CK2 can also phosphorylate some important transcription factors to regulate their DNA‐binding activities. A recent study reported that the phosphorylation of MYC2 by CK2 enhances the transcriptional binding activity of MYC2 to the promoters of jasmonic acid (JA)‐response genes, thereby increasing the resistance of *Arabidopsis thaliana* to *Botrytis cinerea*.^[^ ^13^ ^]^ By contrast, phosphorylation of OsTGA5 by CK2 compromises its suppression of defense‐related gene transcription in rice, thus inhibiting the invasion of blast fungus.^[^ ^14^ ^]^ CK2‐mediated phosphorylation has also been shown to alter the protein–protein binding capability or stability of substrates. In light signaling, CK2‐phosphorylated ELONGATED HYPOCOTYL 5 (HY5) shows weakened interaction with the E3 ubiquitin (Ub) ligase CONSTITUTIVE PHOTOMORPHOGENIC 1 (COP1), and is more stable compared to nonphosphorylated HY5.^[^ ^15^ ^]^ CK2‐mediated phosphorylation also affects the stability of two other regulators of photomorphogenesis, LONG HYPOCOTYL IN FAR‐RED LIGHT 1 (HFR1) and PHY‐INTERACTING FACTOR 1 (PIF1).^[^ ^16^, ^17^ ^]^ The phosphorylation of the aforementioned three factors plays a key role in the light‐regulated developmental switch. In *A. thaliana*, CK2 phosphorylates OPEN STOMATA 1 (OST1), thus significantly increasing the binding capability of OST1 to Protein phosphortase 2C (PP2C) and triggering OST1 degradation, negatively regulating the ABA response.^[^ ^18^ ^]^

The ubiquitin proteasome system (UPS) is one of the most efficient regulatory mechanisms utilized by plants to modulate protein abundance, involving an interplay between three key enzymes: E1 Ub‐activating enzymes, E2 Ub‐conjugating proteins, and, finally, E3 Ub ligases, which catalyze Ub from E2 to the target substrate and form a Ub chain.^[^ ^19^, ^20^ ^]^ F‐box proteins, one of which is a component of the multi‐subunit SKP1‐CULLIN1‐F‐box (SCF) E3 ubiquitin ligase complex, play an essential role in regulating plant resistance to biotic stress.^[^ ^21^, ^22^ ^]^ It is well known that *Coronatine‐insensitive 1* (*COI1*) encodes an F‐box protein present in SCF^COI1^, the core module of the SCF^COI1^/JAZ/MYC2 complex that regulates defense responses through the JA signaling pathway.^[^ ^23^, ^24^, ^25^ ^]^ In maize, an F‐box protein ZmFBL41 recruits cinnamyl alcohol dehydrogenase (ZmCAD) for 26S proteasome‐mediated degradation. However, natural variation in ZmFBL41 changes its binding ability toward the target protein ZmCAD, leading to the accumulation of lignin and increased resistance to *Rhizoctonia solani*.^[^ ^26^ ^]^ Previous work revealed that the F‐box protein FBW2 assembles an SCF complex (SCF^FBW2^) that maintains the homeostasis of ARGONAUTE 1 (AGO1) by preferentially degrading its unloaded form, showing the potential of F‐box proteins to influence plant defense against viruses through quality control of the RNA‐silencing process.^[^ ^27^ ^]^

In this study, we found that CK2αL interacts with TOE3 and phosphorylates it at serine 58 (S58) and threonine 128 (T128). CK2αL confers tobacco antiviral resistance by stabilizing TOE3. Furthermore, phosphorylated TOE3 is more stable and has a lower affinity to FBXL1 compared to nonphosphorylated TOE3, thus reducing the degradation of TOE3 by FBXL1‐mediated ubiquitination. This stable, phosphorylated TOE3 inhibits viral infection by improving the expression levels of defense‐related genes. Overall, our results reveal that phosphorylation and ubiquitination of TOE3 fine‐tune antiviral defense in tobacco.

## 2 Results

### 2.1 CK2αL Physically Interacts with TOE3 In Vitro and In Vivo

Our previous study demonstrated that TOE3 positively regulates the resistance of tobacco against TMV infection.^[^ ^9^ ^]^ In order to identify potential regulators of TOE3, we performed yeast two‐hybrid (Y2H) screen assays using the full‐length TOE3 as the bait and a normalized *Nicotiana tabacum* cDNA library as the prey. A potential interaction between TOE3 and a kinase CK2αL was found (Table S1, Supporting Information).

*N. tabacum* is an interspecific hybrid that possesses two subgenomes, the T subgenome derived from *N. tomentosiformis* and the S genome from *N. sylvestris*. *CK2αL* from *N. sylvestris* (*CK2αL‐S*) and *CK2αL* from *N. tomentosiformis* (*CK2αL‐T*) are two homoeologous genes of *CK2αL* in *N. tabacum*. The CDS sequences of *CK2αL‐S* and *CK2αL‐T* show high identity (96.90%) (Figure S1A,B, Supporting Information). However, a recent study demonstrated that the expression level of *CK2αL‐T* is significantly higher than that of *CK2αL‐S* (Figure S1C, Supporting Information).^[^ ^28^ ^]^ Thus, CK2αL‐T was selected for further study and was represented by CK2αL for the smoothness and convenience of description.

**Figure 1:**
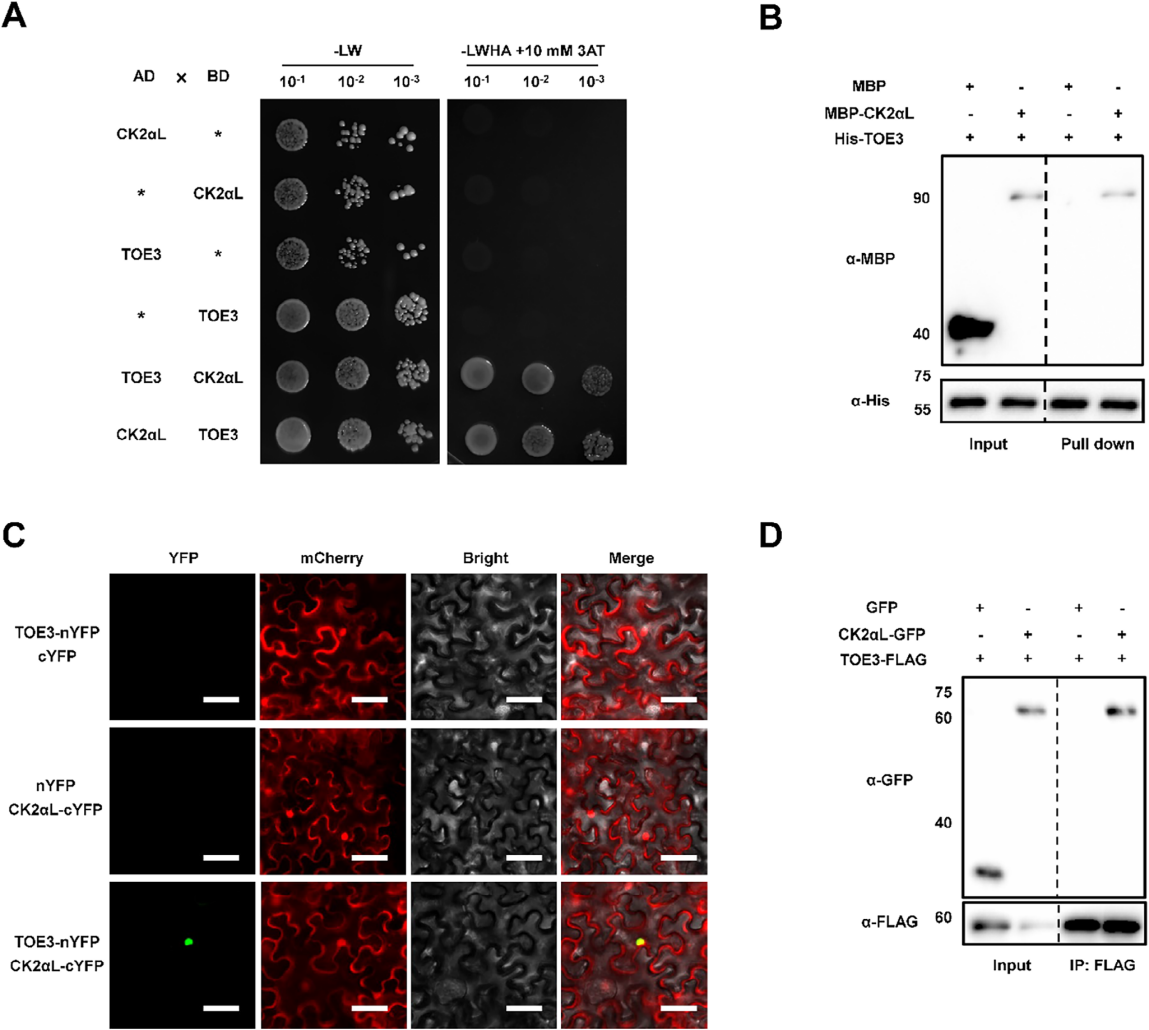
CK2αL interacts with TOE3. A) Y2H assays of the interaction between TOE3 and CK2αL. 10 mm of 3‐AT was used to inhibit self‐activation activity. B) In vitro pull‐down assays of the interaction between TOE3 and CK2αL. WB analysis was performed using anti‐His and anti‐MBP antibodies, respectively. C) BiFC assays of the interaction between TOE3 with CK2αL in the leaves of *N. benthamiana*. A control construct (35S: mCherry) was co‐infiltrated with a couples of BiFC constructs and served as a marker for successful transfection. Scale bars represent 20 µm. D) Co‐IP assays showing the interaction between CK2αL and TOE3 *in planta*. WB analysis was performed using anti‐FLAG and anti‐GFP antibodies, respectively.

CK2αL contains a kinase domain and localizes in the nucleus and plasma membrane (Figure S1D,E, Supporting Information). We then found that TMV inoculation significantly promoted the transcriptional expression of *CK2αL* (Figure S1F, Supporting Information). These findings indicate that CK2αL might work as a kinase and is involved in the tobacco defense response to TMV infection. To test the direct interaction between CK2αL and TOE3, we conducted a Y2H assay, and found that the yeast cells only grew on the Quadruple DO media with 10 mm 3‐AT when AD‐TOE3 and BD‐CK2αL or AD‐CK2αL and BD‐TOE3 were co‐transformed into yeast cells. This result suggested that CK2αL interacted with TOE3 in yeast cells (**Figure** **1**A). Next, we performed an in vitro His pull‐down assay to validate the interaction between CK2αL and TOE3. We first purified maltose‐binding protein (MBP), MBP‐CK2αL, and His‐TOE3 proteins in vitro and incubated MBP and MBP‐CK2αL proteins with His‐TOE3 for 4 h. Then, we used Ni‐NTA agarose beads to bind His‐TOE3 and its interaction proteins. The beads were collected and boiled to perform Western blotting (WB) analysis using anti‐MBP and anti‐His antibodies. The WB results revealed that His‐TOE3 can only pull down MBP‐CK2αL protein, but not MBP protein (Figure 1B). To further confirm the interaction between CK2αL and TOE3 in vivo, we then performed a bimolecular fluorescence complementation (BiFC) assay. As shown in Figure 1C, when we co‐expressed TOE3 fused with the N‐terminus of yellow fluorescent protein (TOE3‐nYFP) and CK2αL fused to the C‐terminus of YFP (CK2αL‐cYFP) in *Nicotiana benthamiana* leaves, the YFP fluorescence signals were detected in the nucleus, which indicated a physical interaction between TOE3 and CK2αL *in planta* (Figure 1C). Co‐immunoprecipitation (Co‐IP) assays were further carried out by overexpressing TOE3‐FLAG along with CK2αL‐GFP or GFP (as a negative control) into *N. benthamiana* leaves. The results showed that TOE3‐FLAG co‐precipitated with CK2αL‐GFP, but not GFP, *in planta* (Figure 1D). Taken together, these results demonstrate that CK2αL interacts with TOE3 both in vitro and in vivo.

As the CK2 enzyme is composed of two α subunits and two β subunits in *N. tabacum*,^[^ ^29^ ^]^ we then tested whether TOE3 interacts with other subunits of CK2. The results of our Y2H and BiFC assays showed that TOE3 did not interact with other subunits of CK2 (Figure S2, Supporting Information). In addition, we also confirmed that there was no interaction between CK2αL and the four subunits of CK2 (Figure S3, Supporting Information). These results indicate that CK2αL might work as a kinase, and independently phosphorylate TOE3.

### 2.2 CK2αL Phosphorylates TOE3 at S58 and T128

**Figure 2:**
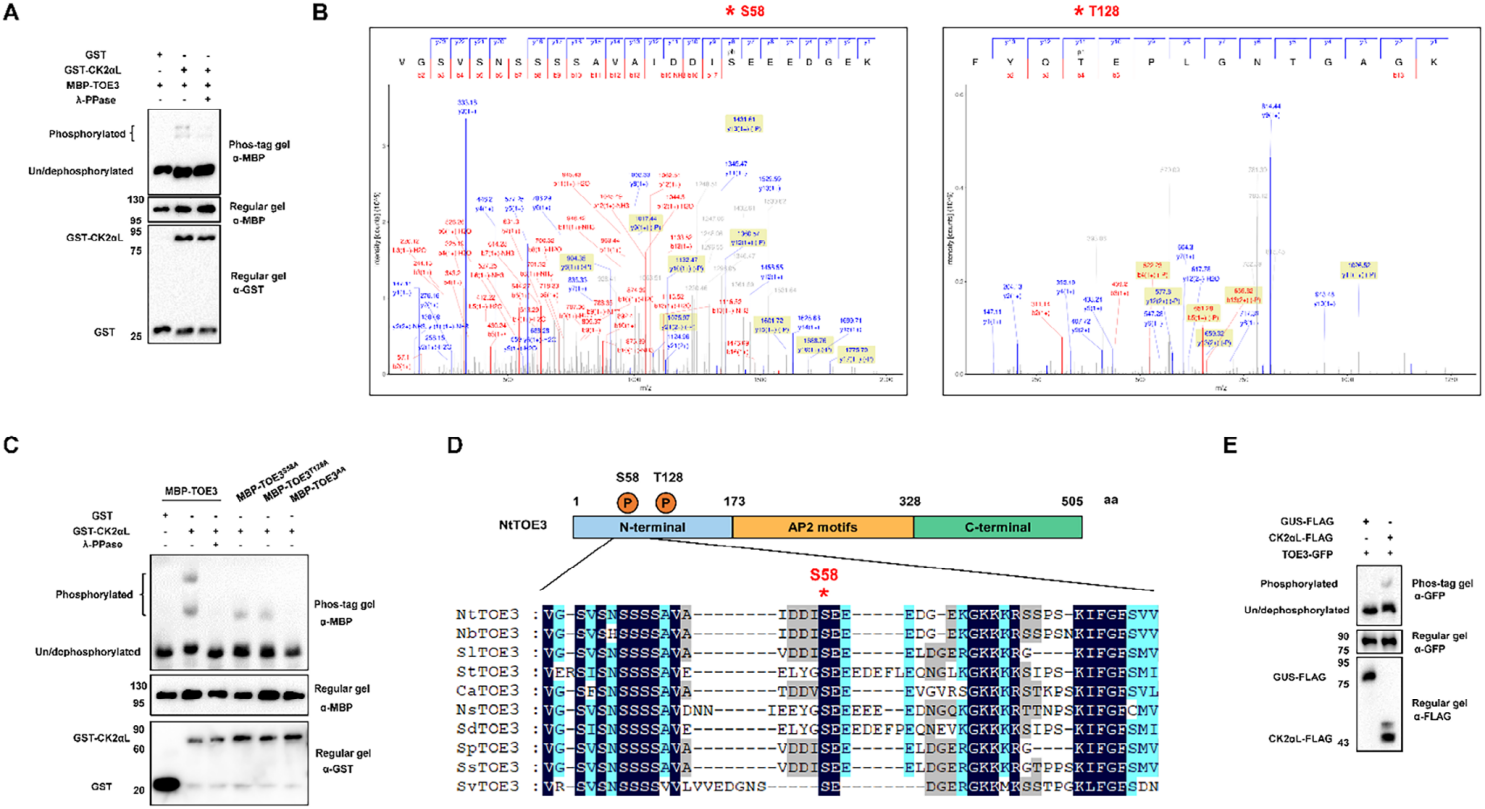
CK2αL phosphorylates TOE3 at S58 and T128. A) Detection of TOE3 phosphorylation by CK2αL kinase in vitro using Phos‐tag assays. The kinase assay was performed with the incubation of GST‐CK2αL and MBP‐TOE3 at 30 °C for 1 h. Protein was separated in the SDS–PAGE gel containing Phos‐tag reagent. WB analysis was performed using anti‐GST and anti‐MBP antibodies, respectively. Phosphorylated TOE3 showed slower gel mobility. The phosphorylation of TOE3 by CK2αL was inhibited by λ‐PPase. B) Identification of the phosphorylated TOE3 residues by CK2αL via mass spectrometry analysis. Annotated spectra for the phosphorylated peptide of TOE3 are shown at the top. Red asterisks represent phosphorylation sites. C) In vitro kinase assays detecting the phosphorylation of TOE3, TOE3^S58A^, TOE3^S128A^, and TOE3^AA^ by CK2αL. The purified MBP‐TOE3, MBP‐TOE3^S58A^, MBP‐TOE3^S128A^, or MBP‐TOE3^AA^ were incubated with GST‐CK2αL at 30 °C for 1 h. Proteins were separated in SDS–PAGE gel containing Phos‐tag reagent, followed by immunoblotting using anti‐MBP or anti‐GST antibodies, respectively. D) Sequence alignment of TOE3 from *Solanaceae* species rendered with ESPript 3. Identical and similar residues are colored dark blue and light blue, respectively. The red asterisk highlights the conserved phosphorylation site S58 of TOE3. E) In vivo kinase assays of TOE3 by CK2αL kinase *in planta*. TOE3‐GFP, along with GUS‐FLAG or CK2αL‐FLAG constructs, was co‐infiltrated into *N. benthamiana* leaves. Then, total proteins were extracted from *N. benthamiana* leaves and subjected to IP with anti‐GFP beads. Proteins were separated in the SDS–PAGE gel containing Phos‐tag reagent, followed by immunoblotting using anti‐GFP or anti‐FLAG antibodies, respectively.

To confirm the phosphorylation of TOE3 by CK2αL, we performed the in vitro kinase assays using Phos‐tag gel, in which phosphorylated proteins bind to the Phos‐tag reagent, reducing their mobility. The results showed that the migration of phosphorylated MBP‐TOE3 was clearly retarded in the presence of GST‐CK2αL. Additionally, the abundance of phosphorylated bands was weakened in the addition of lambda phosphatase (λ‐PPase), confirming that the upper bands were indeed CK2αL‐phosphorylated TOE3 (**Figure** **2**A). We also employed an anti‐phospho‐Ser/Thr (anti‐pS/T) antibody‐based detection assay to validate the phosphorylation of TOE3 by CK2αL (Figure S4, Supporting Information). The results demonstrate that CK2αL phosphorylates TOE3 in vitro.

Next, to identify the phosphorylation site(s), we analyzed the peptides of phosphorylated TOE3 obtained from the in vitro kinase assay via mass spectrometry. The results identified serine (S) 58 and threonine (T) 128 as two potential CK2αL‐mediated phosphorylation sites (Figure 2B). To further confirm the phosphorylation sites, S58 or T128 of TOE3 were mutated to alanine (TOE3^S58A^, TOE3^T128A^, and TOE3^AA^). The results showed that, in the presence of GST‐CK2αL, the phosphorylation bands of MBP‐TOE3^S58A^ and MBP‐TOE3^T128A^ were reduced compared to MBP‐TOE3. Importantly, when the two sites were both mutated to alanine, the CK2αL‐phosphorylated bands of MBP‐TOE3^AA^ were undetectable. These results demonstrate that CK2αL phosphorylates TOE3 at S58 and T128 (Figure 2C). Moreover, the S58 residue of TOE3 is highly conserved among species of the *Solanaceae* family, such as potato, tomato, pepper, and other crops. (Figure 2D).

To further test whether CK2αL phosphorylates TOE3 in vivo, TOE3‐GFP was overexpressed along with CK2αL‐FLAG or GUS‐FALG in *N. benthamiana* leaves. At 3 days post‐agroinfiltration (dpa), we immunoprecipitated TOE3‐GFP protein from infiltrated leaves. WB analysis was performed using Phos‐tag gel to distinguish phosphorylated and dephosphorylated TOE3. We found that the phosphorylated TOE3 could only be detected when CK2αL‐FLAG was overexpressed (Figure 2E), indicating that CK2αL can phosphorylate TOE3 in vivo. Thus, TOE3 is phosphorylated by CK2αL both in vitro and in vivo.

### 2.3 Phosphorylation of TOE3 by CK2αL Enhances its Stability

To investigate TOE3 phosphorylation status during TMV infection, we then performed the time course analysis of TOE3 phosphorylation status in TOE3‐OE#4 after TMV infection. The results demonstrated that the phosphorylation level of TOE3 in TMV‐infected leaves was significantly increased compared to that in the mock leaves. Moreover, upon TMV infection, the phosphorylation level of TOE3 at 12 days post‐inoculation (dpi) was significantly higher than that at 4 dpi (Figure S5A,B, Supporting Information). The results show that TMV infection induces the phosphorylation of TOE3.

To further study how CK2αL modulates the function of TOE3, S58 and T128 of TOE3 were mutated to aspartic acid to mimic the phosphorylation of TOE3 by CK2αL (TOE3^DD^), or were mutated to alanine to mimic the nonphophorylated TOE3 (TOE3^AA^). Then, TOE3‐GFP, TOE3^DD^‐GFP or TOE3^AA^‐GFP were co‐infiltrated into *N. benthamiana* leaves with H2B‐mCherry constructs. Observation of their subcellular localization showed that phosphorylated TOE3 and nonphosphorylated TOE3 both localized in the nucleus (Figure S6A, Supporting Information). In addition, our previous study confirmed that TOE3 can directly bind to the promoter of *KL1* and activate the transcriptional expression of *KL1*.^[^ ^9^ ^]^ To detect the transcription activation ability of TOE3^DD^, and TOE3^AA^, we conducted dual‐luciferase assays by co‐expressing 35S: TOE3^DD^, or 35S: TOE3^AA^ with NtKL1_pro_: LUC in *N. benthamiana* leaves and measuring the luciferase (LUC) luminescence intensity. The results indicated no significant difference between them (Figure S6B, Supporting Information), demonstrating that the subcellular localization and transcriptional regulation ability of TOE3 are not affected by CK2αL phosphorylation.

**Figure 3:**
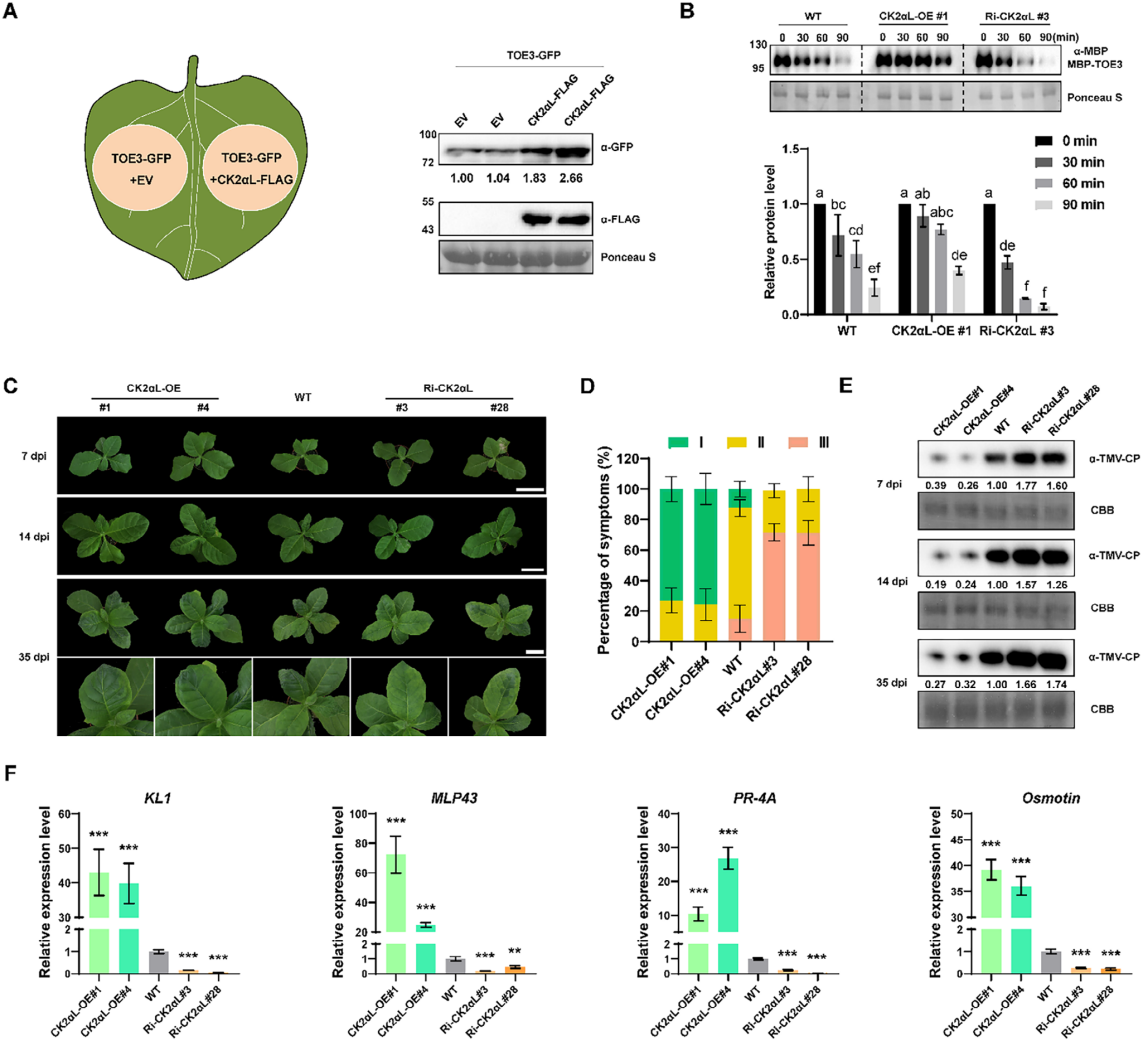
CK2αL stabilizes TOE3 and enhances tobacco antiviral immunity. A) Overexpression of *CK2αL* improved the accumulation of TOE3. TOE‐GFP accumulation in *N. benthamiana* leaves overexpressing EV or CK2αL‐FLAG at 3 dpa was detected by WB analysis using GFP‐ and FLAG‐specific antibodies, respectively. EV, 35S: FLAG empty vector. B) Cell‐free protein degradation assays showing the changes of recombinant MBP‐TOE3 incubated in the extracts from CK2αL‐OE#1, WT, or Ri‐CK2αL#3 leaves, respectively. WB analysis was performed using an anti‐MBP antibody. The initial protein levels before treatment were normalized to 1. Ponceau S staining was performed as a loading control. Data are presented as means ± SD from three independent experiments. Statistical analysis was performed using one‐way ANOVA with Tukey's multiple‐comparison test (different letters represent significantly different groups; *P* <  0.05). C) Disease symptoms of CK2αL‐OE, WT, and Ri‐CK2αL tobacco plants at 7, 14, and 35 dpi. Scale bars = 6 cm. D) The symptom percentages of TMV‐infected CK2αL‐OE, WT, and Ri‐CK2αL plants with different disease symptom grades at 35 dpi. Data are presented as means ± SD from three independent experiments. E) WB analysis of TMV‐CP accumulation in CK2αL‐OE, WT, and Ri‐CK2αL plants at 7, 14, and 35 dpi using anti‐TMV‐CP antibody. F) Expression levels of defense‐related genes activated by TOE3, including *kirola‐like 1* (*KL1*), *major latex protein‐like protein 43* (*MLP43*), *pathogenesis‐related protein 4A* (*PR‐4A*), and *Osmotin*, in CK2αL‐OE, WT, and Ri‐CK2αL plants at 7 dpi. Data are presented as means ± SD from three independent experiments. Significant differences are determined using Student's t‐test (^**^ *P* < 0.01, ^***^ *P* < 0.001).

To determine whether phosphorylation by CK2αL affects the stability of TOE3, we co‐expressed TOE3‐GFP with empty vector (EV) or CK2αL‐FLAG constructs in *N. benthamiana* leaves. The results of WB analysis demonstrated that overexpression of CK2αL‐FLAG increased the accumulation of TOE3‐GFP compared to the EV control (**Figure** **3**A). Then, we generated *CK2αL*‐overexpressing (CK2αL‐OE) transgenic *N. tabacum* plants (Figure S7A,B, Supporting Information). For CK2αL‐S also interacted with TOE3 and the CDS sequences of *CK2αL‐S* and *CK2αL‐T* show high identity (96.70%) (Figures S7C and S1A, Supporting Information), RNA interference (RNAi)‐mediated *CK2αL* knockdown (Ri‐CK2αL) transgenic *N. tabacum* plants were generated, and two homoeologous genes of *CK2αL* could be simultaneously silenced (Figure S7D,E, Supporting Information). The subsequent cell‐free protein degradation assays were performed using recombinant MBP‐TOE3 incubated with protein extracts prepared from wild‐type (WT), CK2αL‐OE#1, and Ri‐CK2αL#3 *N. tabacum* plants. The results showed that the degradation rate of MBP‐TOE3 in the CK2αL‐OE#1 extract was significantly slower than that in the WT extract. In contrast, the degradation rate of MBP‐TOE3 was the highest in the Ri‐CK2αL#3 extract (Figure 3B). Taken together, these data suggest that CK2αL‐mediated phosphorylation enhances the stability of TOE3.

### 2.4 Overexpression of *CK2αL* Improves Tobacco Antiviral Immunity

To test the effect of CK2αL on tobacco immunity against TMV, 28‐day‐old seedlings of WT, CK2αL‐OE, and Ri‐CK2αL lines were inoculated with TMV. The disease symptoms were subsequently categorized based on the severity of systematically infected leaves (Figure S8, Supporting Information). Ri‐CK2αL#3 and Ri‐CK2αL#28 plants appeared with less green recovery tissue and exhibited obvious interveinal chlorotic symptoms caused by TMV infection compared to WT at 14 and 35 dpi. Additionally, milder symptoms and more green recovery tissue were observed in the systemic leaves of CK2αL‐OE lines compared to those in WT plants at 35 dpi. (Figure 3C,D). The WB results showed much higher levels of viral accumulation in Ri‐CK2αL#3 and Ri‐CK2αL#28 plants than in WT plants at 7, 14, and 35 dpi; moreover, the virus titers were lower in CK2αL‐OE lines (Figure 3E). Consistent with the results of the *TOE3*‐overexpressing plants (TOE3‐OE) and *TOE3* knockout plants (CR‐TOE3) in our recently published work,^[^ ^9^ ^]^ RT‐qPCR analysis showed that overexpression of *CK2αL* significantly up‐regulated the expression of defense‐related genes (Figure 3F). These findings indicate that overexpression of *CK2αL* enhances the resistance of *N. tabacum* to TMV.

To further investigate the genetic interplay between CK2αL and TOE3 in antiviral defense, we crossed CK2αL‐OE#1 with the CRISPR‐mediated TOE3 knockout transgenic *N. tabacum* line (CR‐TOE3#68). Then, 28‐day‐old seedlings of WT, CK2αL‐OE#1, CR‐TOE3#68, and the resultant CK2αL‐OE CR‐TOE3 were inoculated with TMV. Severe chlorotic symptoms were observed in CR‐TOE3#68 compared to those in WT at 35 dpi, while milder symptoms and more green recovery tissue were observed in CK2αL‐OE#1. However, overexpression of *CK2αL* did not counteract the severe etiolation caused by TOE3 knockout (Figure S9A,B, Supporting Information). WB analysis of TMV‐CP accumulation confirmed the above conclusion (Figure S9C, Supporting Information), strongly indicating that TOE3 acts downstream of CK2αL in tobacco resistance to TMV infection. Taken together, these findings demonstrate that overexpression of *CK2αL* enhances tobacco defense against TMV by improving the stability of TOE3.

### 2.5 FBXL1 Directly Interacts with and Ubiquitinates TOE3 In Vivo and In Vitro

**Figure 4:**
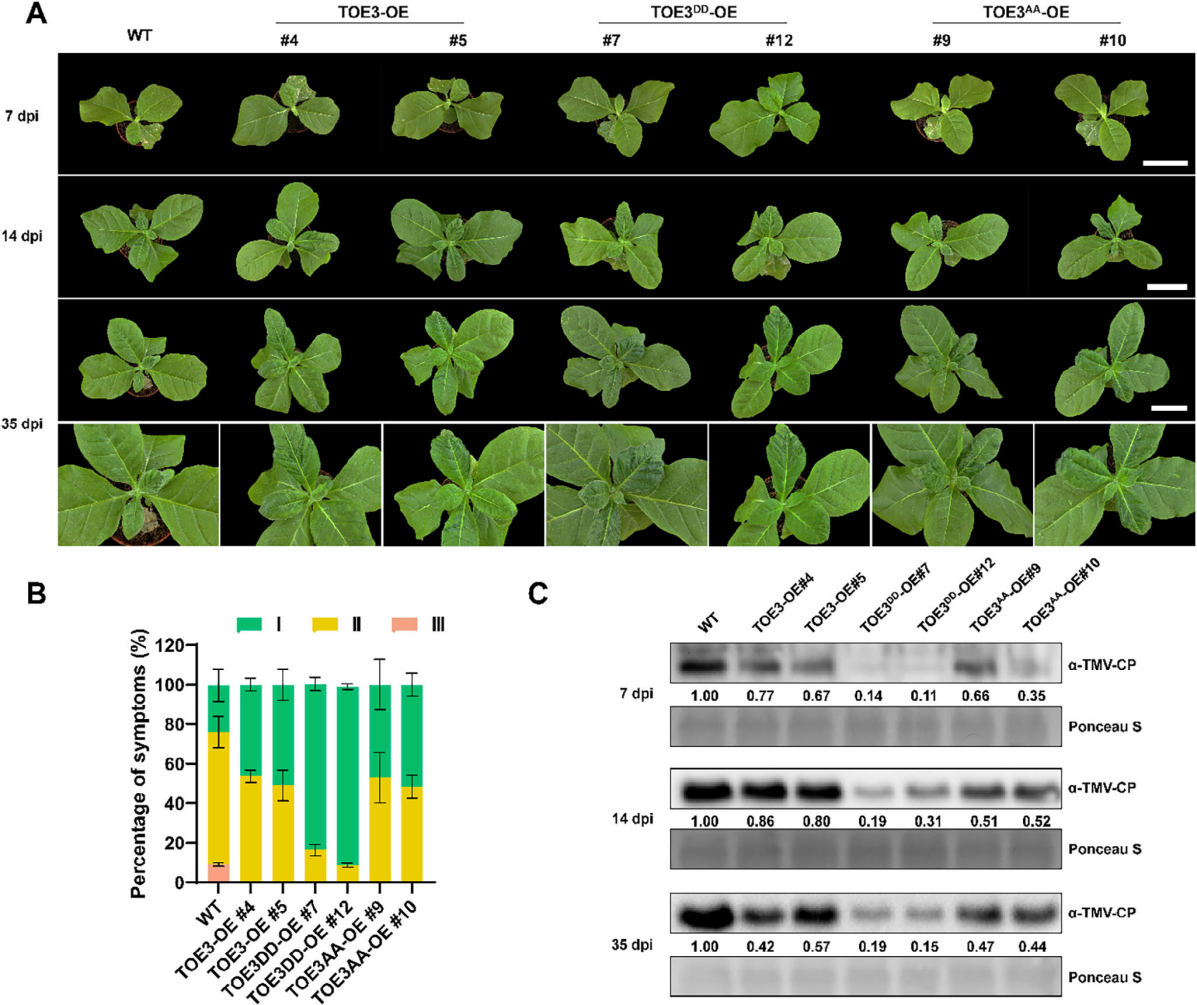
Overexpression of phosphorylated TOE3 enhances tobacco antiviral resistance. A) Disease symptoms of WT, TOE3‐OE, TOE3^DD^‐OE, and TOE3^AA^‐OE tobacco plants at 7, 14, and 35 dpi. Scale bars = 6 cm. B) The symptom percentages of TMV‐infected WT, TOE3‐OE, TOE3^DD^‐OE, and TOE3^AA^‐OE plants with different disease symptom grades at 35 dpi. Data are presented as means ± SD from three independent experiments. C) WB analysis of TMV‐CP accumulation in WT, TOE3‐OE, TOE3^DD^‐OE, and TOE3^AA^‐OE plants at 7, 14, and 35 dpi using anti‐TMV‐CP antibody.

To investigate the antiviral function of phosphorylated TOE3, we generated *TOE3^DD^*‐overexpressing (TOE3^DD^‐OE) and *TOE3^AA^*‐overexpressing (TOE3^AA^‐OE) transgenic *N. tabacum* plants. The WB results showed that TOE3^DD^‐OE plants accumulated more TOE3‐FLAG than TOE3‐OE and TOE3^AA^‐OE tobacco plants (Figure S10, Supporting Information). Then, WT, TOE3‐OE, TOE3^DD^‐OE, and TOE3^AA^‐OE seedlings (28‐day‐old) were inoculated with TMV, respectively. Notably, compared to WT plants, milder symptoms with more green tissue were observed in TOE3‐OE plants. Importantly, both TOE3^DD^‐OE#7 and TOE3^DD^‐OE#12 exhibited more green recovery tissues compared to TOE3‐OE and TOE3^AA^‐OE plants (**Figure** **4**A,B). Consistent with these phenotypic observations, the accumulation of TMV‐CP proteins was substantially decreased in TOE3^DD^‐OE plants (Figure 4C). These results indicate that phosphorylation of TOE3 enhances the antiviral resistance of tobacco. Our above results indicated that the degradation rate of TOE3 in CK2αL‐OE#1 extract was significantly slower than that in WT extract (Figure 3B), which suggested that there might be an antiviral mechanism by which CK2αL protects TOE3 from degradation. However, the mechanism of TOE3 degradation remains unclear.

In order to investigate potential TOE3‐degrading agents, we assessed our Y2H screen results for TOE3, finding that TOE3 might interact with a partial fragment of the F‐box/kelch‐repeat protein At3g23880‐like (FBXL1) (Table S1, Supporting Information). *FBXL1* from *N. sylvestris* (*FBXL1‐S*) and *FBXL1* from *N. tomentosiformis* (*FBXL1‐T*) are two homoeologous genes of *FBXL1* in *N. tabacum*. The CDS sequences of *FBXL1‐S* and *FBXL1‐T* showed high identity (92.70%) (Figure S11A,B, Supporting Information). Given the higher expression level of *FBXL1‐S* in tobacco (Figure S11C, Supporting Information),^[^ ^28^ ^]^ FBXL1‐S was selected for further study and was represented by FBXL1 for the smoothness and convenience of description. FBXL1, localizing in the nucleus, cytoplasm, and cell membrane (Figure S11E, Supporting Information), is part of the SKP1–Cullin1–F‐box (SCF) E3 ubiquitin ligase complex through the interaction of its F‐box domain with SKP1 proteins, and specifically mediates the ubiquitination and degradation of substrates.^[^ ^30^, ^31^ ^]^

**Figure 5:**
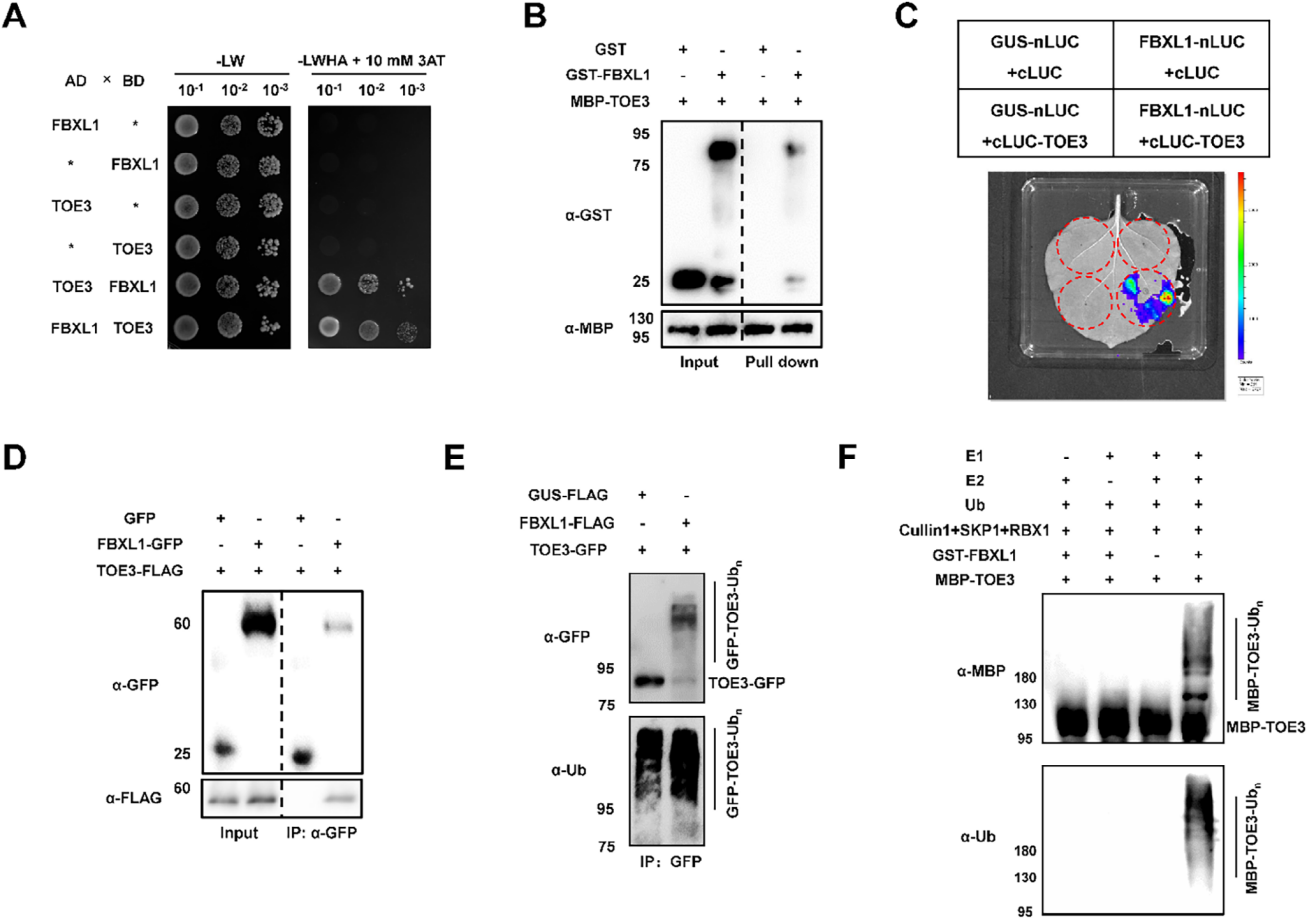
FBXL1 interacts with and ubiquitinates TOE3. A) Y2H assays for the interaction between TOE3 and FBXL1. B) In vitro pull‐down assays showing the interaction between TOE3 and FBXL1. MBP‐TOE3 and GST‐FBXL1 were detected by WB analysis using anti‐MBP and anti‐GST antibodies, respectively. C) Split‐luciferase complementation (SLC) assays of the interaction between TOE3 and FBXL1 *in planta*. Pairs of SLC constructs were co‐infiltrated into the leaves of *N. benthamiana*. At 3 dpa, the LUC luminescence signal was detected. D) Co‐IP assays for the interaction between CK2αL and TOE3 in vivo. WB analysis was detected using anti‐FLAG and anti‐GFP antibodies, respectively. E) FBXL1 ubiquitinated TOE3 in vivo. Proteins were extracted from *N. benthamiana* leaves and were subjected to IP using anti‐GFP beads. WB analysis was then performed to detect the native and ubiquitinated forms of TOE3‐GFP using anti‐GFP and anti‐Ub antibodies, respectively. F) In vitro ubiquitination assays showing ubiquitination of TOE3 by FBXL1. Recombinant MBP‐TOE3 was incubated with E1, E2, ubiquitin, His‐SKP1, His‐Cullin1, and His‐RBX1 in the presence of GST‐FBXL1 at 30 °C for 5 h. WB analysis was performed to detect the native and ubiquitinated MBP‐TOE3 using anti‐MBP and anti‐Ub antibodies, respectively.

To determine whether FBXL1 is involved in regulating the function of TOE3, we performed a Y2H assay, and confirmed that FBXL1 interacted with TOE3 in yeast cells (**Figure** **5**A). We then employed a pull‐down assay to test whether FBXL1 and TOE3 interact with each other in vitro. We first expressed and purified Glutathione S‐Transferase (GST), GST‐FBXL1, and MBP‐TOE3 proteins in vitro. Then, we used amylose resin beads to bind the MBP‐TOE3 proteins, and incubated them with GST or GST‐FBXL1. WB analysis using anti‐MBP and anti‐GST antibodies confirmed that FBXL1 interacted with TOE3 in vitro (Figure 5B). We also examined this interaction in vivo using split‐luciferase complementation (SLC) assays in *N. benthamiana* leaves. FBXL1 was fused to the N‐terminal of luciferase protein (FBXL1‐nLUC), and TOE3 was fused to the C‐terminal of LUC (cLUC‐TOE3). Co‐expression of FBXL1‐nLUC and cLUC‐TOE3 resulted in strong luminescent signals, suggesting that FBXL1 interacted with TOE3 in vivo (Figure 5C). This interaction in vivo was further confirmed by Co‐IP assays, where we detected TOE3‐FLAG in the immunoprecipitated FBXL1‐GFP, but not in the GFP control precipitation (Figure 5D). Our results demonstrate that FBXL1 directly interacts with TOE3 both in vitro and in vivo.

To investigate the effect of FBXL1 on the stability of TOE3, GUS‐FLAG, and FBXL1‐FLAG were each co‐infiltrated into *N. benthamiana* leaves with TOE3‐GFP constructs (Figure S12A, Supporting Information). By observing the GFP fluorescence, we found that the GFP signal of TOE3‐GFP was clearly weaker in FBXL1‐overexpressing leaves compared to that in the control leaves (Figure S12B, Supporting Information). The fluorescence intensity of TOE3‐GFP in *FBXL1*‐overexpressing leaves was significantly lower than that in the control leaves (Figure S12C, Supporting Information). These findings demonstrate that overexpression of *FBXL1* promotes the degradation of TOE3. Furthermore, in vivo ubiquitination assays were performed to detect the ubiquitination level of TOE3‐GFP in *N. benthamiana* leaves with or without FBXL1‐FLAG. TOE3‐GFP protein was immunoprecipitated using GFP agarose beads after protein extraction. Subsequently, WB analysis was performed using anti‐GFP and anti‐Ub antibodies, and showed a smear of slowly migrating polyubiquitinated TOE3‐GFP in the presence of FBXL1‐FLAG (Figure 5E). In contrast, the native TOE3‐GFP was almost undetectable using anti‐GFP antibody in the addition of FBXL1‐FLAG (Figure 5E). Next, an in vitro ubiquitination assay was further performed to test whether FBXL1 ubiquitinates TOE3. The results showed that incubation of MBP‐TOE3 and GST‐FBXL1 yields poly‐ubiquitinated chains of MBP‐TOE3 only in the presence of E1, E2, SKP1, Cullin1, RBX, and Ub (Figure 5F). Taken together, our results suggest that FBXL1 can ubiquitinate TOE3 in vitro and in vivo, thus promoting the degradation of TOE3.

### 2.6 FBXL1 Facilitates TMV Infection through Ubiquitination‐Degradation of TOE3

**Figure 6:**
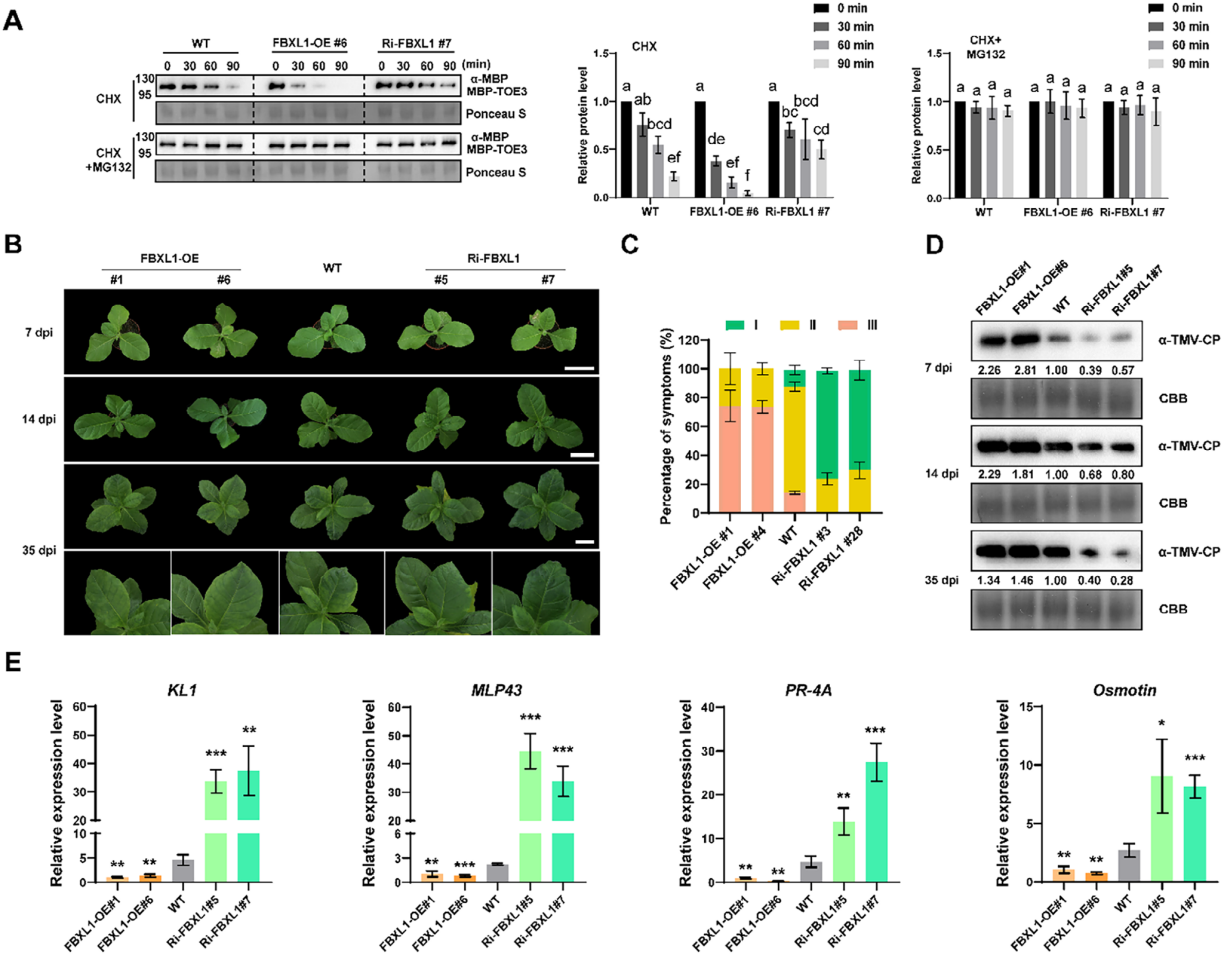
FBXL1 degrades TOE3 through the 26S proteasome to promote viral infection. A) Cell‐free protein degradation assays showing the changes of recombinant MBP‐TOE3 in the extracts from WT, FBXL1‐OE#6, and Ri‐FBXL1#7 seedlings. WB analysis was performed using an anti‐MBP antibody. The relative MBP‐TOE3 abundance was quantified, and the initial protein levels before treatment were normalized to 1. Data are presented as means ± SD from three independent experiments. Statistical analysis was performed using one‐way ANOVA with Tukey's multiple‐comparison test (different letters represent significantly different groups; *P* <  0.05). B) The disease symptoms of FBXL1‐OE, WT, and Ri‐FBXL1 tobacco plants at 7, 14, and 35 dpi. Scale bars = 6 cm. C) The symptom percentages of TMV‐infected FBXL1‐OE, WT, and Ri‐FBXL1 plants with different disease symptom grades at 35 dpi. Data are presented as means ± SD from three independent experiments. D) WB analysis of TMV‐CP accumulation in FBXL1‐OE, WT, and Ri‐FBXL1 plants at 7, 14, and 35 dpi using anti‐TMV‐CP antibody. D) Expression levels of defense‐related genes activated by TOE3, including *KL1*, *MLP43*, *PR‐4A*, and *Osmotin*, in FBXL1‐OE, WT, and Ri‐FBXL1 plants at 7 dpi. Data are presented as means ± SD from three independent experiments. Significant differences are determined using Student's *t*‐test (^*^ *P* < 0.05, ^**^ *P* < 0.01, ^***^ *P* < 0.001).

Then, we generated *FBXL1*overexpressing (FBXL1‐OE) transgenic *N. tabacum* lines (Figure S13A,B, Supporting Information). For FBXL1‐T also interacted with TOE3, and the CDS sequences of *FBXL1‐S* and *FBXL1‐T* show high identity (92.70%) (Figures S13C and S11A, Supporting Information), RNAi‐mediated knockdown transgenic *N. tabacum* lines (Ri‐FBXL1) were generated to silence the two homoeologous genes of *FBXL1* (Figure S13D,E, Supporting Information). To further investigate the degradation of TOE3 by FBXL1, we performed cell‐free protein degradation assays using recombinant MBP‐TOE3 incubated with protein extracts prepared from WT, FBXL1‐OE#6, or Ri‐FBXL1#7 plants. The results indicated that the degradation rate of MBP‐TOE3 was slower in Ri‐FBXL1#7 extract and faster in FBXL1‐OE#6 extract compared to WT extract (**Figure** **6**A). Interestingly, the addition of the proteasome inhibitor MG132 inhibited the degradation of MBP‐TOE3 (Figure 6A). These results demonstrate that FBXL1 significantly promotes the degradation of TOE3 via the 26S proteasome.

To study the function of FBXL1 upon TMV infection, WT, FBXL1‐OE, and Ri‐FBXL1 seedlings (28‐day‐old) were then inoculated with TMV. The results showed that FBXL1‐OE lines exhibited more severe etiolation and more interveinal chlorotic tissue compared to WT plants at 35 dpi. However, milder symptoms with more green tissue were observed in Ri‐FBXL1 lines compared to the WT plants at 35 dpi (Figure 6B,C). The WB results further confirmed that the virus titers in FBXL1‐OE lines were much higher than those in the WT plants at 7, 14, and 35 dpi, while virus accumulation levels in Ri‐FBXL1 plants were lower than those in the WT plants (Figure 6D). RT‐qPCR analysis demonstrated that the expression level of defense‐related genes was down‐regulated in FBXL1‐OE plants and up‐regulated in Ri‐FBXL1 plants (Figure 6E), displaying a trend opposite to that in TOE3‐OE, CR‐TOE3,^[^ ^9^ ^]^ CK2αL‐OE, and Ri‐CK2αL lines (Figure 3F). These results suggest that overexpression of *FBXL1* promotes TMV infection in *N. tabacum* plants.

To further elucidate the genetic relationship between FBXL1 and TOE3 in tobacco antiviral immunity, we crossed Ri‐FBXL1#7 with CR‐TOE3#68. Next, 28‐day‐old seedlings of WT, Ri‐FBXL1#7, CR‐TOE3#68, and the resultant Ri‐FBXL1 CR‐TOE3 plants were inoculated with TMV. The results showed that the leaves of Ri‐FBXL#6 exhibited more green recovery tissue and milder symptoms compared to those in WT, CR‐TOE3#68, and Ri‐FBXL1 CR‐TOE3 at 35 dpi. However, silencing of *FBXL1* did not attenuate the symptom of severe chlorosis in CR‐TOE3 (Figure S14A,B, Supporting Information). WB analysis also demonstrated that virus titers were not decreased in Ri‐FBXL1 CR‐TOE3 compared to those in CR‐TOE3#68 plants (Figure S14C, Supporting Information). Taken together, these results indicate that FBXL1 ubiquitinates and degrades TOE3 to negatively regulate antiviral defense.

### 2.7 Phosphorylated TOE3 shows Lower Levels of FBXL1‐Mediated Degradation

After finding that CK2αL enhances the stability of TOE3 (Figure 3A,B), we hypothesized that CK2αL might interfere with FBXL1‐mediated TOE3 degradation. To investigate this hypothesis, we first performed an in vitro competitive binding assay to study whether CK2αL disrupts the interaction between FBXL1 and TOE3. The results showed that increased amounts of GST‐CK2αL did not impair the interaction between MBP‐FBXL1 and TOE3‐FLAG (Figure S15, Supporting Information).

**Figure 7:**
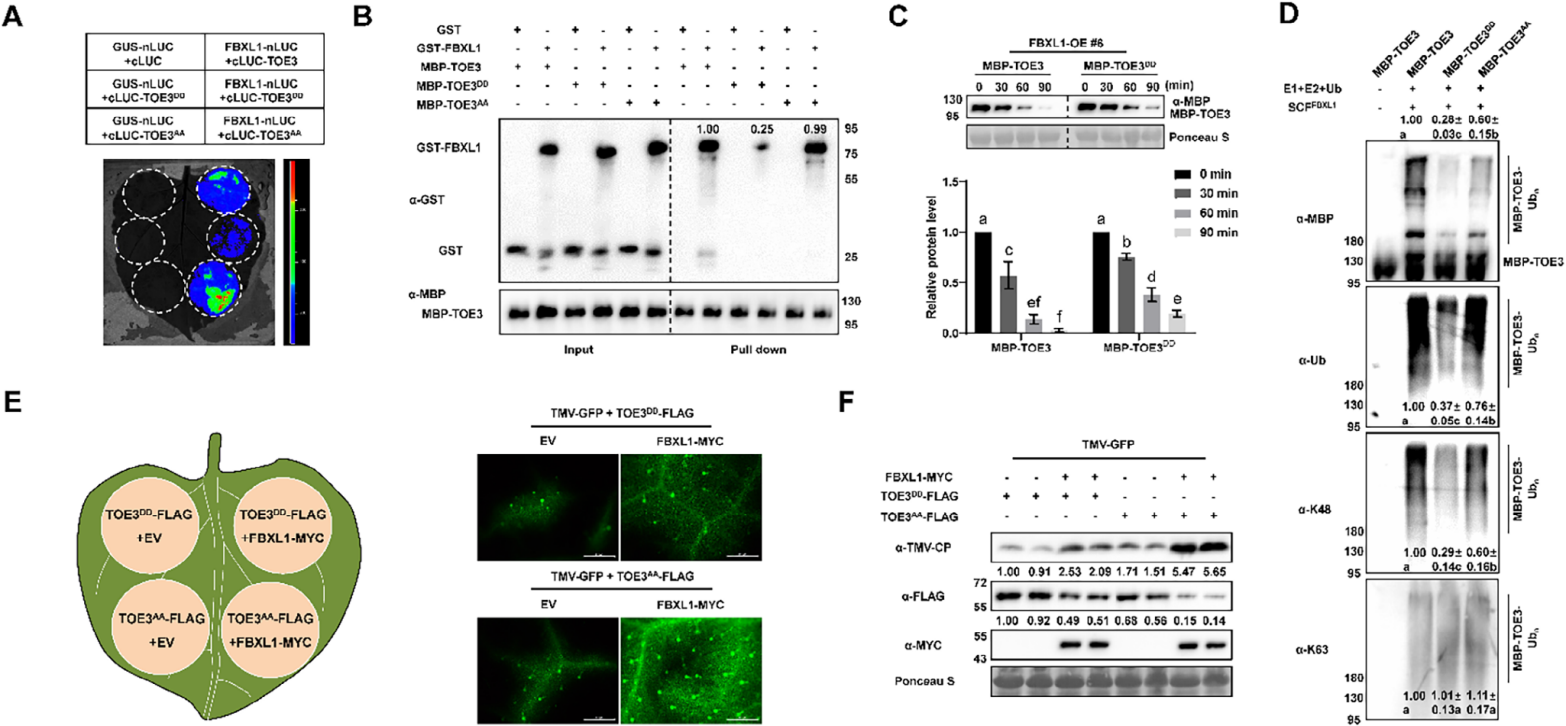
FBXL1 tends to degrade nonphosphorylated TOE3 to facilitate viral infection. A) SLC assays of the interaction between FBXL1 and TOE3, TOE3^DD^, or TOE3^AA^. B) In vitro pull‐down assays of the interaction between GST‐FBXL1 and MBP‐TOE3, MBP‐TOE3^DD^, or MBP‐TOE3^AA^. WB analysis was performed using anti‐GST and anti‐MBP antibodies, respectively. C) Cell‐free protein degradation assays showing the changes in MBP‐TOE3 and MBP‐TOE3^DD^ in FBXL1‐OE#7 extracts. WB analysis was performed using an anti‐MBP antibody. The relative amounts of MBP‐TOE3 and MBP‐TOE3^DD^ were quantified, and the initial protein levels before treatment were set to 1. Data are presented as means ± SD from three independent experiments. Statistical analysis was performed using one‐way ANOVA with Tukey's multiple‐comparison test (different letters represent significantly different groups; *P* <  0.05). D) In vitro FBXL1‐mediated ubiquitination assays of TOE3, TOE3^DD^, and TOE3^AA^. Recombinant MBP‐TOE3, MBP‐TOE3^DD^, or MBP‐TOE3^AA^ proteins were incubated with E1, E2, ubiquitin, His‐SKP1, His‐Cullin1, and His‐RBX1 in the presence of GST‐FBXL1 at 30 °C for 5 h. WB analysis was performed to detect the native and ubiquitinated MBP‐TOE3 using anti‐MBP, anti‐Ub, anti‐lysine 48‐linked (K48) Ub, and anti‐K68 Ub antibodies, respectively. The protein levels of MBP‐TOE3‐Ub_n_ and MBP‐TOE3 in the solution of E1, E2, Ub, SCF^FBXL1^, and MBP‐TOE3 were normalized to 1. Data are presented as means ± SD from three independent experiments. Statistical analysis was performed using one‐way ANOVA with Tukey's multiple‐comparison test (different letters represent significantly different groups; *P* <  0.05). E) FBXL1 preferred to degrade TOE3^AA^ over TOE3^DD^, thus facilitating TMV infection. TMV‐GFP infectious clones, and TOE3^DD^‐FLAG/TOE3^AA^‐FLAG were co‐expressed in *N. benthamiana* leaf halves treated with or without FBXL1‐MYC. The GFP fluorescence expressed from the TMV replicon was examined and photographed under a fluorescence microscope at 2 dpi. EV, 35S: MYC empty vector. Scale bars = 500 µm. F) WB analysis of TMV replicon, FBXL1‐MYC, TOE3^DD^‐FLAG, and TOE3^AA^‐FLAG protein accumulation shown in E) using anti‐TMV‐CP, anti‐MYC, and anti‐FLAG antibodies, respectively.

Next, we further tested whether phosphorylation of TOE3 by CK2αL would alter the binding affinity of TOE3 with FBXL1. The results of SLC assays and in vitro pull‐down assays indicated that FBXL1 preferred to bind with the nonphosphorylated TOE3 (TOE3^AA^) compared to the phosphorylated TOE3 (TOE3^DD^) (**Figure** **7**A,B). Thus, we speculated that phosphorylation of TOE3 may protect it from degradation by FBXL1. To verify this hypothesis, MBP‐TOE3 and MBP‐TOE3^DD^ were each incubated with FBXL1‐OE#6 extract. The results showed that MBP‐TOE3^DD^ exhibited a significantly slower degradation rate than MBP‐TOE3 (Figure 7C). These findings suggest that phosphorylation of TOE3 by CK2αL significantly reduces its degradation rate due to the decreased interaction intensity between TOE3^DD^ and FBXL1.

To assess the FBXL1‐mediated ubiquitination levels of phosphorylated TOE3 and nonphosphorylated TOE3, the in vitro ubiquitination assay was carried out using MBP‐TOE3, MBP‐TOE3^DD^, and MBP‐TOE3^AA^. The results indicated that MBP‐TOE3 was strongly ubiquitinated by GST‐FBXL1 (Figure 7D), which was consistent with the above result (Figure 5F). Importantly, the ubiquitination level of MBP‐TOE3^DD^ was significantly weaker than that of MBP‐TOE3^AA^ and MBP‐TOE3 (Figure 7D), suggesting that CK2αL‐mediated phosphorylation of TOE3 reduces the ubiquitination level of TOE3.

Proteins marked by lysine 48 (K48)‐linked polyubiquitination are usually degraded by the 26S proteasome, while other polyubiquitin chains, such as K63, usually regulate other protein properties.^[^ ^32^ ^]^ Thus, we detected both K48‐ and K63‐linked polyubiquitination signals for the above three forms of MBP‐TOE3. The results showed that the intensity of K48‐linked ubiquitination of MBP‐TOE3^DD^ was significantly weaker than that of MBP‐TOE3 and MBP‐TOE3^AA^. In contrast, the K63‐linked ubiquitination of MBP‐TOE3, MBP‐TOE3^DD^, and MBP‐TOE3^AA^ was present at detectable levels, with no significant differences between them (Figure 7D). These results demonstrate that FBXL1 facilitates the ubiquitination of TOE3 through K48‐ and K63‐linked chains, with a preference for K48‐linked ubiquitination. Importantly, phosphorylated TOE3 inhibits K48‐linked polyubiquitination, thus reducing its own degradation.

Finally, we co‐expressed TMV‐GFP infectious clones with constructs containing TOE3^DD^‐FLAG/TOE3^AA^‐FLAG in *N. benthamiana* leaves treated with or without FBXL1‐MYC (Figure 7E). The results showed that overexpression of TOE3^DD^‐FLAG resulted in higher TOE3 levels compared to TOE3^AA^‐FLAG. In addition to FBXL1‐MYC, the degradation rate of TOE3^AA^‐FLAG was much higher than that of TOE3^DD^‐FLAG. The value of the TOE3 degradation ratio in TOE3^AA^‐FLAG was ≈4, while the value of that in TOE3^DD^‐FLAG was only ≈2. Importantly, compared to the lower TOE3 level and higher viral accumulation in the TOE3^AA^‐FLAG + FBXL1‐MYC group, co‐expression of TOE3^DD^‐FLAG and FBXL1‐MYC enhanced TOE3 stability and inhibited TMV accumulation (Figure 7E,F). Taken together, our results demonstrate that phosphorylation of TOE3 by CK2αL blocks its degradation mediated by FBXL1 through decreasing the binding affinity between phosphorylated TOE3 and FBXL1, thus enhancing the antiviral resistance.

## 3 Discussion

AP2 transcription factors participate in various plant processes, including growth,^[^ ^33^, ^34^ ^]^ floral architecture,^[^ ^35^ ^]^ vegetative phase change,^[^ ^36^, ^37^ ^]^ and responses to abiotic stresses.^[^ ^38^, ^39^, ^40^ ^]^ Recent studies have reported the essential roles of AP2 transcription factors in response to biotic stress.^[^ ^41^ ^]^ In *Arabidopsis*, TOE1 and TOE2 inhibit the transcription of *FLAGELLIN‐SENSING2* (*FLS2*) to regulate the ontogeny of plant innate immunity.^[^ ^41^, ^42^ ^]^ Our previous study demonstrated that TOE3 improves the resistance of tobacco to TMV by up‐regulating the expression levels of defense‐related genes, such as *kirola‐like 1* (*KL1*) and *major latex protein‐like protein 43* (*MLP43*).^[^ ^9^ ^]^ Interestingly, MLP43 has been reported to enhance plant antiviral effects against multiple viruses, such as TMV, cucumber mosaic virus (CMV), and potato virus Y (PVY),^[^ ^43^ ^]^ which suggests the potential of TOE3 to mediate broad‐spectrum resistance against multiple RNA viruses by up‐regulating *MLP43* expression. To validate this hypothesis, we inoculated wild‐type, *TOE3*‐overexpressing, and *TOE3*‐knockout transgenic *N. tabacum* plants with either chili veinal mottle virus (ChiVMV) or CMV. The results confirmed that TOE3 confers broad‐spectrum resistance to viruses, including TMV,^[^ ^9^ ^]^ CMV, and ChiVMV (Figure S16, Supporting Information). In this study, the results of Y2H screening showed that PYL4, known as an ABA receptor, is also a potential protein that interacted with TOE3 (Table S1, Supporting Information). ABA has been reported to participate in regulating plant antiviral defense.^[^ ^44^ ^]^ Thus, we speculated that TOE3 might influence the ABA signaling pathway to regulate tobacco defense, which needs to be further investigated in the future.

Several studies have also shown that TOE3 positively regulates plant tolerance to abiotic stress. For example, overexpression of *TOE3* in tobacco and sweet potato has been found to enhance tolerance to salt, drought, and oxidative stresses by maintaining reactive oxygen species (ROS) homeostasis.^[^ ^45^ ^]^ In herbaceous peony, TOE3 activates the transcription of *tryptophan decarboxylase* (*TDC*) to increase the production of phytomelatonin and thus enhance high‐temperature stress tolerance.^[^ ^46^ ^]^ However, in this study, we found that overexpression of *TOE3* reduces the drought tolerance of tobacco (Figure S17, Supporting Information). To mitigate the negative effect of *TOE3* overexpression on drought tolerance, there is a feasible strategy to optimize the promoter of *TOE3* by inserting a TMV‐inducible cis‐elements in the promoter of *TOE3* and deleting the drought‐inducible motif. Thus, the expression of *TOE3* is induced by TMV infection, but is not activated by drought stress. This strategy could enhance the antiviral advantages of TOE3 while alleviating its drought‐sensitive deficiency. In addition, our previous work has shown that overexpression of *TOE3* delays vegetative phase change and promotes the growth of tobacco plants.^[^ ^9^ ^]^ Vegetative phase change is a major developmental switch in the life cycle of flowering plants, where metabolic expenditure is shifted from vegetative growth to reproductive development. Tobacco is one of the crops that primarily harvest nutrient organs, delaying flowering can promote prolonged growth before switching to the reproductive phase, enabling the plants to allocate more energy to the growth of leaves, thereby increasing leaf yield. Overexpression of *TOE3* in tobacco may provide a strategy to delay vegetative phase change and increase vegetative biomass production. Overall, overexpression of *TOE3* could promote the growth of tobacco and enhance broad‐spectrum antiviral resistance, while reducing tobacco tolerance to drought stress.

Previous studies have reported that phosphorylation is associated with the proteasomal degradation of AP2 transcription factors. For example, a recent study revealed that BIN2‐mediated phosphorylation of the TOE1 T124 residue delays vegetative phase change by destabilizing TOE1.^[^ ^47^ ^]^ Moreover, a PEST sequence containing a potential phosphorylation site was located in the C‐terminal region of the AP2 transcription factor WRINKLED1 (WRI). Removal of the PEST motif or mutation of the phosphorylation site increased the stability of AtWRI1.^[^ ^48^ ^]^ Another study demonstrated that SUCROSE NONFERMENTATION1‐RELATED KINASE10 (KIN10)‐induced phosphorylation of WRI1 accelerated the proteasomal degradation of WRI1, with the two phosphorylation sites of WRI1 by KIN10 (T70 and S166) being located on the AP2 DNA‐binding domains.^[^ ^49^ ^]^ However, in the present study, the mass spectrometry analysis and in vitro phosphorylation assays confirmed that both phosphorylation sites (S58 and T128) of TOE3 are located on the N‐terminal region, but not the AP2 domain or the C‐terminal region (Figure 2B–D), indicating its phosphorylation‐caused degradation mechanisms might be distinct. Our results showed that phosphorylated TOE3 was more stable than nonphosphorylated TOE3 in the extracts of *FBXL1*‐overexpressed plants (Figure 7C), and exhibited a lower level of ubiquitination (Figure 7D). Furthermore, TOE3 incubated in extracts of *CK2αL*‐overexpressed plants exhibited significantly slower degradation rates than those of wild‐type plants or *CK2αL*‐knockdown plants (Figure 3B), indicating that the phosphorylation of TOE3 by CK2αL inhibited the FBXL1‐mediated ubiquitination degradation of TOE3. We also elucidated the mechanism by which CK2αL suppresses the degradation of TOE3, which is due to FBXL1's preference for binding to nonphosphorylated TOE3 over phosphorylated TOE3 (Figure 7A,B). Therefore, CK2αL enhances the resistance of tobacco by stabilizing TOE3. Interestingly, the two AP2 domains of TOE3 are both DNA‐binding domains to recognize their target motif.^[^ ^50^ ^]^ Thus, phosphorylation of the N‐terminal—but not the AP2 DNA‐binding domains—of TOE3 by CK2αL exhibited no significant effects on the transcriptional regulation activity of TOE3 (Figure S6B, Supporting Information). Moreover, we found that phosphorylation site S58 was conserved in *Solanaceae* species such as potato, tomato, pepper, and other crops (Figure 2D), indicating that this phosphorylation site may play a key role in stabilizing TOE3 in these crops and merits further research in the future.

CK2β subunits have been reported to be responsible for the specificity of the CK2 holoenzyme toward downstream substrates.^[^ ^51^, ^52^, ^53^ ^]^ However, several studies have also demonstrated that CK2α can directly interact with and phosphorylate viral proteins. For instance, CK2α and β subunits bind to the N terminus of Epstein‐Barr virus (EBV) early protein EB2 either individually or—more efficiently—as a complex, thereby controlling EB2‐mediated production of infectious EBV virions through CK2‐induced phosphorylation.^[^ ^54^ ^]^ In both monocots and dicots, CK2α plays a critical role in regulating the movement of barley stripe mosaic virus (BSMV) by phosphorylating the TGB1 protein of BSMV.^[^ ^55^ ^]^ These reports indicated the potential of CK2 in regulating antiviral defense by modulating viral proteins. Besides regulating viral proteins, Kwon and colleagues confirmed that CK2α subunits directly bind to and phosphorylate their substrate protein Pseudo‐response regulator 37 (PRR37) in long‐day‐dependent floral repression in rice and other plants.^[^ ^56^ ^]^ Similarly, in the present study, CK2αL showed the ability to directly phosphorylate TOE3 without the cooperation of other CK2 subunits (Figure 2A; Figures S2, S3, and S4, Supporting Information), and was identified as a positive regulatory factor of tobacco antiviral immunity (Figure 3C–E).

**Figure 8:**
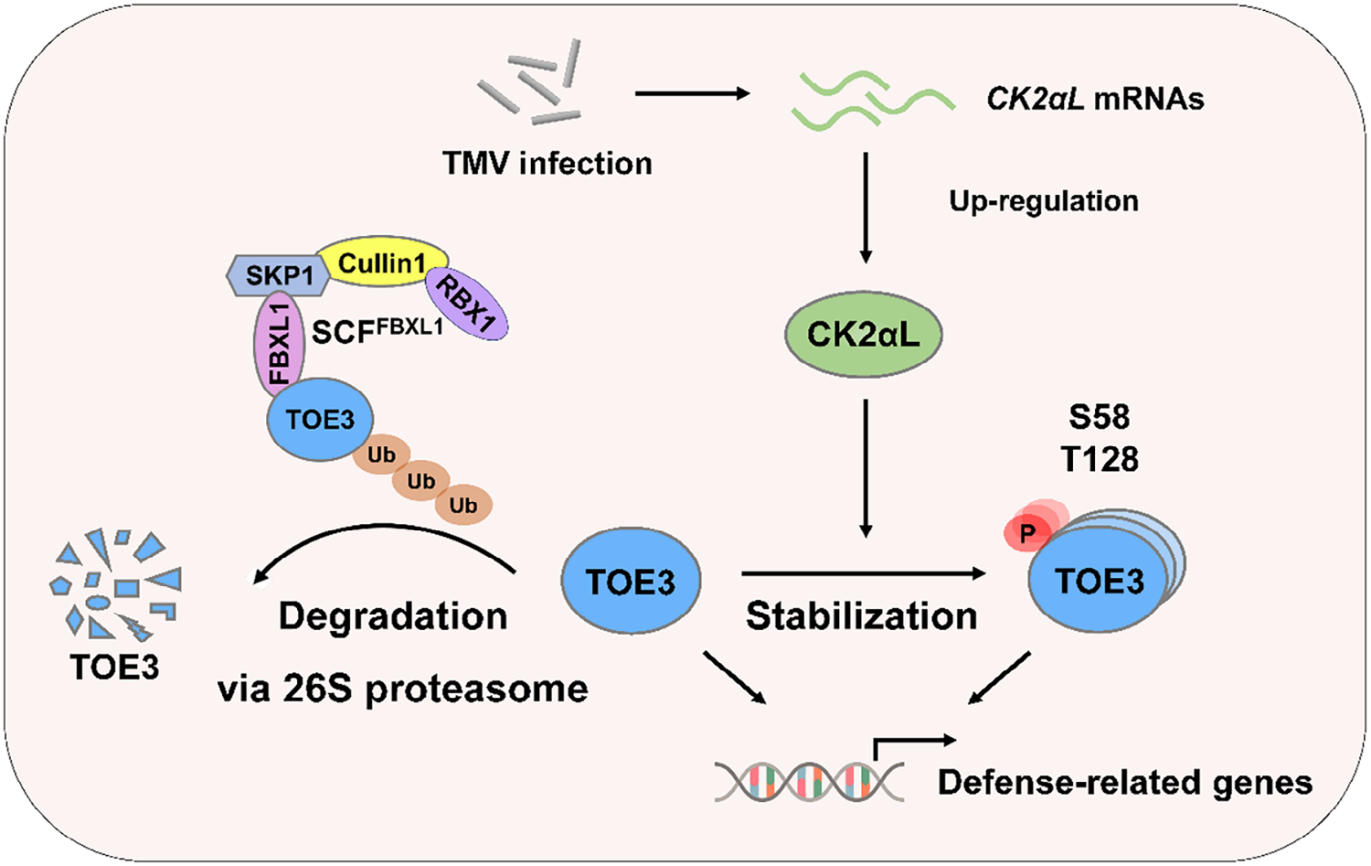
A proposed working model of the post‐translational modifications of TOE3 in the regulation of antiviral immunity. After TMV infection, the transcription of *CK2αL* is up‐regulated. CK2αL interacts with TOE3 and phosphorylates it at S58 and T128. Nonphosphorylated TOE3 tends to bind with FBXL1 and is degraded via the 26S proteasome, whereas phosphorylated TOE3 is more stable due to the lower binding affinity to FBXL1. Thus, the accumulated TOE3 activates the expression of downstream defense‐related genes to enhance tobacco antiviral immunity.

In summary, our results revealed a mechanism by which phosphorylation and ubiquitination of TOE3 co‐regulate the resistance of tobacco against TMV infection (**Figure** **8**). After TMV infection, increasing CK2αL leads to the subsequent phosphorylation of TOE3. This CK2αL‐mediated phosphorylation results in the inhibition of SCF^FBXL1^‐mediated degradation of TOE3 via 26S proteasome due to the decreased affinity between phosphorylated TOE3 and FBXL1. The stable TOE3 increases the expression levels of defense‐related genes, thus improving the resistance of tobacco to TMV. Our study provides new insights into the roles of post‐translational modifications of TOE3 in regulating plant antiviral immunity, and highlights candidate genes for creating crop varieties with coordinated vegetative growth and broad‐spectrum antiviral resistance.

## 4 Experimental Section

### Plant Materials and Virus Inoculation

*N. tabacum* cultivar “NC89” and *N. benthamiana* were used in this study. All plants were grown in a greenhouse at 24 ± 1 °C under a 16 h/8 h light/dark photoperiod. TMV, CMV, and ChiVMV were used in this study, and these viral isolates were maintained in 0.02 m phosphate‐buffered saline (0.02 m PBS, pH 6.8) at −80 °C. The virus was rub‐inoculated into the leaves of *N. tabacum* and *N. benthamiana* as described previously;^[^ ^57^ ^]^ PBS inoculation was used as the control. For agrobacterium‐mediated inoculation of TMV‐GFP infectious clones, *Agrobacterium tumefaciens* harboring TMV‐GFP infectious clones was infiltrated into leaves of *N. benthamiana*. Leaf samples were collected independently and immediately frozen in liquid nitrogen and stored at −80 °C for DNA, RNA, or protein extraction.

### Vector Construction

The ClonExpress II One Step Cloning Kit (Vazyme, Nanjing, China) was used for plasmid construction. The full‐length CDS sequence of *NtCK2αL* and *NtFBXL1* was cloned and inserted into pCAMBIA1307 and pCAMBIA1300 to produce NtCK2αL‐FLAG, NtFBXL1‐FLAG, NtCK2αL‐GFP, and NtFBXL1‐GFP fusion proteins driven by the cauliflower mosaic virus (CaMV) 35S promoter. Fragments of *CK2αL* and *FBXL1* for RNA interference were obtained by RT‐PCR and inserted into the intron‐containing pRNAi‐LIC vector as described previously.^[^ ^58^ ^]^ The primers used for RT‐PCR are listed in Table S2, Supporting Information.

### Agrobacterium Infiltration and Genetic Transformation

The recombinant vectors for gene overexpression and RNA interference were introduced into *A. tumefaciens* strain GV3101. For transient expression assays, agrobacterium strain cultures harboring vectors were infiltrated into the leaves of *N. benthamiana* plants. Tobacco genetic transformation was performed using the agrobacterium‐mediated leaf disc transformation method.^[^ ^59^ ^]^ Transformed tissue was selected by culturing calli and then regenerated. Hygromycin B was used to screen the positive transformed tobacco plants. The overexpression of transgenic *N. tabacum* lines was analyzed by WB and RT‐qPCR. The RNAi‐mediated knockdown of transgenic *N. tabacum* lines was analyzed by PCR and RT‐qPCR. The primers used for PCR and RT‐qPCR are listed in Table S2, Supporting Information.

### Y2H Assays

Y2H assays were performed as described previously.^[^ ^60^ ^]^ Briefly, full‐length CDS of *CK2αL* and *FBXL1* were cloned into the pGADT7 vector as the prey. The full‐length sequence of *TOE3* was cloned into the pGBKT7 construct as the bait. Each pair of bait and prey constructs was co‐transformed into the yeast strain AH109. Then, the strains were cultured on Double DO supplement medium (‐Leu/‐Trp, ‐LW) (Clontech, California, USA) for 3 days, and shifted onto Quadruple DO supplement (‐Leu/Trp/Ade/His, ‐LWHA) (Clontech, California, USA) to test for any possible interaction.

### BiFC Assays

A bimolecular fluorescence complementation assay was conducted using the N‐ and C‐termini of YFP as described previously.^[^ ^61^ ^]^ In brief, the coding regions of *CK2αL* and *FBXL1* were fused into a pXY103 vector carrying the N‐terminal of YFP, and *TOE3* was fused into a pXY104 vector carrying the C‐terminal of YFP. Agrobacterium cultures carrying a pair of nYFP and cYFP constructs were co‐infiltrated into the leaves of *N. benthamiana*. After 48 h, YFP was excited with a 514‐nm laser line, and YFP signals were detected using a fluorescence microscope (Leica, Heerbrugg, Switzerland).

### In Vitro Pull‐Down Assays

The recombinant constructs were transferred into *E. coli* BL21 competent cells (TsingKe, Beijing, China) and proteins were purified using the appropriate beads as described previously.^[^ ^62^ ^]^ For His pull‐down assay, purified MBP or MBP‐fused CK2αL proteins were incubated with equal amounts of His‐TOE3 beads in pull‐down buffer (20 mm Tris‐HCl [pH 8.0], 150 mm NaCl, 1 mm PMSF, 0.2% Triton X‐100, and 1% protease inhibitor cocktail) at 4 °C for 2 h with gentle rotation. The beads were washed six times using pull‐down buffer and then collected and boiled in 50 µL of 2 × SDS loading buffer for 10 min at 100 °C. Finally, the protein extract was detected by WB analysis using anti‐His or anti‐MBP antibodies (Abcam, Cambridge, UK).

### In Vitro Competitive Pull‐Down Assays

The in vitro competitive pull‐down assay was performed as described previously.^[^ ^63^ ^]^ 5 µg of TOE3‐FLAG mixed with 1, 5, or 10 µg of GST‐FBXL1 was incubated with amylose resin beads containing 10 µg of MBP or MBP‐CK2αL in 500 µL pull‐down buffer (20 mm Tris‐HCl [pH 7.4], 200 mm NaCl, 1 mm PMSF, 0.2% Triton X‐100, 1% protease inhibitor cocktail) at 4 °C for 2 h. The beads were then washed six times with the pull‐down buffer. Proteins were eluted from beads by boiling in 100 °C with 30 µL of 2 × SDS loading buffer. Finally, WB analysis was conducted using anti‐MBP, anti‐GST (Abcam, Cambridge, UK), or anti‐FLAG (MBL, Beijing, China) antibodies.

### Co‐IP Assays

Co‐immunoprecipitation (Co‐IP) was performed as described previously.^[^ ^64^ ^]^ Briefly, agrobacterium suspensions containing TOE3‐FLAG and CK2αL‐GFP were co‐infiltrated into the leaves of *N. benthamiana*. At 3 dpa, the total proteins were extracted and then incubated with FLAG‐agarose beads (Sigma–Aldrich) or GFP‐trap agarose beads (ChromoTek, Martinsried, Germany) in IP buffer (10 mm Tris‐HCl [pH 7.5], 2 mm EDTA, 150 mm NaCl, 0.5% Nonidet P‐40, 1 mm PMSF, and 1% plant protease inhibitor cocktail). After gentle rotation for 2 h, the beads were collected and washed at least five times with IP buffer, and then mixed with 2 × SDS loading buffer. Finally, the protein extracts and coimmunoprecipitated proteins were detected by immunoblot analysis using anti‐FLAG and anti‐GFP (ABclonal, Wuhan, China) antibodies, respectively.

### SLC Assays

The SLC assays were carried out as described previously with some modifications.^[^ ^65^ ^]^ Agrobacterium suspensions containing the pair of nLUC and cLUC constructs were co‐expressed in the leaves of *N. benthamiana* to be examined for protein interaction. The LUC signals were detected using IVIS Lumina III (PerkinElmer, USA) with an exposure time of 2–10 min.

### In Vitro Kinase Assays

The kinase assays were performed as described previously with some modifications.^[^ ^66^ ^]^ For the in vitro kinase assay, each reaction was incubated in 20 µL of kinase buffer (20 mm Tris‐HCl [pH 7.5], 100 mm NaCl, 12 mm MgCl_2_, 10 µm ATP, 1 mm of PMSF, and 1% plant protease inhibitor cocktail). The negative control was treated with λ‐PPase. The samples were incubated at 30 °C for 1 h and then boiled in 2 × SDS loading buffer at 100 °C for 10 min. Phosphorylated proteins were examined using Phos‐tag reagent (NARD Institute, Japan) as described previously.^[^ ^64^ ^]^ In brief, the proteins were separated by 10% SDS–PAGE gels containing 50 µm Phos‐tag reagent and 100 mm MnCl_2_. The separated proteins were then transferred to a PVDF membrane (Millipore, USA) and blotted with anti‐MBP antibody for WB analysis. The phosphorylation level was also detected using another method

In brief, the reaction samples were separated by regular 10% SDS–PAGE gels and transferred to a PVDF membrane. Finally, WB analysis was performed using anti‐phospho‐Ser/Thr (pS/T) antibody (Abcam, Cambridge, UK).

### Mass Spectrometry Analysis

For mass spectrometry analysis, the reaction samples were separated in regular 10% SDS–PAGE gels. Then, the bands containing MBP‐TOE3 were excised after Coomassie blue staining and digested with trypsin. To analyze the peptide fragments and phosphorylation sites, liquid chromatography–tandem mass spectrometry (LC–MS/MS) was performed using Q Exactive Plus (Thermo, USA) (Jingjie PTM BioLabs, Hangzhou, China).

### In Vivo Kinase Assays

For the in vivo kinase assay, the CK2αL‐FLAG and TOE3‐GFP fusion constructs were co‐overexpressed in the leaves of *N. benthamiana*. At 3 dpa, the total proteins were extracted. TOE3‐GFP fusion proteins were then immunoprecipitated with anti‐GFP beads. Finally, the phosphorylated proteins were separated using Phos‐tag gels and detected using an anti‐GFP antibody.

### Dual‐Luciferase Assay

The dual‐luciferase assay was performed as described previously.^[^ ^63^ ^]^ The promoter of *NtKL1* was cloned into the pGreen II 0800‐LUC vector to generate a reporter construct. The reporter construct was co‐transformed with 35S: NtTOE3^DD^ or 35S: NtTOE3^AA^ in *N. benthamiana* leaves according to the dual luciferase assay kit (Vazyme, Nanjing, China) protocol. Firefly and Renilla luciferase signals were detected using the Berthold Centro LB960 luminometer system. The LUC:REN ratio was calculated as the final measurement assay.

### In Vitro Ubiquitination Assay

The in vitro ubiquitination assay was performed as described previously.^[^ ^31^, ^67^ ^]^ The recombinant proteins His‐SKP1, His‐RBX1, and His‐Cullin1 were expressed by *E. coli* BL21 (DE3) cells and purified using Ni‐NTA agarose (Invitrogen, California, USA). Then, the E2‐Ub Conjugation Kit (Abcam, Cambridge, UK) was employed for in vitro ubiquitination. Each reaction mixture (20 µL) contained 50 mm Tris‐HCl (pH 7.4), 10 mm MgCl_2_, 5 mm ATP, 2 mm DTT, 20 mm ZnCl_2_, 250 ng E1 (wheat E1), 200 ng E2 (human E2 UbcH5B), 0.15 µg His‐SKP1, 0.4 µg His‐Cullin1, 0.15 µg His‐RBX1, 0.4 µg GST‐FBXL1, 500 ng MBP‐TOE3, and 5 µg Ub. After incubating at 30 °C for 5 h, MBP‐TOE3 was purified using amylose resin beads, and then WB analysis was used to detect the ubiquitination level using anti‐MBP, anti‐Ub (Abcam, Cambridge, UK), anti‐K48 Ub (Abcam, Cambridge, UK), and anti‐K63 Ub (Abcam, Cambridge, UK) antibodies, respectively.

### In Vivo Ubiquitination Assay

For the in vivo ubiquitination assay, TOE3‐GFP and FBXL1‐FLAG were co‐expressed in the leaves of *N. benthamiana*. After 24 h of expression, the MG132 solution was injected into the leaves. At 3 dpa, the total proteins were extracted and the TOE3‐GFP fusion proteins were then immunoprecipitated with anti‐GFP beads. Finally, the ubiquitination was examined through WB analysis using anti‐GFP and anti‐Ub antibodies, respectively.

### Cell‐Free Protein Degradation Assay

Cell‐free protein degradation assays were performed as described previously.^[^ ^68^ ^]^ In brief, total proteins were extracted from CK2αL and FBXL1 transgenic *N. tabacum* lines with degradation buffer (25 mm Tris–HCl [pH 7.5], 10 mm NaCl, 10 mm MgCl_2_, 0.1% Triton X‐100, 1 mm PMSF, 5 mm DTT, and 10 mm ATP). MBP‐TOE3 (100 ng) was incubated in the protein extracts with/without 100 µm MG132/10 mm 3‐MA and 100 µm cycloheximide (CHX) at 30 °C for designated time points. Finally, the protein changes were detected via WB analysis using anti‐MBP antibodies.

### Drought Tolerance Assays and Stomatal Aperture Analysis

Drought tolerance assays were performed using 28‐day‐old WT and TOE3‐OE transgenic *N. tabacum* lines. Each experiment was independently repeated three times with similar results. Stomatal apertures in epidermal strips peeled from leaves of WT and TOE3‐OE transgenic *N. tabacum* plants, were photographed and measured using ImageJ software.

### Statistical Analysis

All experiments were repeated at least three times and statistically analyzed using Student's *t*‐tests or one‐way ANOVA with GraphPad Prism 8.0 software. The intensity of the protein bands in WB and the intensity of fluorescence were measured and analyzed using ImageJ software. The significance levels (^*^ *P* < 0.05, ^**^ *P* < 0.01, and ^***^ *P* < 0.001), and different lowercase letters (*P* < 0.05) were used to illustrate statistically significant differences.

## Conflict of Interest

D.X. and B.J. have filed a patent application in China (priority filing with serial number 202410898738.8).

## Author Contributions

The experiments are conceived and designed by D.X. and B.J. The experimental work and data analysis are carried out by B.J. and B.W. The manuscript is written by B.J. and subsequently revised by D.X. H.L. contributes by providing suggestions.

## Supporting information



Supporting Information

Supporting Information

## Data Availability

The data that support the findings of this study are available from the corresponding author upon reasonable request.
