# Supplementary material for: Post‐Translational Modifications of TOE3 Regulate Antiviral Defense in Tobacco
Source: Adv Sci (Weinh). 2025 Aug 14;12(42):e06243. doi: 10.1002/advs.202506243 (PMC12622502; doi:10.1002/advs.202506243)
Supplement: Supplementary file 1 — Supporting Information [file ADVS-12-e06243-s002.docx]

Supporting Information

Post-translational modifications of TOE3 regulate antiviral defense in tobacco

Bolei Jiao, Baijun Wu, Honghui Lin, and Dehui Xi*

**Supplementary Figures 1-17**

**Supplementary Tables 1-2**


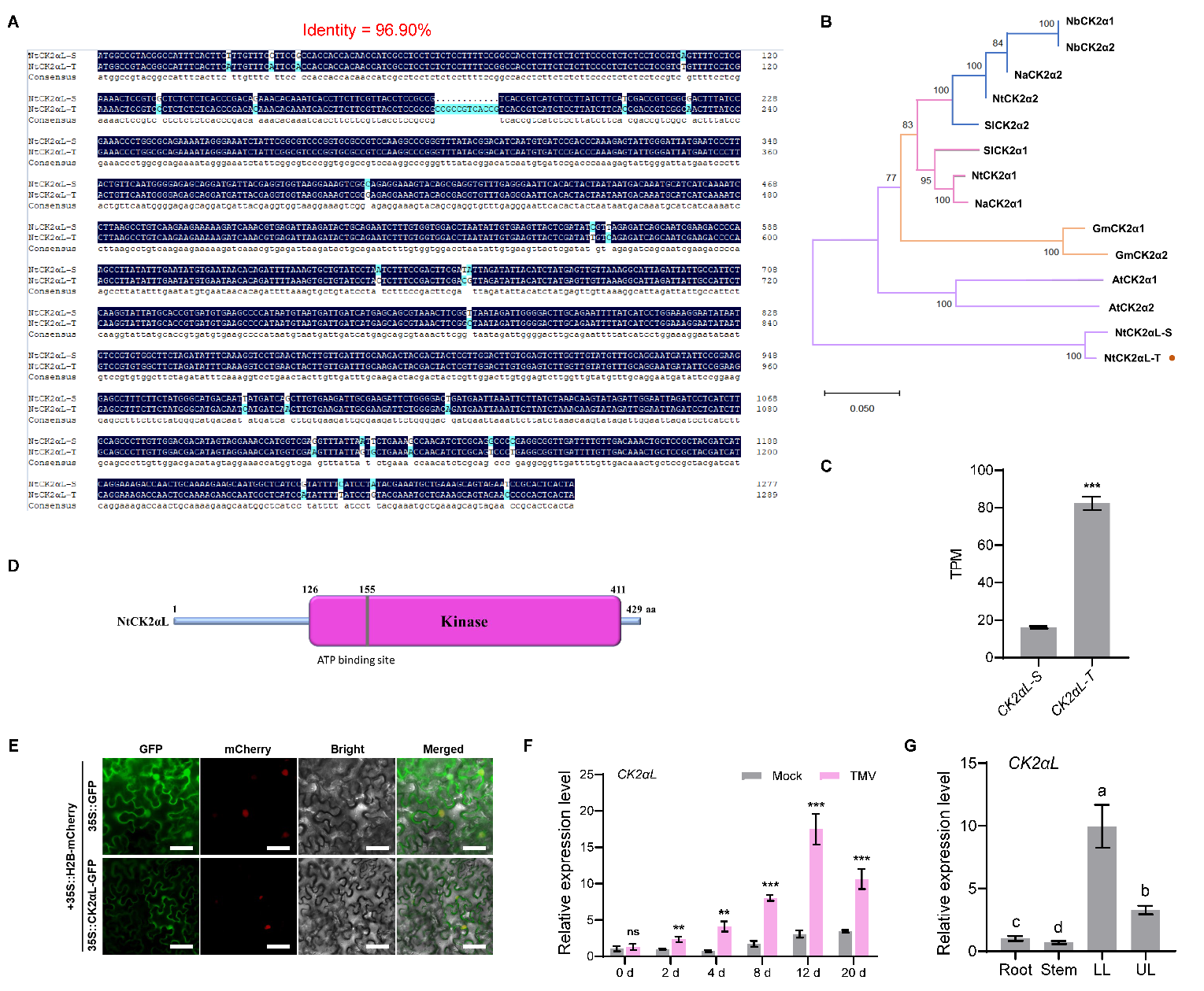
**Fig****ure S1.** The characteristics of NtCKαL. (A) Sequence alignment of *N**tCKαL-S* and *NtCKαL-T*, with conserved nucleic acids shaded in blue. (B) Phylogenetic analysis of CK2 subunits from *N. tabacum* and other plants using the neighbor-joining method in MEGA 11.0.13 with 1000 bootstrap iterations. The numbers at the nodes of the tree indicate bootstrap values from 1000 replicates. (C) The transcripts per million (TPM) of *CKαL-S* and *CKαL-T* in tobacco. Data are presented as means ± SD from three independent experiments. Significant differences are determined using Student’s t-test (****P* < 0.001). (D) The structure of NtCK2αL. The kinase domain is located at the C-terminal of CK2αL. (E) Subcellular localization of CKαL. Agrobacterium suspensions containing the control construct H2B-mCherry were co-infiltrated with the suspensions carrying GFP or CK2αL-GFP constructs, and served as a nuclear marker. Scale bars = 30 μm. (F) Temporal expression patterns of *CK2αL* in *N. tabacum* under TMV infection (0, 2, 4, 8, 12, and 20 dpi, respectively). Data are presented as means ± SD from three independent experiments. Significant differences are determined using Student’s t-test (***P* < 0.01, ****P* < 0.001). (G) Relative expression levels of *CK2αL* in *N. tabacum* leaves. LL: lower leaves; UL: upper leaves. Data are presented as means ± SD from three independent experiments. Statistical analysis was performed using one-way ANOVA with Tukey’s multiple-comparison test (different letters represent significantly different groups; *P* < 0.05).


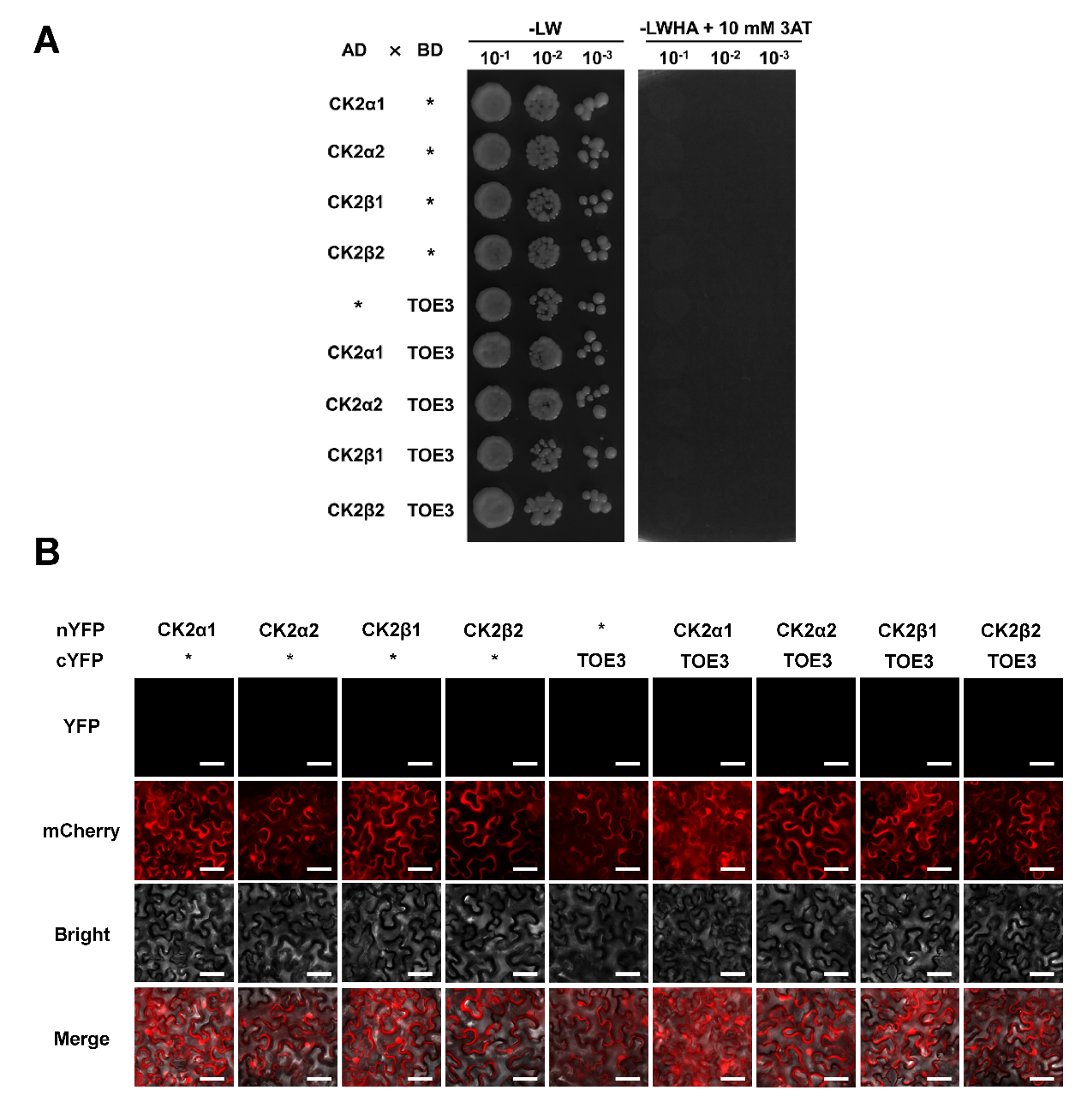


**Figure S2.** TOE3 does not interact with the subunits of CK2. (A) Y2H assay for the interaction between TOE3 and CK2 subunits. 10 mM 3-AT was used to inhibit self- activation activity. (B) BiFC assays of the interaction between TOE3 and CK2 subunits. Agrobacterium mixtures containing the control construct 35S: mCherry were co-infiltrated with the suspensions carrying BiFC constructs, and served as a marker for successful transfection. Scale bars represent 20 μm.


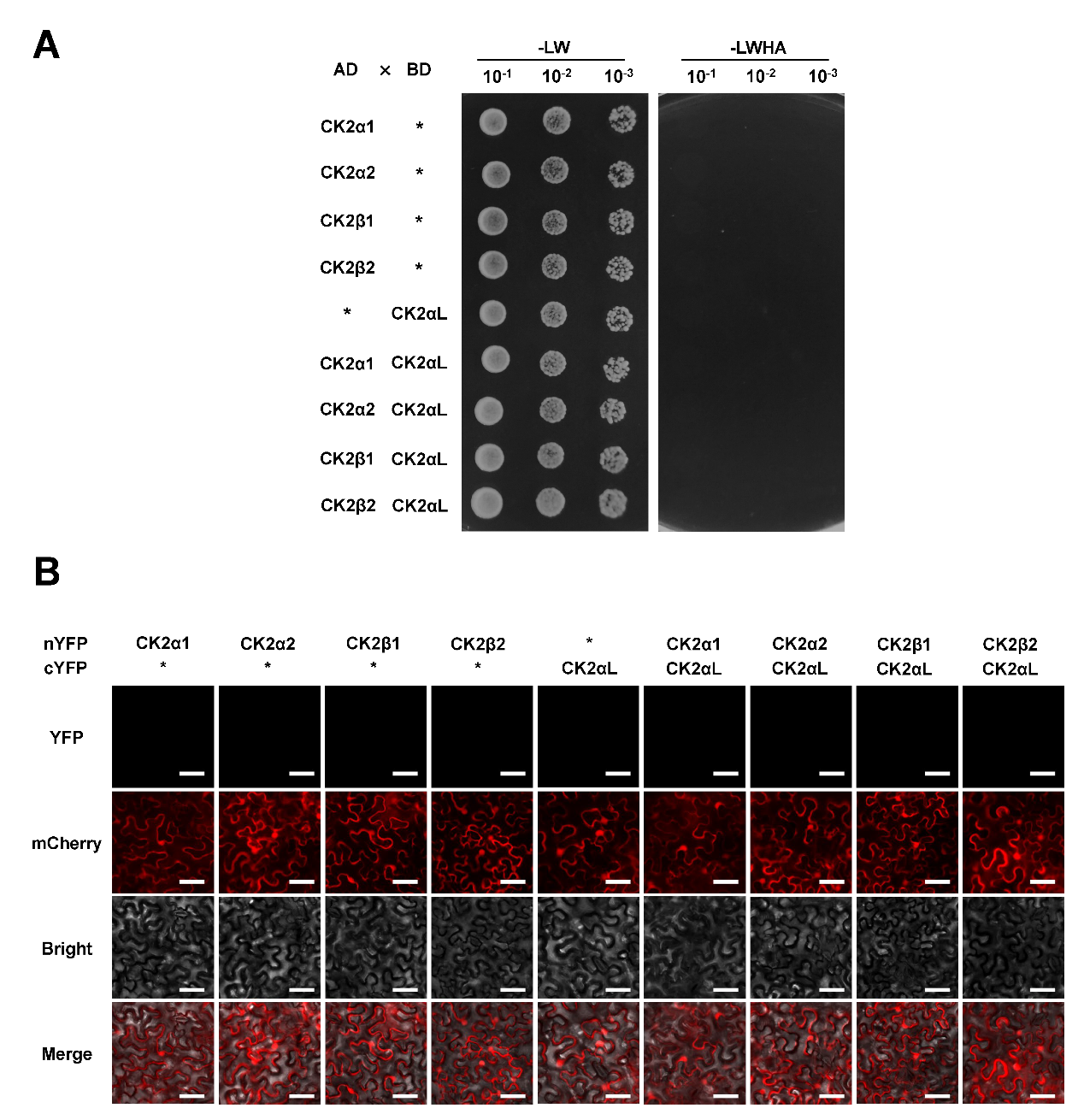


**Figure S3.** CK2αL does not physically interact with CK2 subunits. (A) Y2H assay of the interaction between CK2αL and other subunits of CK2. (B) BiFC assays of the interaction between CK2αL and CK2 subunits. Agrobacterium mixtures containing the control construct 35S: mCherry were co-infiltrated with the suspensions carrying BiFC constructs, and served as a marker for successful transfection. Scale bars represent 20 μm.


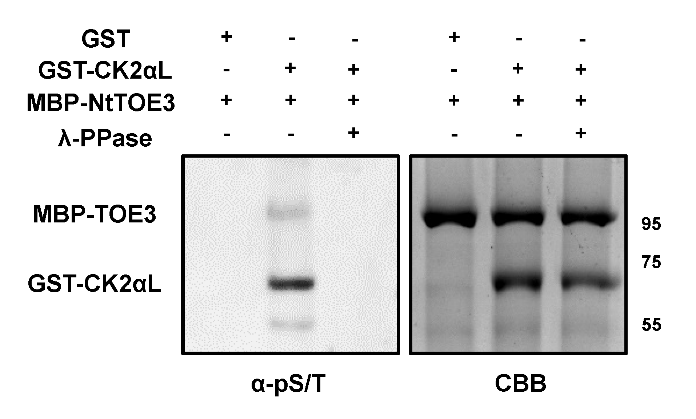


**Figure S4.** WB analysis of phosphorylated TOE3 by CK2αL *in vitro* using α-pS/T antibody. MBP-TOE3 was incubated with GST or GST-CK2αL at 30°C for 1 h. Proteins were separated in regular gels and then detected by WB analysis using anti-pS/T antibody. Meanwhile, another similar gel was subjected to CBB staining.


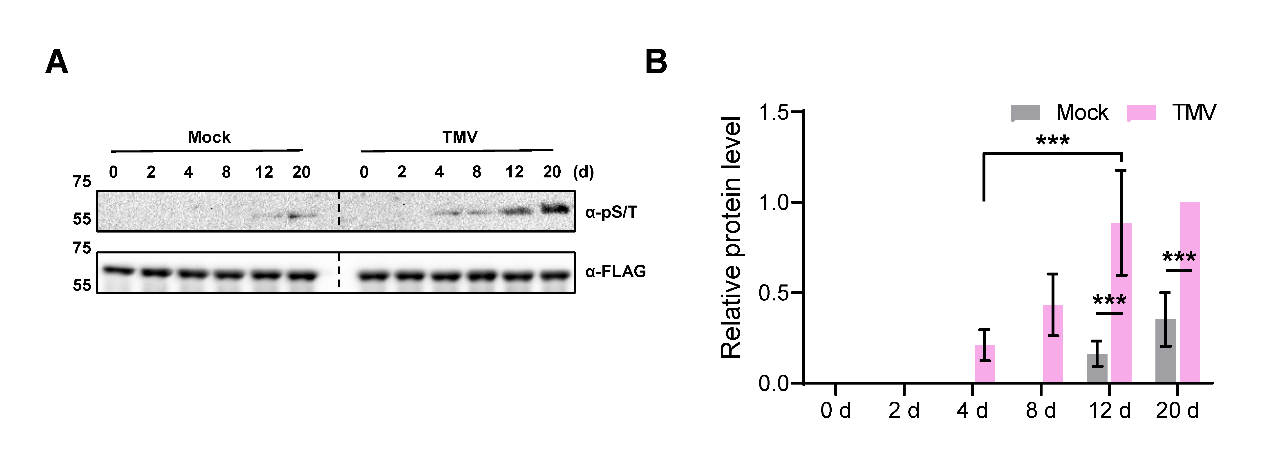


**Figure S5.** Time course analysis of the phosphorylation level of TOE3 during TMV infection. (A) WB analysis of TOE3-FLAG protein and phosphorylated TOE3-FLAG in TOE3-OE#4 plants after TMV infection. TOE3-OE#4 plants were inoculated with TMV. Proteins were extracted from Mock and TMV-infected plants at 0, 2, 4, 8, 12, and 20 dpi, respectively. TOE3-FLAG was immunoprecipitated with FLAG beads, then the immunoprecipitated proteins were detected using anti-FLAG and anti-pS/T antibodies, respectively. (B) The quantification analysis of phosphorylated TOE3-FLAG levels in (A). The relative phosphorylated TOE3-FLAG abundance was quantified and the protein level at 20 dpi were normalized to 1. Data are presented as means ± SD from three independent experiments. Statistical analysis was performed using one-way ANOVA with Tukey’s multiple-comparison test (****P* < 0.001).


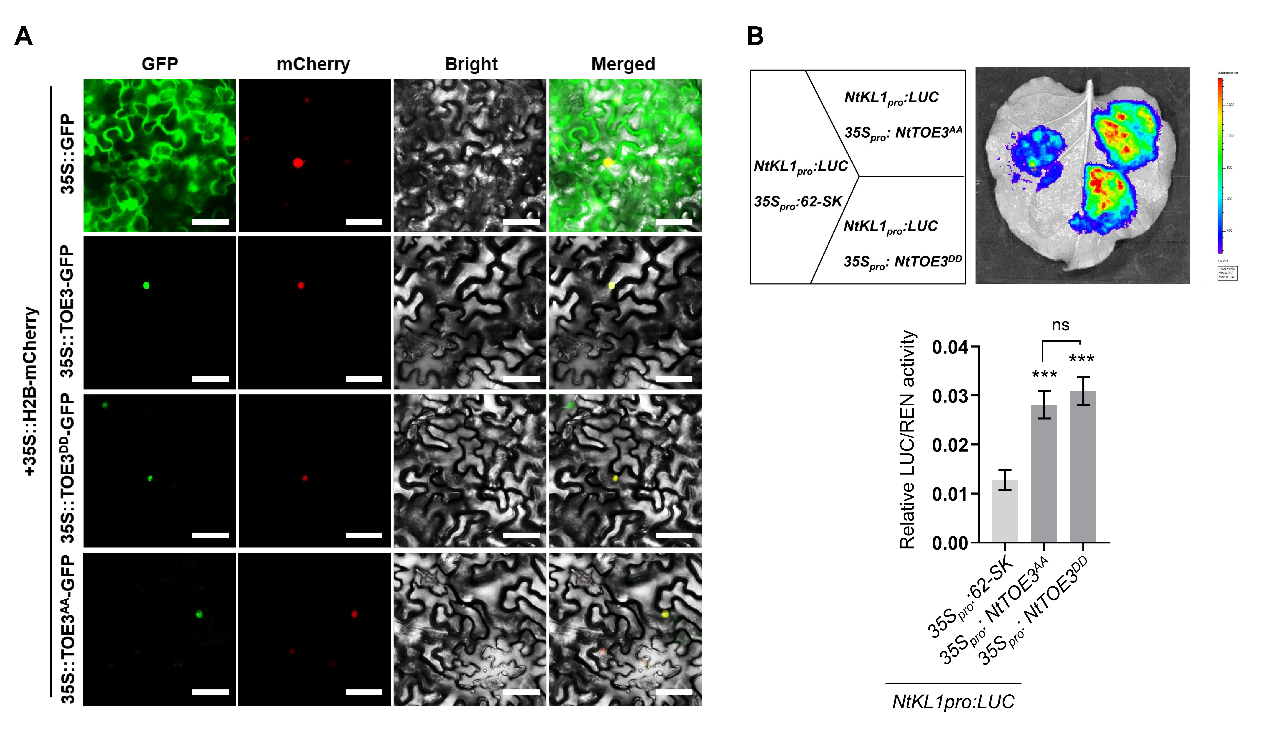


**Figure S6.** CK2αL-mediated phosphorylation does not affect the subcellular localization and transcriptional regulation activity of TOE3. (A) The subcellular localization of native TOE3, phosphorylated TOE3, and nonphosphorylated TOE3. Agrobacterium mixtures containing TOE3-GFP, TOE3^DD^-GFP, or TOE3^AA^-GFP and H2B-mCherry constructs were co-expressed in *N. benthamiana* leaves. The H2B-mCherry construct served as a nuclear marker. At 3 dpa, the subcellular localization of TOE3 was observed. Scale bars represent 50 μm. (B) Transcriptional activation assay illustrating no significant difference in the transcriptional activation of *KL1* by TOE3^DD^ and TOE3^AA^. Agrobacterium mixtures containing 35S_pro_: TOE3^DD^ or 35S_pro_: TOE3^AA^ and *KL1pro*: LUC were co-infiltrated into the leaves of *N. benthamiana*. At 3 dpa, the relative LUC/REN activities were detected. Data are presented as means ± SD from three independent experiments. Statistical analysis was performed using one-way ANOVA with Tukey’s multiple-comparison test (****P* < 0.001).


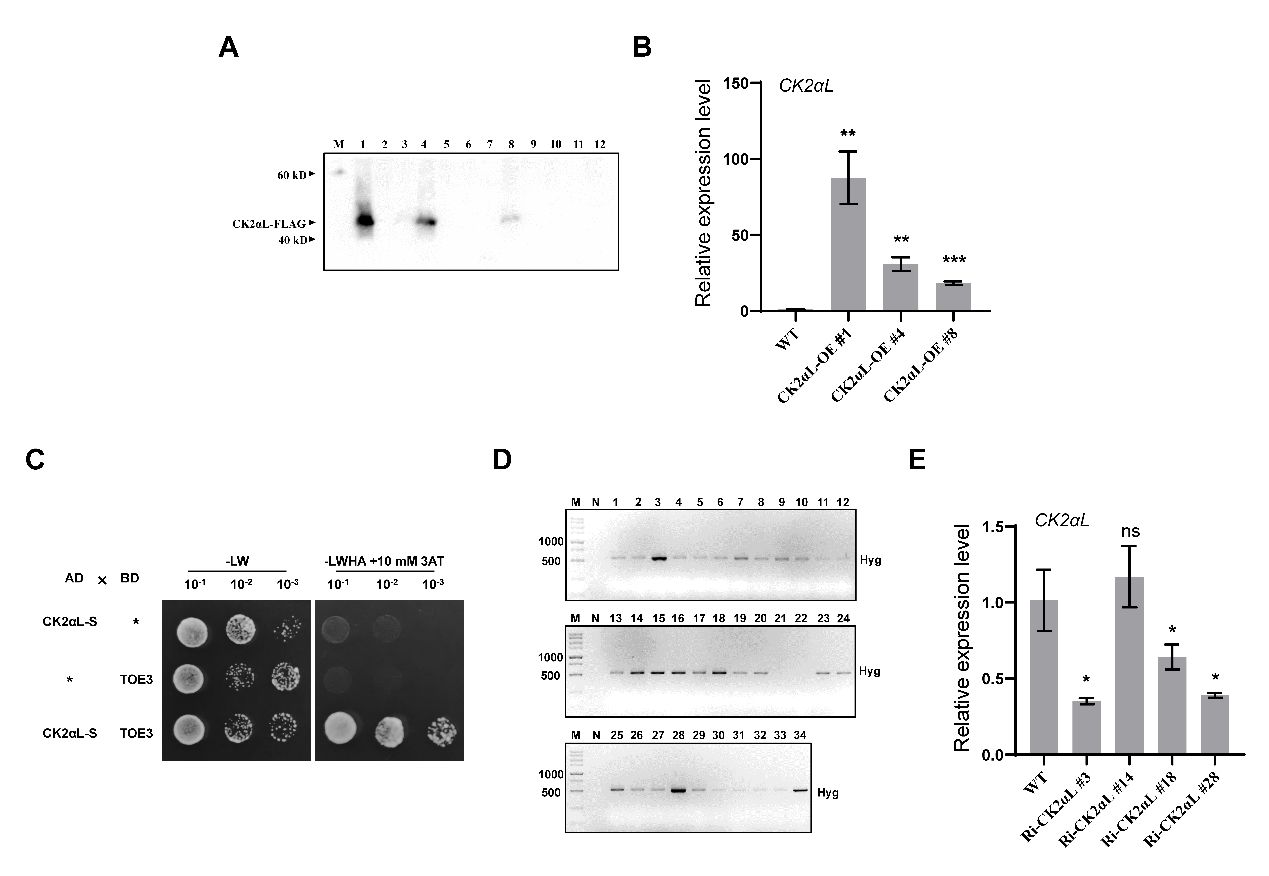
**Figure S7**. Identification of CK2αL-OE and Ri-CK2αL transgenic *N. tabacum* lines. (A) WB analysis of CK2αL-FLAG in CK2αL-OE lines and WT plants using anti-FLAG antibody. Lane M: EasySee western blotting marker. (B) The expression level of *CK2αL* in the selected CK2αL-OE lines and WT plants. Data are presented as means ± SD from three independent experiments. Significant differences are determined using Student’s t-test (***P* < 0.01, ****P* < 0.001). (C) Y2H assays of the interaction between TOE3 and CK2αL-S. 10 mM of 3-AT was used to inhibit self-activation activity. (D) RT-PCR was used to identify the resistance gene in Ri-CK2αL lines and WT plants. Lane M: DL5000 DNA marker; Lane N: WT as negative control; Hyg: Hygromycin B resistance gene. (E) The expression level of *CK2αL* in the selected Ri-CK2αL lines and WT plants. Data are presented as means ± SD from three independent experiments. Significant differences are determined using Student’s t-test (**P* < 0.05).


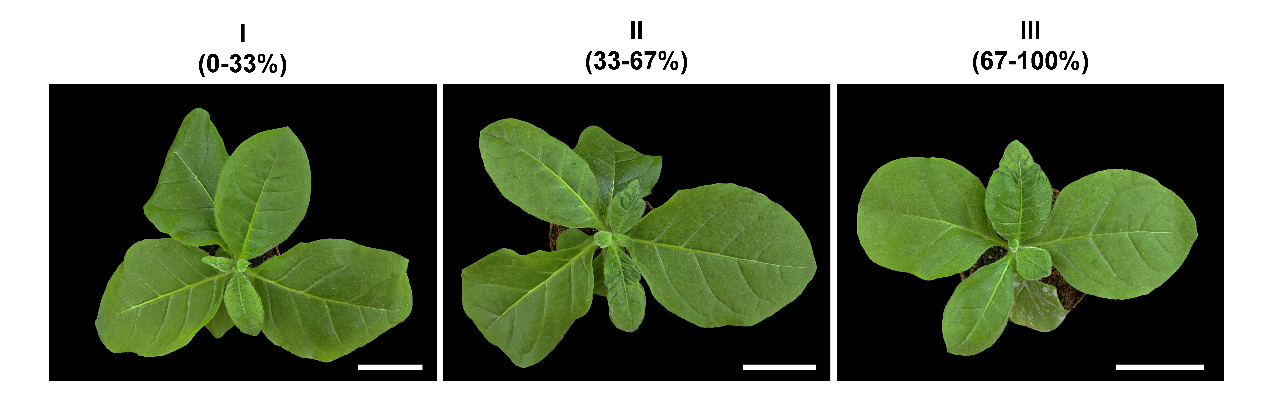


**Figure S8**. Photographs of TMV-infected WT tobacco plants with different degrees of disease symptoms. The photographs were taken at 35 dpi. Scale bars = 6 cm. I, 0-33% green recovery tissue on the second and third leaves at the top of the plant; II 33-67% green recovery tissue on the second and third leaves at the top of the plant; III 67-100% green recovery tissue on the second and third leaves at the top of the plant.


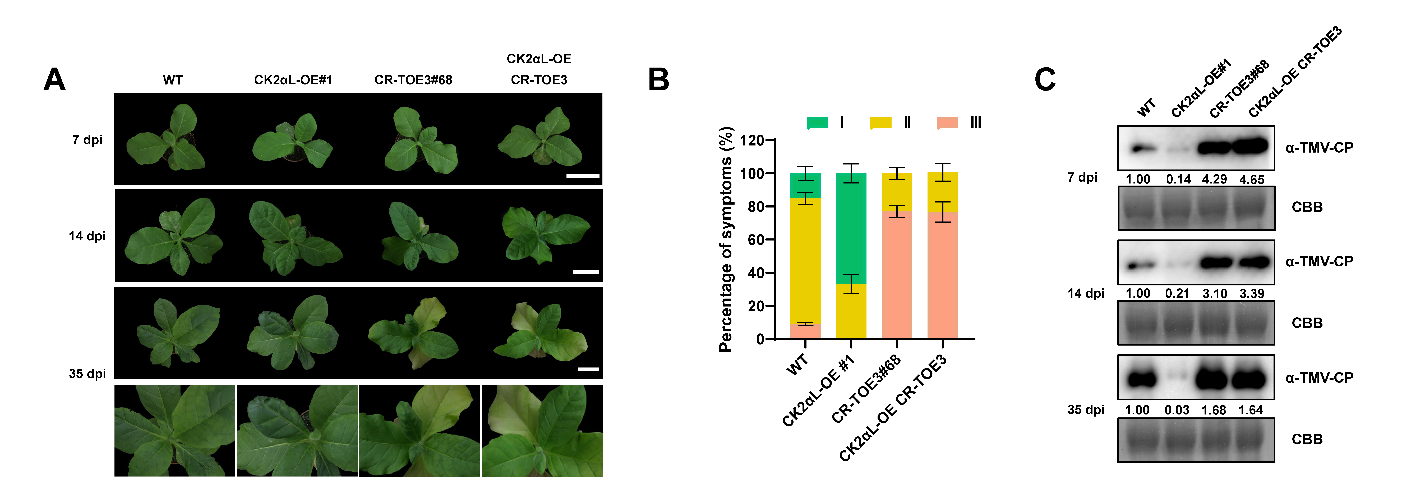
**Figure S9.** TOE3 acts downstream of CK2αL in tobacco resistance to TMV infection. (A) The disease symptoms of WT, CK2αL-OE#1, CR-TOE3#68, and CK2αL-OE CR-TOE3 tobacco plants at 7, 14, and 35 dpi. Scale bars = 5 cm. (B) The symptom percentages of TMV-infected WT, CK2αL-OE#1, CR-TOE3#68, and CK2αL-OE CR-TOE3 plants with different disease symptom grades at 35 dpi. Data are presented as means ± SD from three independent experiments. (C) WB analysis of viral accumulation of the indicated lines in (A) using anti-TMV-CP antibody.


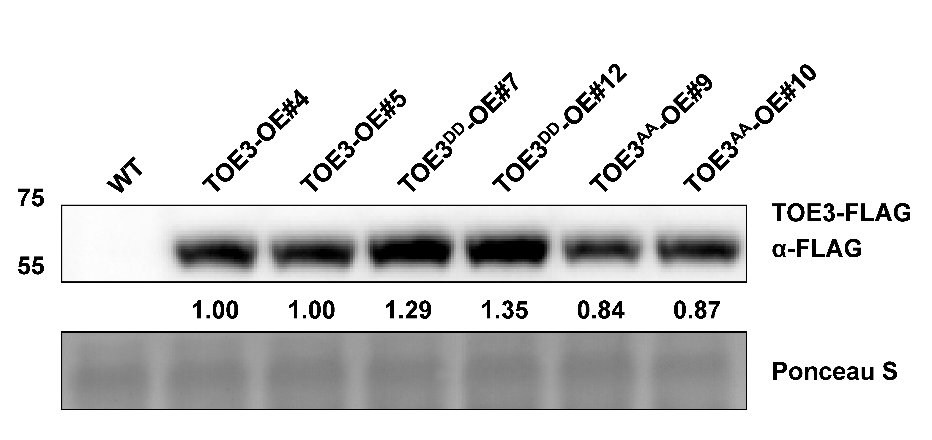


**Figure S10.** Identification of TOE3-OE, TOE3^DD^-OE, and TOE3^AA^-OE transgenic *N. tabacum* lines. WB analysis of TOE3-FLAG in WT, TOE3-OE, TOE3^DD^-OE, and TOE3^AA^-OE lines using anti-FLAG antibody.


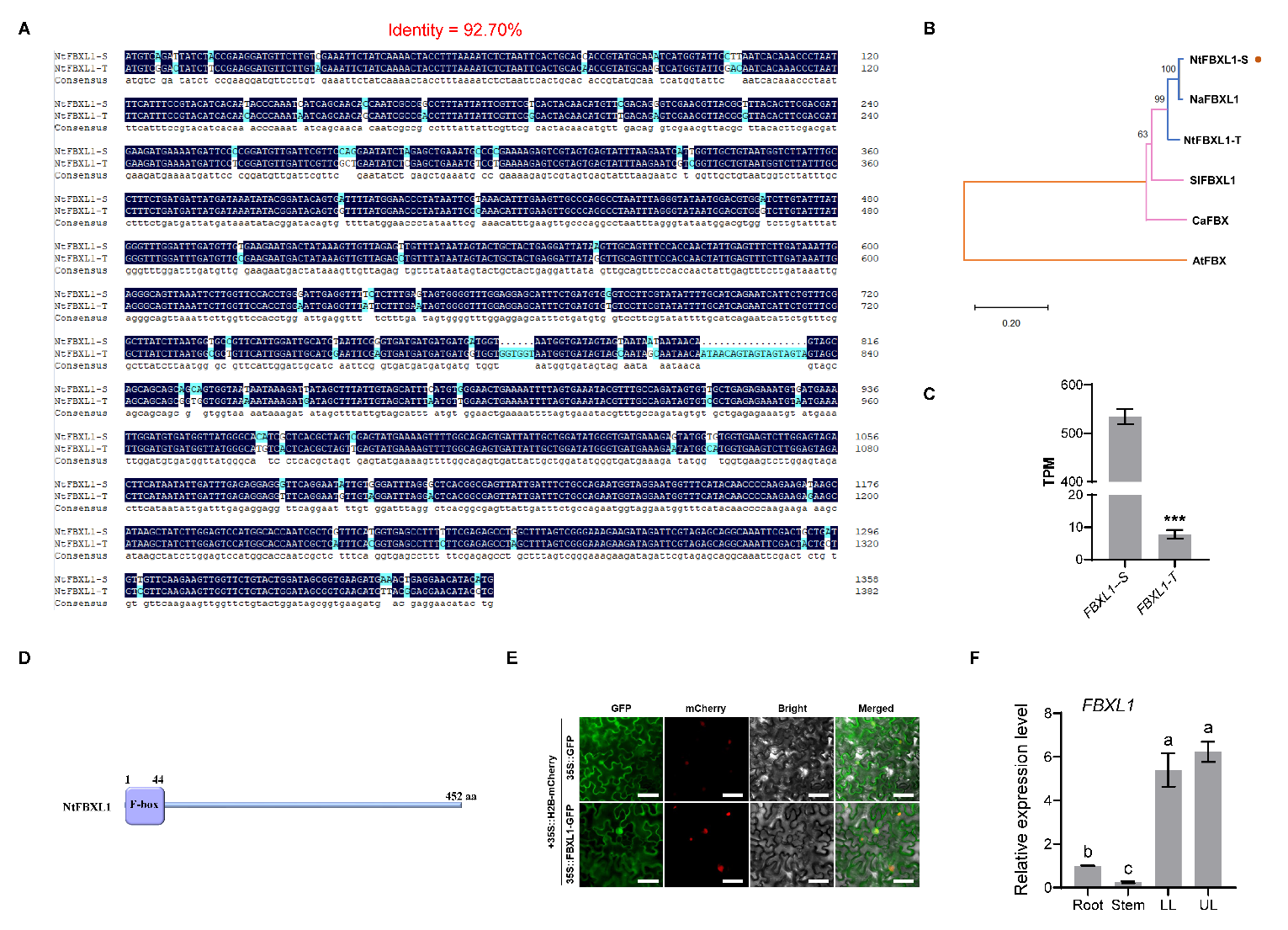
**Figure S11.** The characteristics of NtFBXL1. (A) Sequence alignment of *NtFBXL1-S* and *NtFBXL1-T*, with conserved nucleic acids shaded in blue. (B) Phylogenetic analysis of FBXL1 from *N. tabacum* and other plants using the neighbor-joining method in MEGA 11.0.13 with 1000 bootstrap iterations. The numbers at the nodes of the tree indicate bootstrap values from 1000 replicates. (C) The TPM of *FBXL1-S* and *FBXL1-T* in tobacco. Data are presented as means ± SD from three independent experiments. Significant differences are determined using Student’s t-test (****P* < 0.001). (D) The structure of NtFBXL1. The conserved F-box domain is located at the N-terminal of FBXL1. (E) Subcellular localization of FBXL1. The control construct H2B-mCherry was co-infiltrated with GFP or FBXL1-GFP constructs and served as a nuclear marker. Scale bars = 30 μm. (F) Relative expression levels of *FBXL1* in *N. tabacum* leaves. LL: lower leaves; UL: upper leaves. Data are presented as means ± SD from three independent experiments. Statistical analysis was performed using one-way ANOVA with Tukey’s multiple-comparison test (different letters represent significantly different groups; *P* < 0.05).


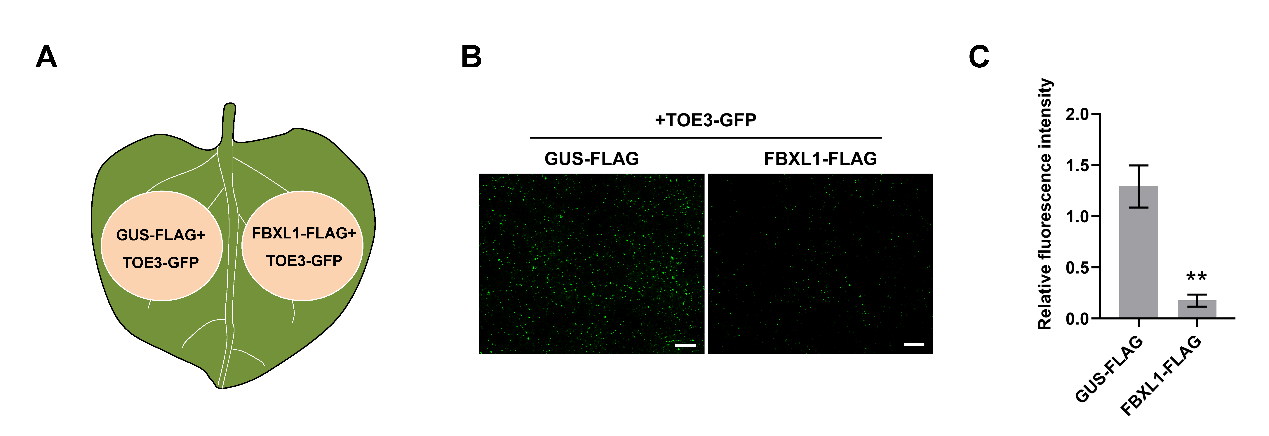


**Figure S12.** Overexpression of FBXL1 promotes the degradation of TOE3 *in planta*. (A) Schematic representation of combinations of protein co-expression in *N. benthamiana* leaves. Agrobacterium mixtures containing TOE3-GFP and GUS-FLAG or FBXL1-FLAG were co-infiltrated into *N. benthamiana* leaves. The fluorescence was observed at 3 dpa. (B) The observation of GFP fluorescence signal. The fluorescence signal of TOE3-GFP was lower in combination with FBXL1-FLAG. Scale bars = 250 μm. (C) Quantification of GFP fluorescence intensity in (*B*). Data are presented as means ± SD from three independent experiments. Significant differences are determined using Student’s t-test (***P* < 0.01).


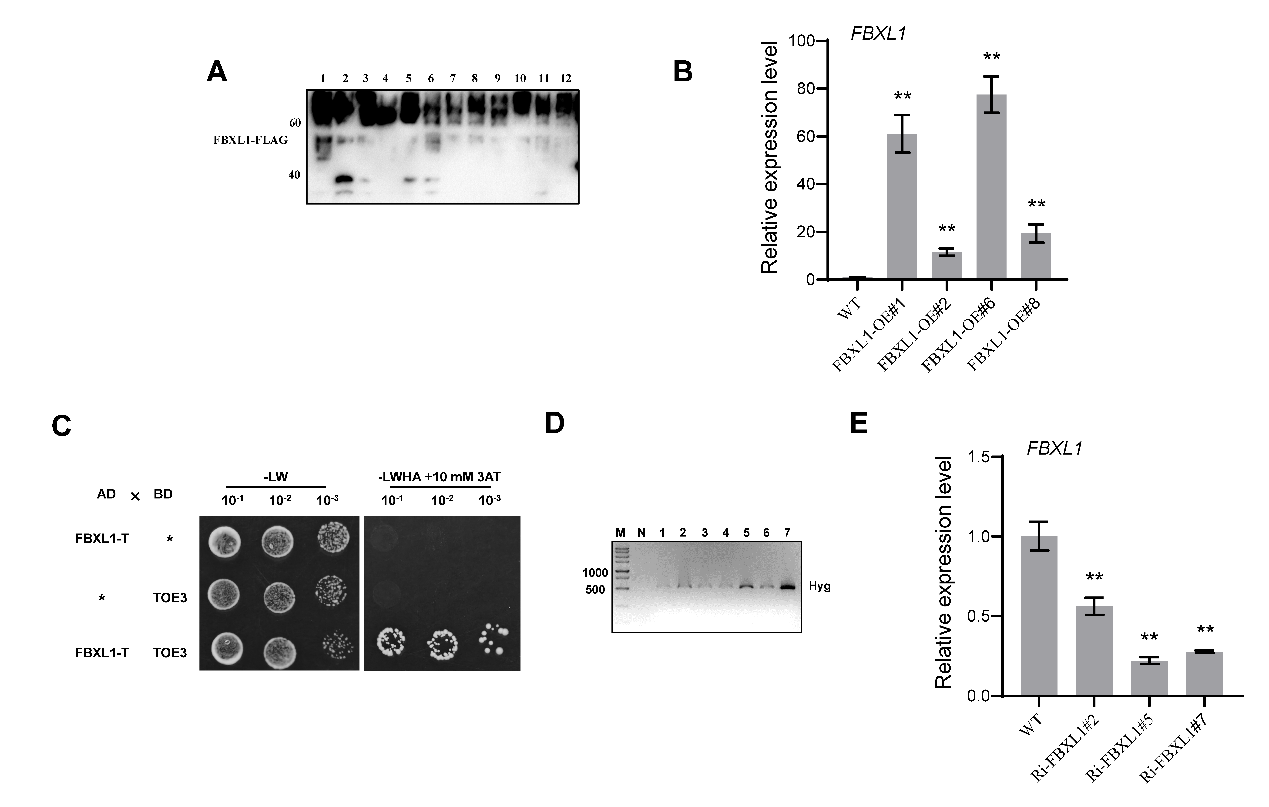


**Figure S13.** Identification of FBXL1-OE and Ri-FBXL1 transgenic *N. tabacum* lines. (A) WB analysis of FBXL1-FLAG in FBXL1-OE lines and WT plants using anti-FLAG antibody. (B) The expression level of *FBXL1* in the selected FBXL1-OE lines and WT plants. Data are presented as means ± SD from three independent experiments. Significant differences are determined using Student’s t-test (***P* < 0.01). (C) Y2H assays of the interaction between TOE3 and FBXL1-T. 10 mM of 3-AT was used to inhibit self-activation activity. (D) RT-PCR was used to identify the resistance gene in Ri-FBXL1 lines and WT plants. Lane M: DL5000 DNA marker; Lane N: WT as negative control; Hyg: Hygromycin B resistance gene. (E) The expression level of *FBXL1* in the selected Ri-FBXL1 lines and WT plants. Data are presented as means ± SD from three independent experiments. Significant differences are determined using Student’s t-test (***P* < 0.01).


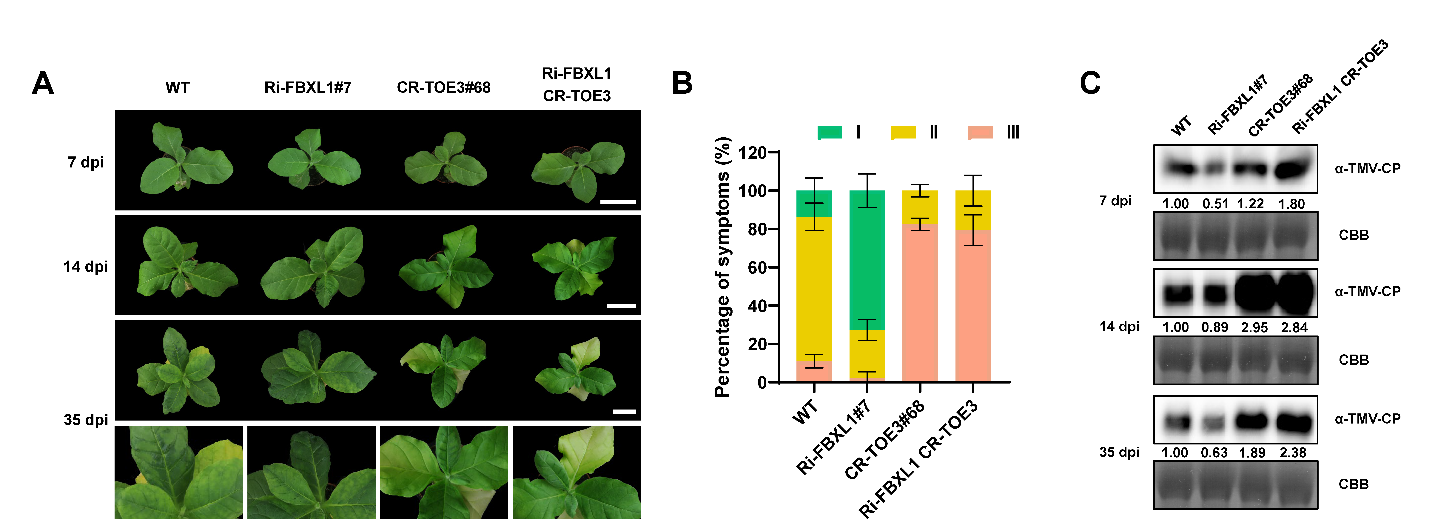
**Figure S14.** TOE3 acts downstream of FBXL1 in tobacco resistance to TMV infection. (A) The disease symptoms of WT, Ri-FBXL1#7, CR-TOE3#68, and Ri-FBXL1 CR-TOE3 tobacco plants at 7, 14, and 35 dpi. Scale bars = 5 cm. (B) The symptom percentages of TMV-infected WT, Ri-FBXL1#7, CR-TOE3#68, and Ri-FBXL1 CR-TOE3 plants with different disease symptom grades at 35 dpi. Data are presented as means ± SD from three independent experiments. (C) WB analysis of viral accumulation of the indicated lines in (A) using anti-TMV-CP antibody.


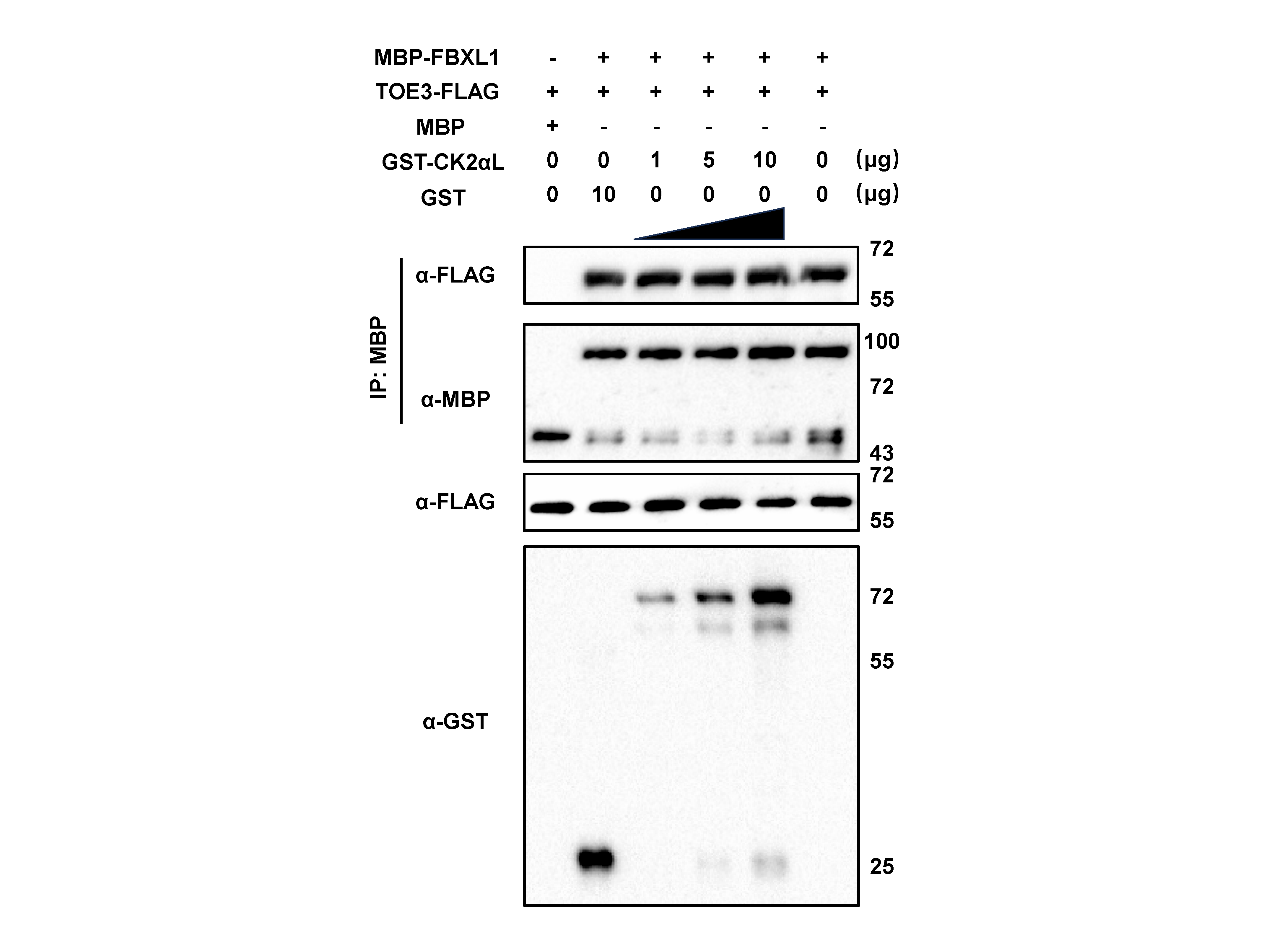


**Figure S15.** *In vitro* competitive pull-down assay of FBXL1 and CK2αL to TOE3. MBP-FBXL1 protein was used to pull down the TOE3-FLAG with increasing amounts of GST-CK2αL. MBP was used as a negative control. WB analysis was performed using anti-MBP, anti-FLAG, and anti-GST antibodies, respectively.


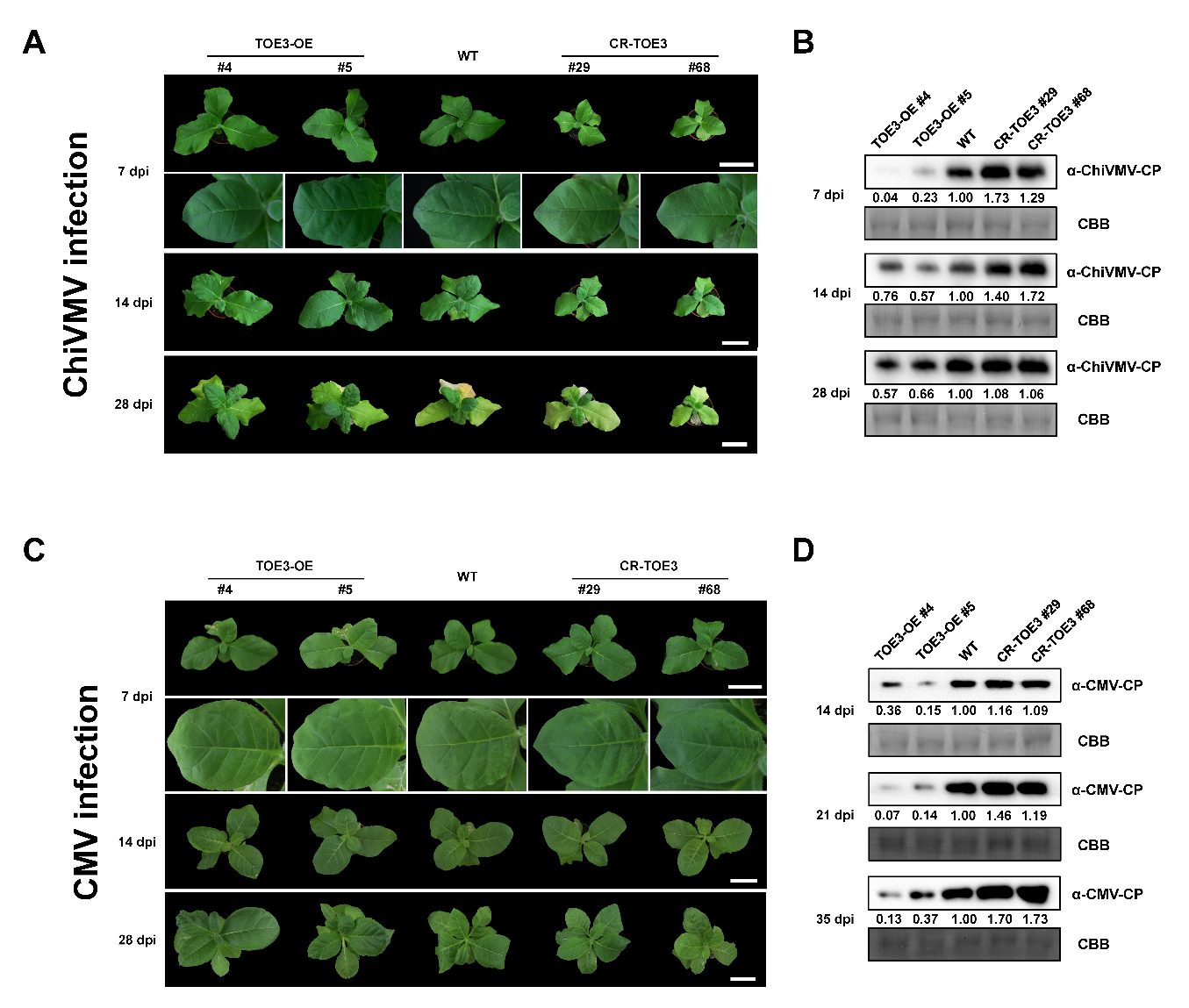
**Figure S16.** Overexpression of *TOE3* enhances the resistance of tobacco to ChiVMV and CMV infection. (A) The disease symptoms of ChiVMV-infected TOE3-OE, CR-TOE3, and WT tobacco plants at 7, 14, and 28 dpi. Scale bars = 5 cm. (B) WB analysis of ChiVMV-CP accumulation in the indicated lines in (A) using anti-ChiVMV-CP antibody. (C) The disease symptoms of CMV-infected TOE3-OE, CR-TOE3, and WT tobacco plants at 7, 14, and 28 dpi. Scale bars = 5 cm. (D) WB analysis of CMV-CP accumulation in the indicated lines in (C) using anti-CMV-CP antibody.


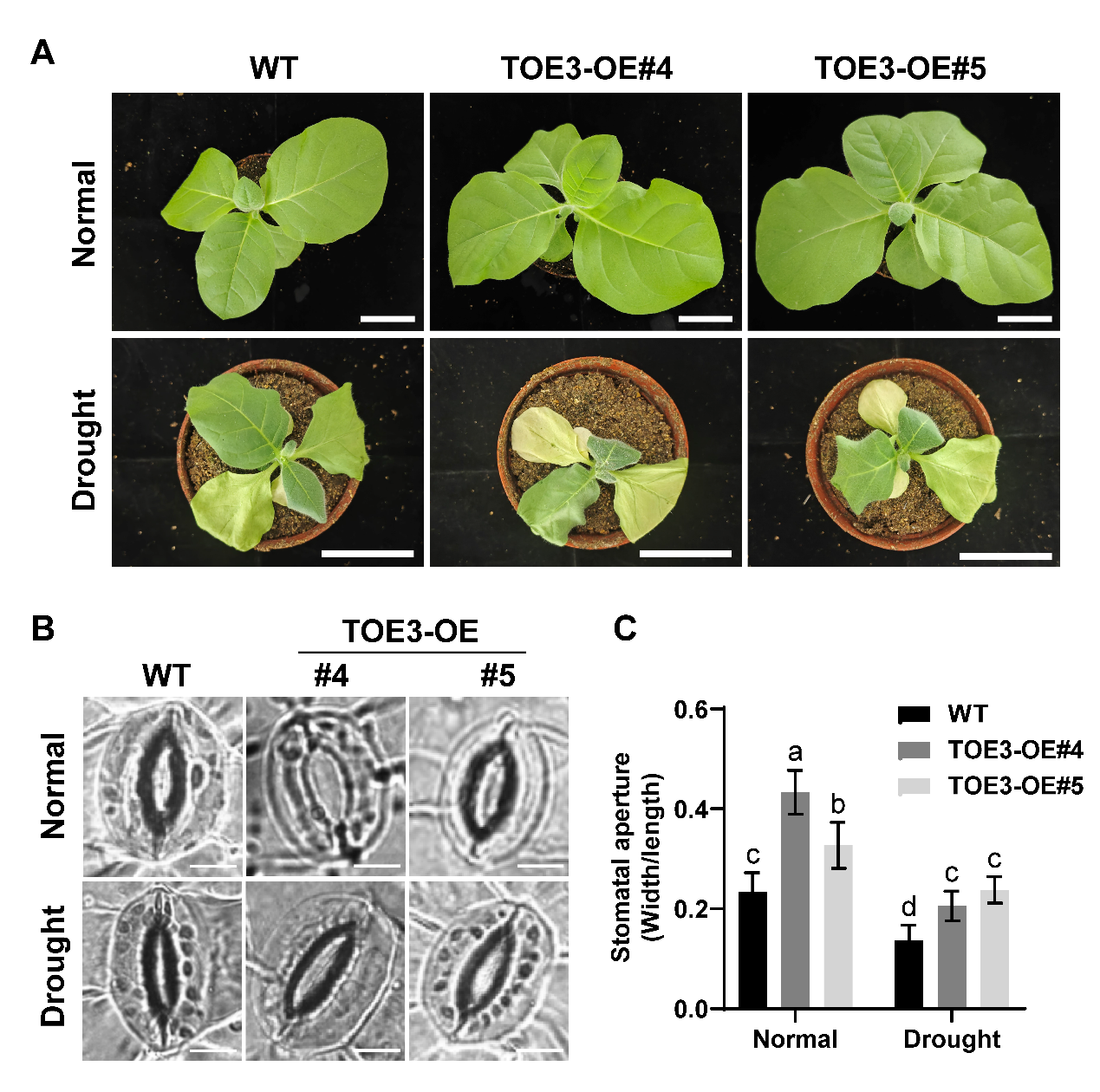


**Figure S17.** Overexpression of TOE3 decreases the drought tolerance of tobacco. (A) Phenotypes of WT, TOE3-OE#4, and TOE3-OE#5 seedlings under normal conditions (Normal) or subjected to drought stress. Scale bars = 5 cm. (B) Photographs of stomatal aperture in WT, TOE3-OE#4, and TOE3-OE#5 seedlings under normal conditions or drought stress. Scale bars = 10 μm. (C) Statistical analysis results of stomatal aperture in tobacco leaves of WT, TOE3-OE#4, and TOE3-OE#5 seedlings in (B). Data are presented as means ± SD from three independent experiments. Statistical analysis was performed using one-way ANOVA with Tukey’s multiple-comparison test (different letters represent significantly different groups; *P* < 0.05).

**Table S1.** The interacting proteins of TOE3 identified through Y2H screen.

| Number | NCBI reference sequence | Functional nnotation | Frequency of occurrence |
| --- | --- | --- | --- |
| 1 | XM_016589267.1 | CK2αL | 3 |
| 2 | XM_016599302.1 | FBXL1 | 2 |
| 3 | XM_016610315.1 | PYL4 | 3 |
| …… | …… | …… | …… |
| 25 | XM_016585394.1 | OEE1 | 1 |

**Table S2.** List of primers used in this study.

| **Primer** | **Sequence** | **Purpose** |
| --- | --- | --- |
| CK2αL-OE-F | ACGGGGGACAAGCTTGGTACCATGGCCGTACGGCCATTTCA | Construction for transgenic plants |
| CK2αL-OE-R | GTCTTTGTAGTCCATGTGAGTGCGGGTTCTAC | Construction for transgenic plants |
| FBXL1-OE-F | ACGGGGGACAAGCTTGGTACCATGTCAGATTATCTACCGAA | Construction for transgenic plants |
| FBXL1-OE-R | GTCTTTGTAGTCCATTGTATGTTCCTCAGTTTCAT | Construction for transgenic plants |
| Ri-CK2αL-1-F | ACGGGGGACAAGCTTGGTACCAACCATCGCCTCCTCTCTCC | Construction for transgenic plants |
| Ri-CK2αL-1-F | GCGGTATATACGTACGTCGACTCCCTATTTTCTGCGCCAGG | Construction for transgenic plants |
| Ri-CK2αL-2-F | GGCGTACGTGTGCAGGGATCCTCCCTATTTTCTGCGCCAGG | Construction for transgenic plants |
| Ri-CK2αL-2-F | AATTCGAGCTGGTCAGAGCTCAACCATCGCCTCCTCTCTCC | Construction for transgenic plants |
| Ri-FBXL1-1-F | ACGGGGGACAAGCTTGGTACCCTACCGAAGGATGTTCTTGT | Construction for transgenic plants |
| Ri-FBXL1-1-F | GCGGTATATACGTACGTCGACACCCTGTCGAACATGTTGTA | Construction for transgenic plants |
| Ri-FBXL1-2-F | GGCGTACGTGTGCAGGGATCCACCCTGTCGAACATGTTGTA | Construction for transgenic plants |
| Ri-FBXL1-2-F | AATTCGAGCTGGTCAGAGCTCCTACCGAAGGATGTTCTTGT | Construction for transgenic plants |
| Hyg-F | ACCGCGACGTCTGTCGAGAA | Identification for transgenic lines |
| Hyg-R | ACGAGGTGCCGGACTTCGGG | Identification for transgenic lines |
| CK2αL-GFP-F | CAGGGGCCCGGGGTCGACATGGCCGTACGGCCATTTCA | Subcellular location |
| CK2αL-GFP-R | CCTTGCTCACCATGGTACCGTGAGTGCGGGTTCTAC | Subcellular location |
| FBXL1-GFP-F | CAGGGGCCCGGGGTCGACATGTCAGATTATCTACCGAA | Subcellular location |
| FBXL1-GFP-R | CCTTGCTCACCATGGTACCTGTATGTTCCTCAGTTTCAT | Subcellular location |
| AD-CK2αL-F | GCCATGGAGGCCAGTGAATTCATGGCCGTACGGCCATTTCA | Y2H assay |
| AD-CK2αL-R | CAGCTCGAGCTCGATGGATCCTTAGTGAGTGCGGGTTCTAC | Y2H assay |
| BD-CK2αL-F | GGCCATGGAGGCCGAATTCATGGCCGTACGGCCATTTCA | Y2H assay |
| BD-CK2αL-R | CGCTGCAGGTCGACGGATCCTTAGTGAGTGCGGGTTCTAC | Y2H assay |
| AD-TOE3-F | GCCATGGAGGCCAGTGAATTCATGGAGTGTAGAGAAATGTG | Y2H assay |
| AD-TOE3-R | CAGCTCGAGCTCGATGGATCCGTCTATCTGCTGGGGGAATC | Y2H assay |
| BD-TOE3-F | GGCCATGGAGGCCGAATTCATGGAGTGTAGAGAAATGTG | Y2H assay |
| BD-TOE3-R | CGCTGCAGGTCGACGGATCCGTCTATCTGCTGGGGGAATC | Y2H assay |
| AD-CK2α1-F | GCCATGGAGGCCAGTGAATTCATGTCTAAAGCTCGAGTATA | Y2H assay |
| AD-CK2α1-R | CAGCTCGAGCTCGATGGATCCCTGTGTCCTCATCCTGCTAG | Y2H assay |
| AD-CK2α2-F | GCCATGGAGGCCAGTGAATTCATGTCGAAAGCTCGTGTTTA | Y2H assay |
| AD-CK2α2-R | CAGCTCGAGCTCGATGGATCCCTGCGTCCTCATCCTGCTAT | Y2H assay |
| AD-CK2β1-F | GCCATGGAGGCCAGTGAATTCATGTACAAAGAAAGGAGAGG | Y2H assay |
| AD-CK2β1-R | CAGCTCGAGCTCGATGGATCCCGGCTTGTGGACCTTGAAAC | Y2H assay |
| AD-CK2β2-F | GCCATGGAGGCCAGTGAATTCATGTATAGAGATCGAGGAGG | Y2H assay |
| AD-CK2β2-R | CAGCTCGAGCTCGATGGATCCAGTCTTGTGGATCTTGAAAC | Y2H assay |
| BD-FBXL1-F | GGCCATGGAGGCCGAATTCATGTCAGATTATCTACCGAA | Y2H assay |
| BD-FBXL1-R | CGCTGCAGGTCGACGGATCCTGTATGTTCCTCAGTTTCAT | Y2H assay |
| AD-FBXL1-F | GCCATGGAGGCCAGTGAATTCATGTCAGATTATCTACCGAA | Y2H assay |
| AD-FBXL1-R | CAGCTCGAGCTCGATGGATCCTGTATGTTCCTCAGTTTCAT | Y2H assay |
| NY-CK2αL-F | ATTACAGGTACCCGGGGATCCATGGCCGTACGGCCATTTCA | BiFC assay |
| NY-CK2αL-R | GCTCACCATACCGCCGTCGACGTGAGTGCGGGTTCTAC | BiFC assay |
| CY-CK2αL-F | ATTACAGGTACCCGGGGATCCATGGCCGTACGGCCATTTCA | BiFC assay |
| CY-CK2αL-R | CACGCTGCCACCGCCGTCGACGTGAGTGCGGGTTCTAC | BiFC assay |
| NY-TOE3-F | ATTACAGGTACCCGGGGATCCATGGAGTGTAGAGAAATGTG | BiFC assay |
| NY-TOE3-R | GCTCACCATACCGCCGTCGACGTCTATCTGCTGGGGGAATC | BiFC assay |

Continued Table S2

| **Primer** | **Sequence** | **Purpose** |
| --- | --- | --- |
| CY-TOE3-F | ATTACAGGTACCCGGGGATCCATGGAGTGTAGAGAAATGTG | BiFC assay |
| CY-TOE3-R | CACGCTGCCACCGCCGTCGACGTCTATCTGCTGGGGGAATC | BiFC assay |
| NY-CK2α1-F | ATTACAGGTACCCGGGGATCCATGTCTAAAGCTCGAGTATA | BiFC assay |
| NY-CK2α1-R | GCTCACCATACCGCCGTCGACCTGTGTCCTCATCCTGCTAG | BiFC assay |
| NY-CK2α2-F | ATTACAGGTACCCGGGGATCCATGTCGAAAGCTCGTGTTTA | BiFC assay |
| NY-CK2α2-R | GCTCACCATACCGCCGTCGACCTGCGTCCTCATCCTGCTAT | BiFC assay |
| NY-CK2β1-F | ATTACAGGTACCCGGGGATCCATGTACAAAGAAAGGAGAGG | BiFC assay |
| NY-CK2β1-R | GCTCACCATACCGCCGTCGACCGGCTTGTGGACCTTGAAAC | BiFC assay |
| NY-CK2β2-F | ATTACAGGTACCCGGGGATCCATGTATAGAGATCGAGGAGG | BiFC assay |
| NY-CK2β2-R | GCTCACCATACCGCCGTCGACAGTCTTGTGGATCTTGAAAC | BiFC assay |
| GST-CK2αL-F | GATCTGGTTCCGCGTGGATCCATGGCCGTACGGCCATTTCA | Pull-down assay |
| GST-CK2αL-R | GATGCGGCCGCTCGAGTCGACTTAGTGAGTGCGGGTTCTAC | Pull-down assay |
| GST-FBXL1-F | GATCTGGTTCCGCGTGGATCCATGTCAGATTATCTACCGAA | Pull-down assay |
| GST-FBXL1-R | GATGCGGCCGCTCGAGTCGACTGTATGTTCCTCAGTTTCAT | Pull-down assay |
| MBP-TOE3-F | GTACCACGTGGAAGCCATATGGAGTGTAGAGAAATGTG | Pull-down assay |
| MBP-TOE3-R | TGCGGCCGCAAGCTTGTCGACGTCTATCTGCTGGGGGAATC | Pull-down assay |
| His-TOE3-F | CAGCAAATGGGTCGCGGATCCATGGAGTGTAGAGAAATGTG | Pull-down assay |
| His-TOE3-R | TGCGGCCGCAAGCTTGTCGACCTAGTCTATCTGCTGGGGGA | Pull-down assay |
| syn-TOE3(DD) | ATGGAGTGTAGAGAAATGTGGGATCTAAACGATTCTCCAGATCGACGAAGGGATGAAAAATCAGAAGAAGGCTGCTCTTCTCCTATAGAGCTAGAGGGCGATGATGAGAAAGGTAAACGGGTCGGATCCGTTTCGAATTCAAGTTCATCGGCAGTAGCTATTGATGATATTGACGAGGAGGAAGATGGAGAAAAAGGCAAGAAAAAGAGAAGTAGTCCTAGCAAAATATTCGGCTTCTCCGTGGTGGGTCCCGGTAACGACGATGAGGAACAGCCGGTAACCCGTCAGTTTTTTCCGGTTGATGAGTCTGAAACGAGTGCGCCTACCAATGGATCCCCGAATTTTCCCATGGCTCACTGGGTTGGAGTTAAATTTTACCAAGACGAGCCACTTGGCAAC | synthesis for site-mutated TOE3 |
| syn-TOE3(AA) | ATGGAGTGTAGAGAAATGTGGGATCTAAACGATTCTCCAGATCGACGAAGGGATGAAAAATCAGAAGAAGGCTGCTCTTCTCCTATAGAGCTAGAGGGCGATGATGAGAAAGGTAAACGGGTCGGATCCGTTTCGAATTCAAGTTCATCGGCAGTAGCTATTGATGATATTGCCGAGGAGGAAGATGGAGAAAAAGGCAAGAAAAAGAGAAGTAGTCCTAGCAAAATATTCGGCTTCTCCGTGGTGGGTCCCGGTAACGACGATGAGGAACAGCCGGTAACCCGTCAGTTTTTTCCGGTTGATGAGTCTGAAACGAGTGCGCCTACCAATGGATCCCCGAATTTTCCCATGGCTCACTGGGTTGGAGTTAAATTTTACCAAGCCGAGCCACTTGGCAAC | synthesis for site-mutated TOE3 |
| KL1-LUC-F | GTCGACGGTATCGATAAGCTTCTTATAAATCACGTATCTAT | Lucifease assay |
| KL1-LUC-R | AGGAAGGGTCTTGCGGGATCCATTTTCTTCTTCAATATAGTAG | Lucifease assay |

Continued Table S2

| **Primer** | **Sequence** | **Purpose** |
| --- | --- | --- |
| TOE3-OE-F | ACGGGGGACAAGCTTGGTACCATGGAGTGTAGAGAAATGTG | Gene-overexpression |
| TOE3-OE-R | GTCTTTGTAGTCCATGTCTATCTGCTGGGGGAATC | Gene-overexpression |
| NL-FBXL1-F | AACACGGGGGACGAGCTCGGTACCATGGCCGTACGGCCATTTCA | Split-LUC complementation assay |
| NL-FBXL1-F | ACGCGTACGAGATCTGGTCGACTTAGTGAGTGCGGGTTCTAC | Split-LUC complementation assay |
| CL-TOE3-F | TACGCGTCCCGGGGCGGTACCATGGAGTGTAGAGAAATGTG | Split-LUC complementation assay |
| CL-TOE3-R | ACGAAAGCTCTGCAGGTCGACGTCTATCTGCTGGGGGAATC | Split-LUC complementation assay |
| His-SKP1-F | CAGCAAATGGGTCGCGGATCCATGTCTACTTCAAAAATGAT | Ubiquitination assay |
| His-SKP1-R | TGCGGCCGCAAGCTTGTCGACCTCAAAGGCCCAGGCATTCT | Ubiquitination assay |
| His-CULLIN1-F | CAGCAAATGGGTCGCGGATCCATGAATCAGCGTACCACAAT | Ubiquitination assay |
| His-CULLIN1-R | TGCGGCCGCAAGCTTGTCGACTGCCAAGTATTTGAACAAGT | Ubiquitination assay |
| His-RBX1-F | CAGCAAATGGGTCGCGGATCCATGGCATCTGTTGACACCGA | Ubiquitination assay |
| His-RBX1-R | TGCGGCCGCAAGCTTGTCGACGTGACCATATTTCTGAAACT | Ubiquitination assay |
| qEF1α(Actin)-F | ACGCACTGCTTGCTTTCA | qPCR |
| qEF1α(Actin)-R | AACCTCCTTCACGATTTCAT | qPCR |
| qTMV-CP-F | GGACAGGAGGATTACAATAGG | qPCR |
| qTMV-CP-R | GGACACCAGGAGCATAGT | qPCR |
| qCK2αL-F | ACCTTCTTCGTTACCTCCGC | qPCR |
| qCK2αL-R | ACCTCGTAATCATCCTGCTC | qPCR |
| qFBXL1-F | ATCGCCGGCCTTTATTATTC | qPCR |
| qFBXL1-R | ATCAGAAAGGCAAATAAGACC | qPCR |
| qKL1-F | AGCCGTAGATCATGAGAAGA | qPCR |
| qKL1-R | ATCTTTGGTCACATGTAGGAC | qPCR |
| qMLP43-F | AGGCAAGTTGATCTCTCAAA | qPCR |
| qMLP43-R | ACTTCCAGCCGACAACAGAA | qPCR |
| qOsmotin-F | ATGGGCAACTTGAGATCTTC | qPCR |
| qOsmotin-R | ATCACCCAAGTTTGGCCTCG | qPCR |
| qPR4A-F | ATAAGTTGTGCGTGGCATTG | qPCR |
| qPR4A-R | ATTTCTGGCGCCATGCGAGA | qPCR |
