# Supplementary material for: Post‐Translational Modifications of TOE3 Regulate Antiviral Defense in Tobacco
Source: Adv Sci (Weinh). 2025 Aug 14;12(42):e06243. doi: 10.1002/advs.202506243 (PMC12622502; doi:10.1002/advs.202506243)

Fig. 1B

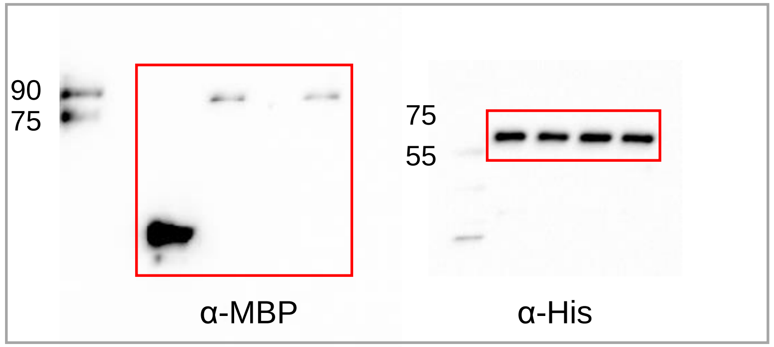

Fig. 1D

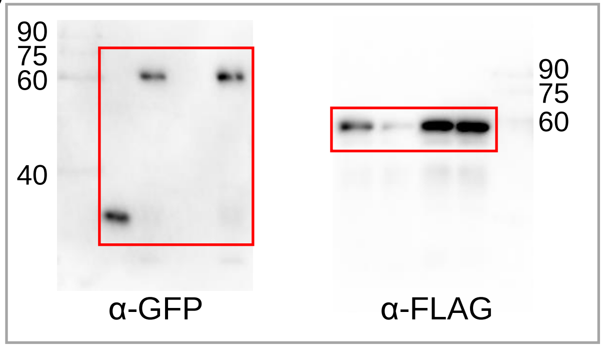

Fig. 2A

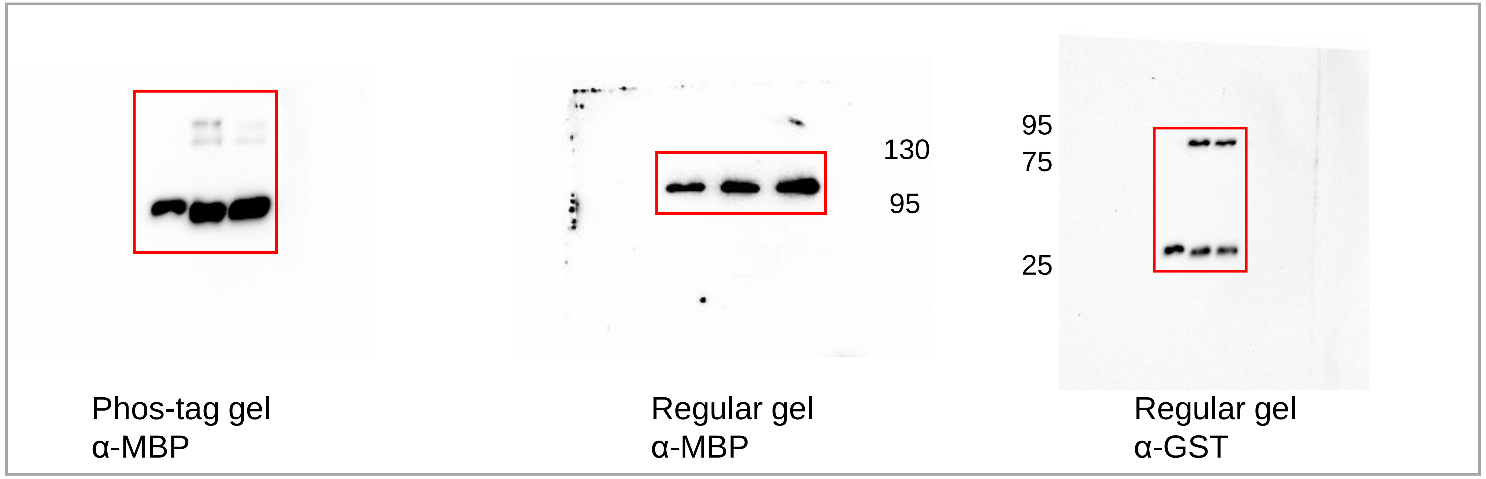

Fig. 2C

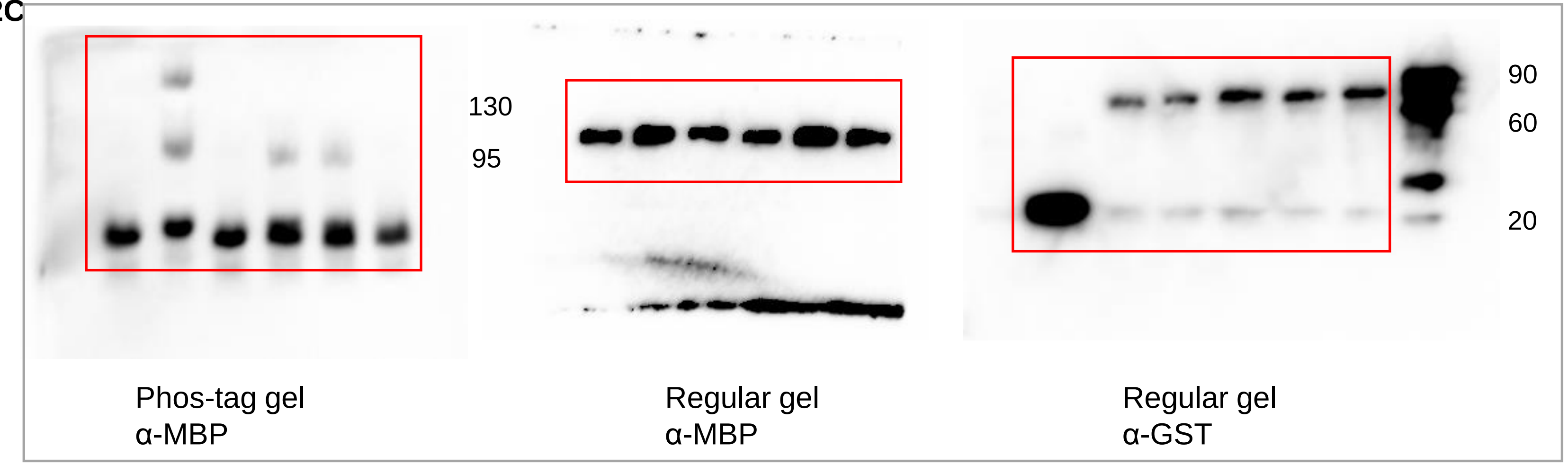

Fig. 2E

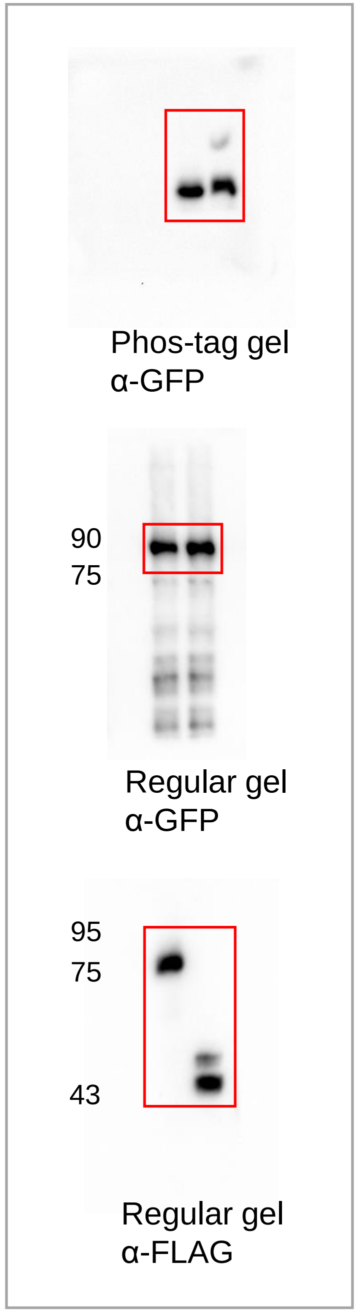

Fig. 3A

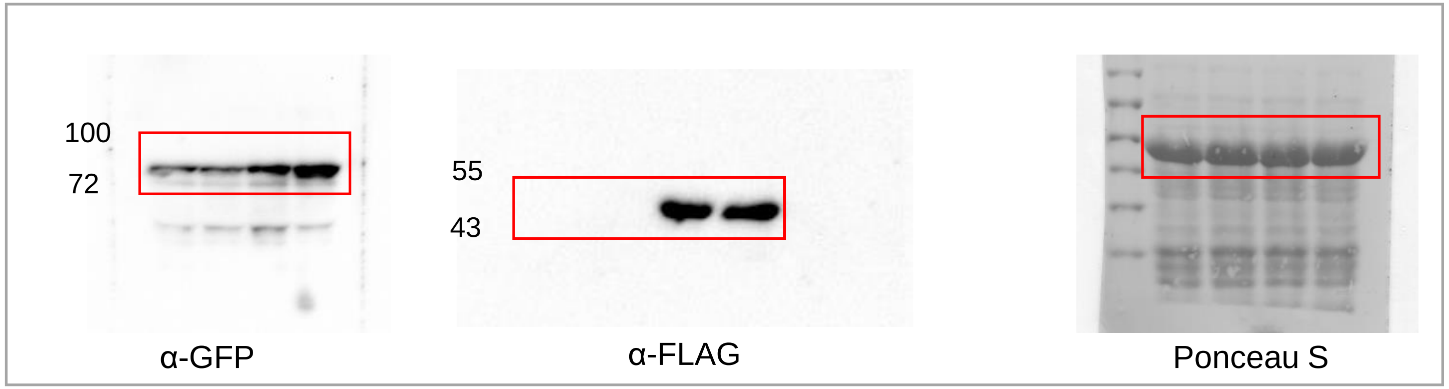

Fig. 3B

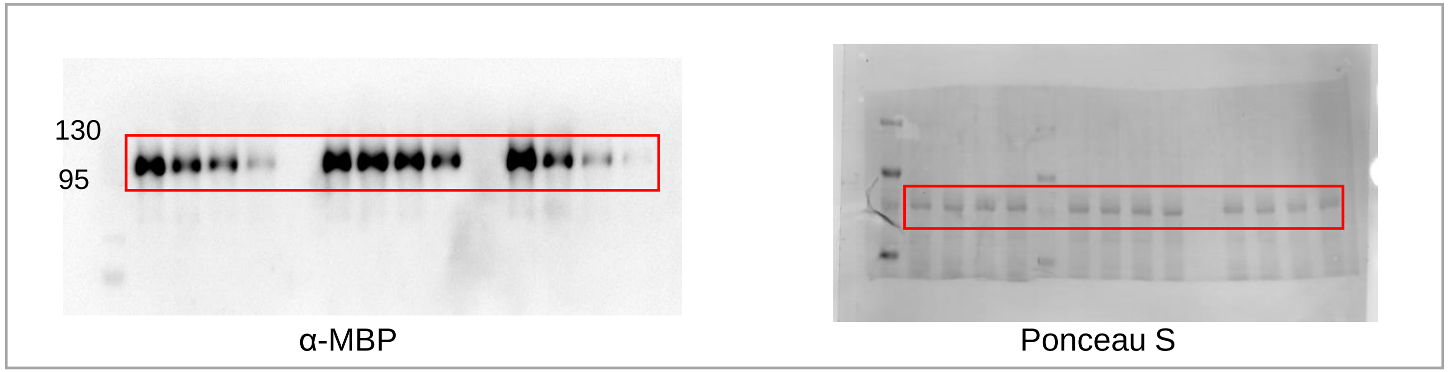

Fig. 3E

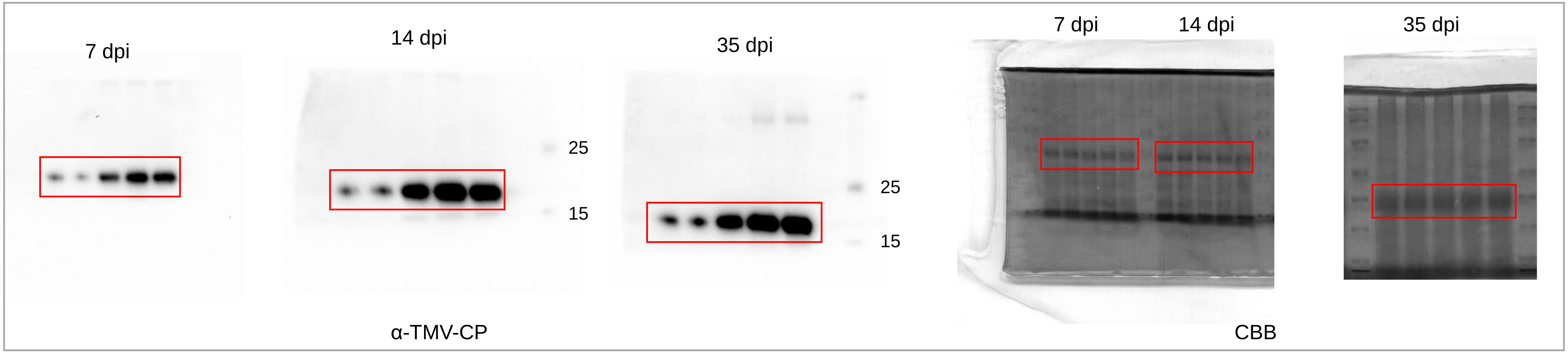

Fig. 4C

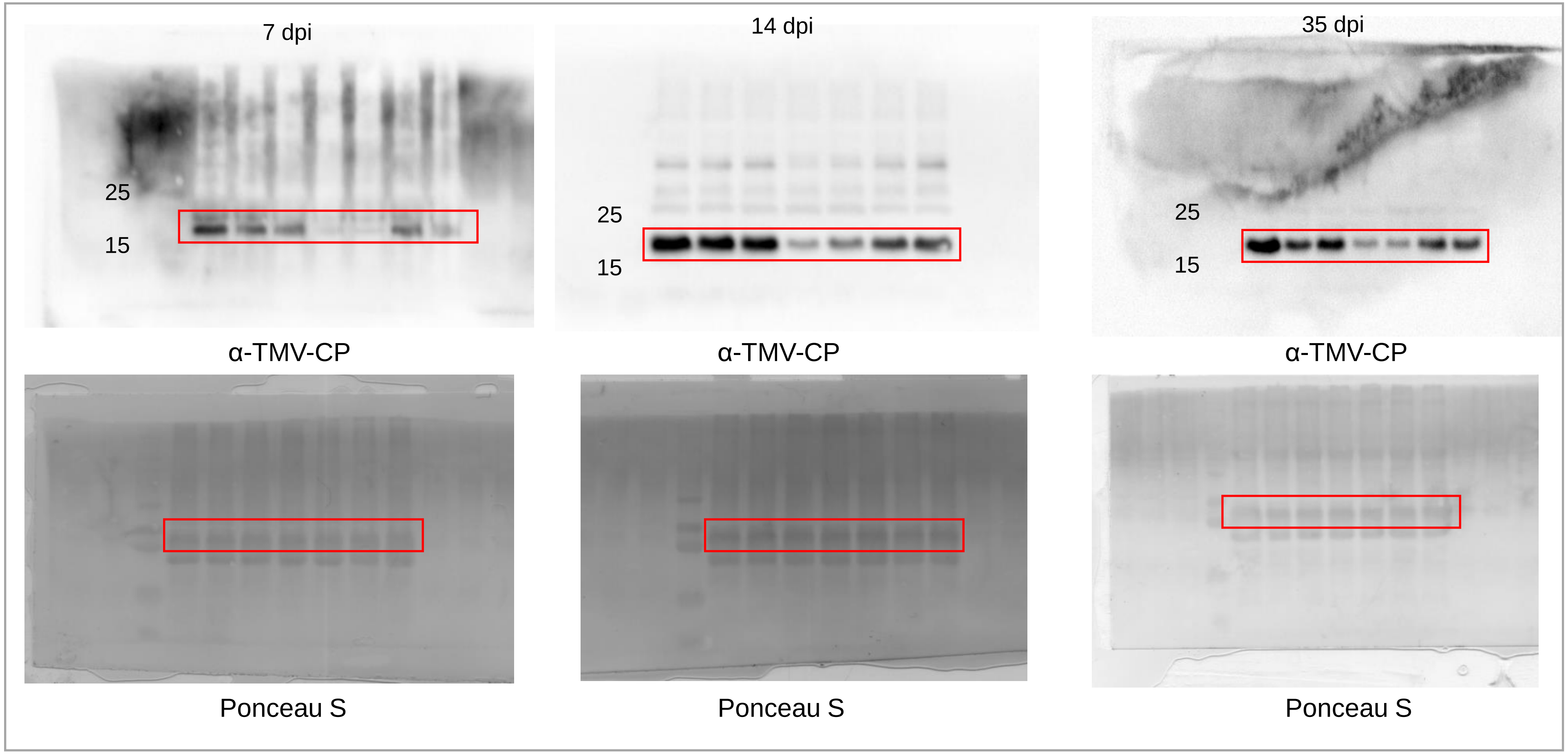
7 dpi
14 dpi
35 dpi
25
15
25
15
25
15
α-TMV-CP
α-TMV-CP
α-TMV-CP
Ponceau S
Ponceau S
Ponceau S

Fig. 5B

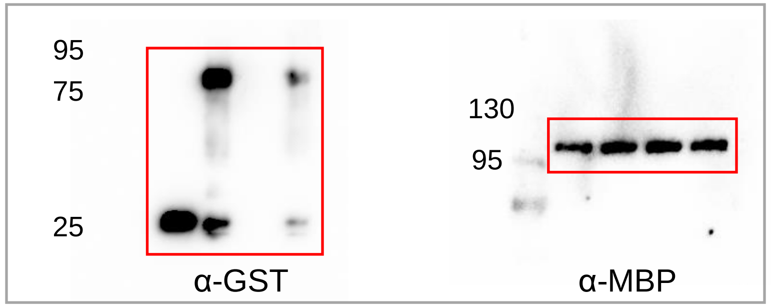

Fig. 5D

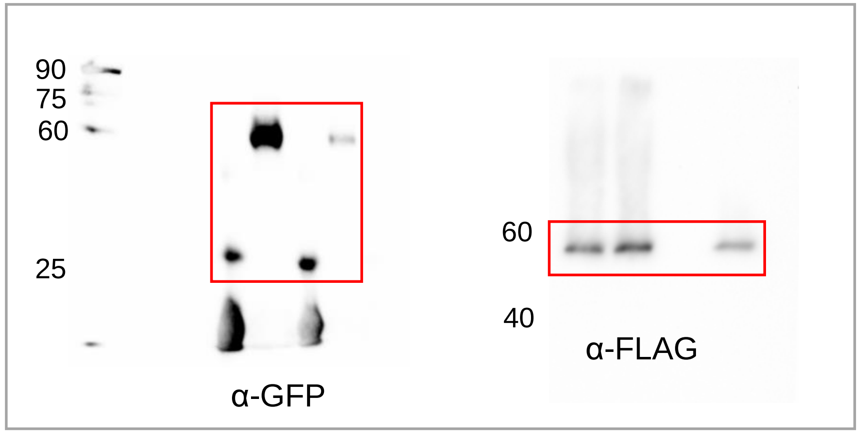

Fig. 5E

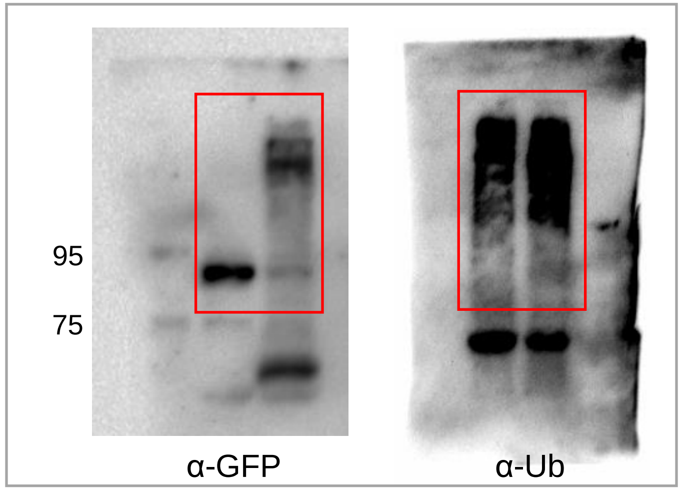

Fig. 5F

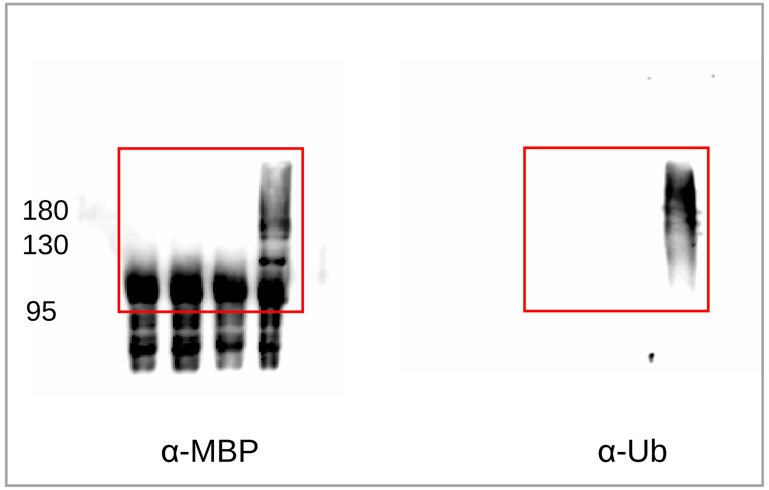

Fig. 6A

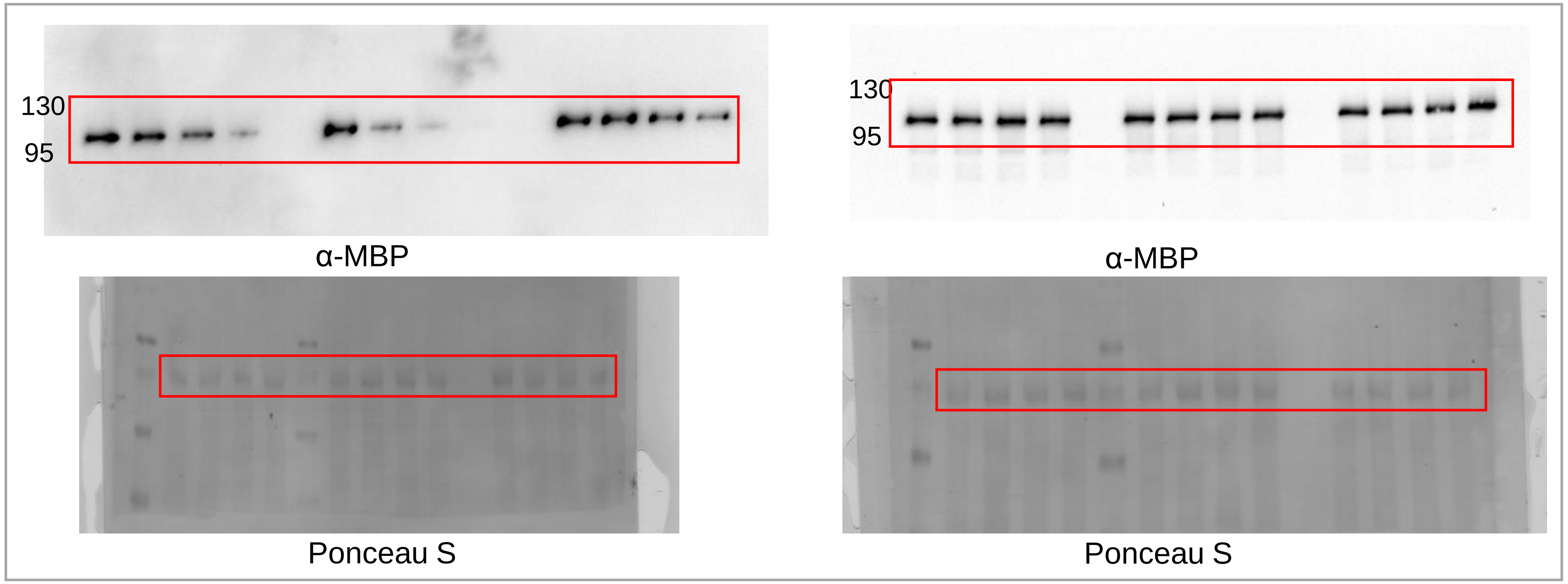
130
95
α-MBP
Ponceau S
130
95
α-MBP
Ponceau S

Fig. 6D

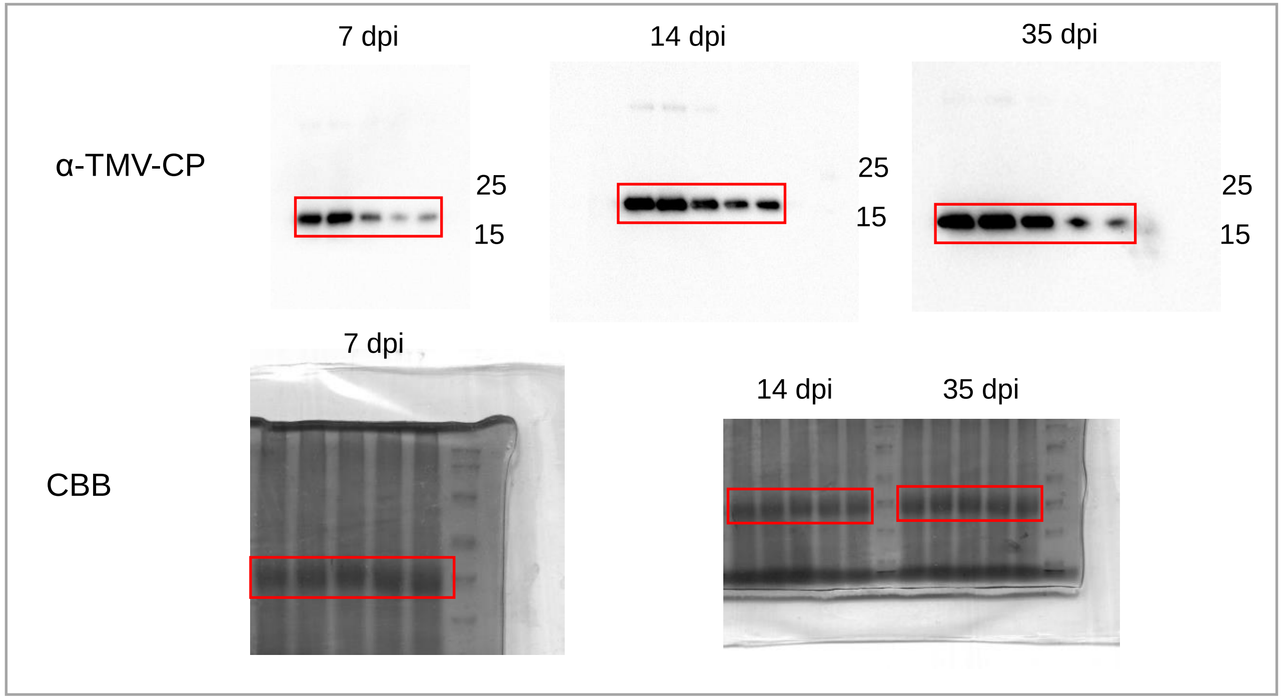
7 dpi
14 dpi
35 dpi
α-TMV-CP
25
15
25
15
25
15
7 dpi
CBB
14 dpi
35 dpi

Fig. 7B

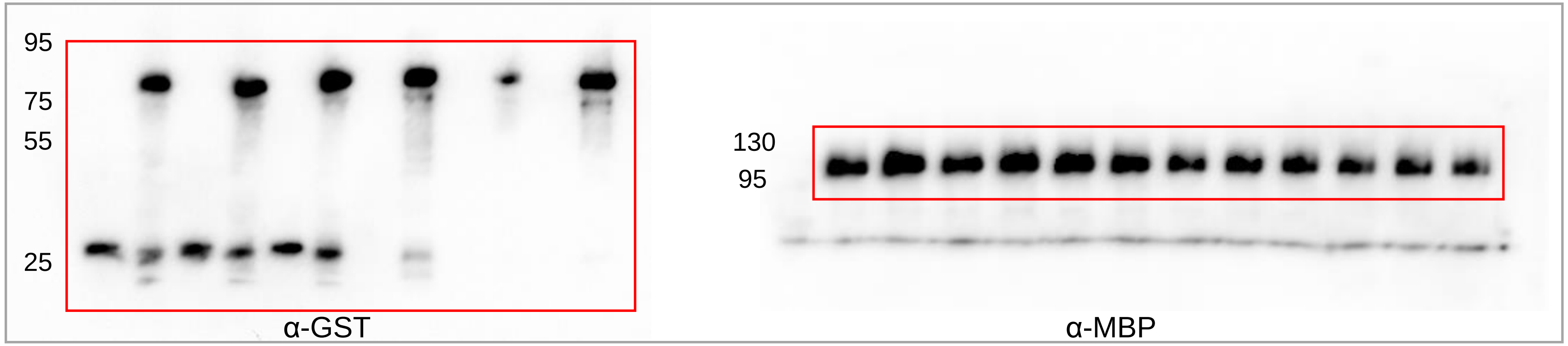

Fig. 7C

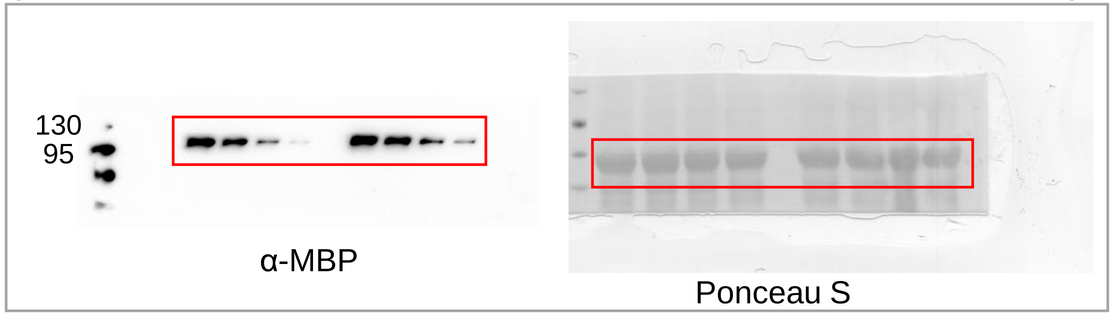

Fig. 7D

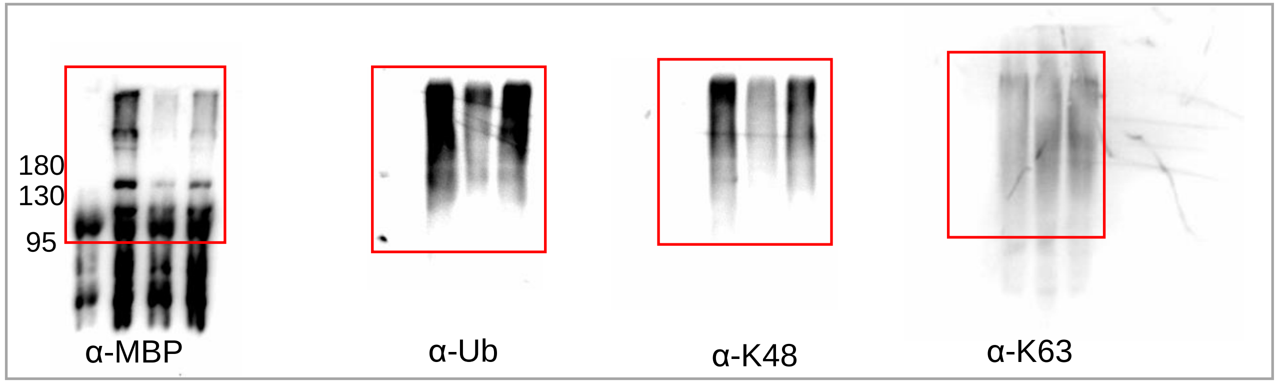

Fig. 7F

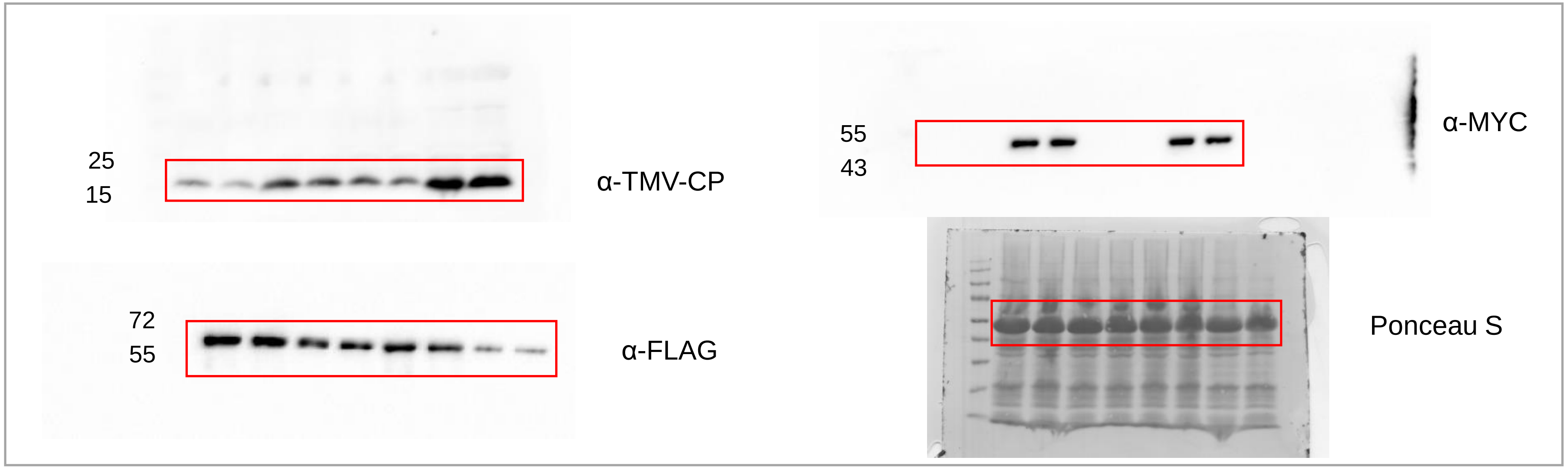

Fig. S4

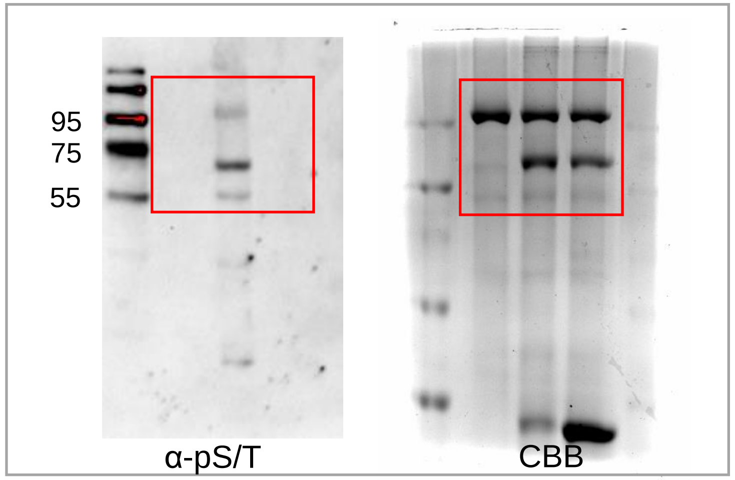

Fig. S5

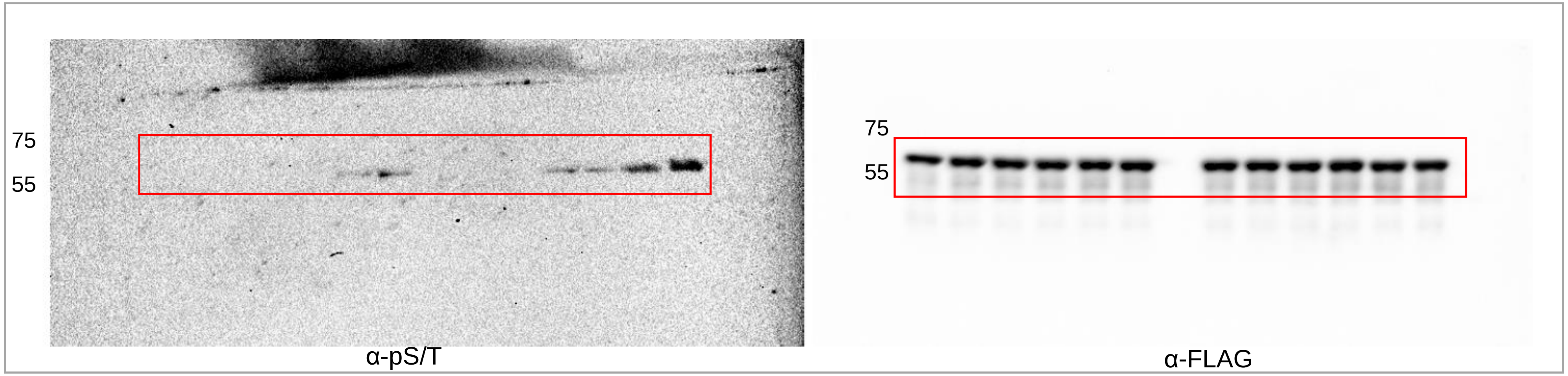

Fig. S7

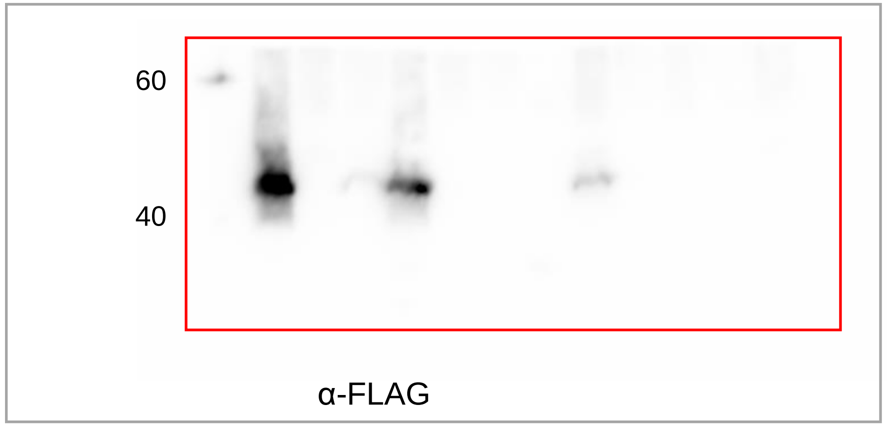

Fig. S9

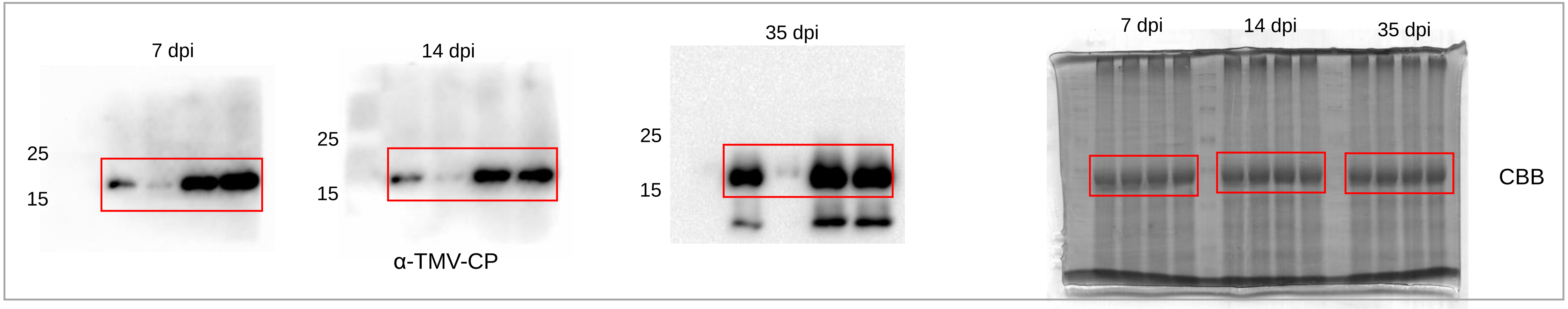

Fig. S10

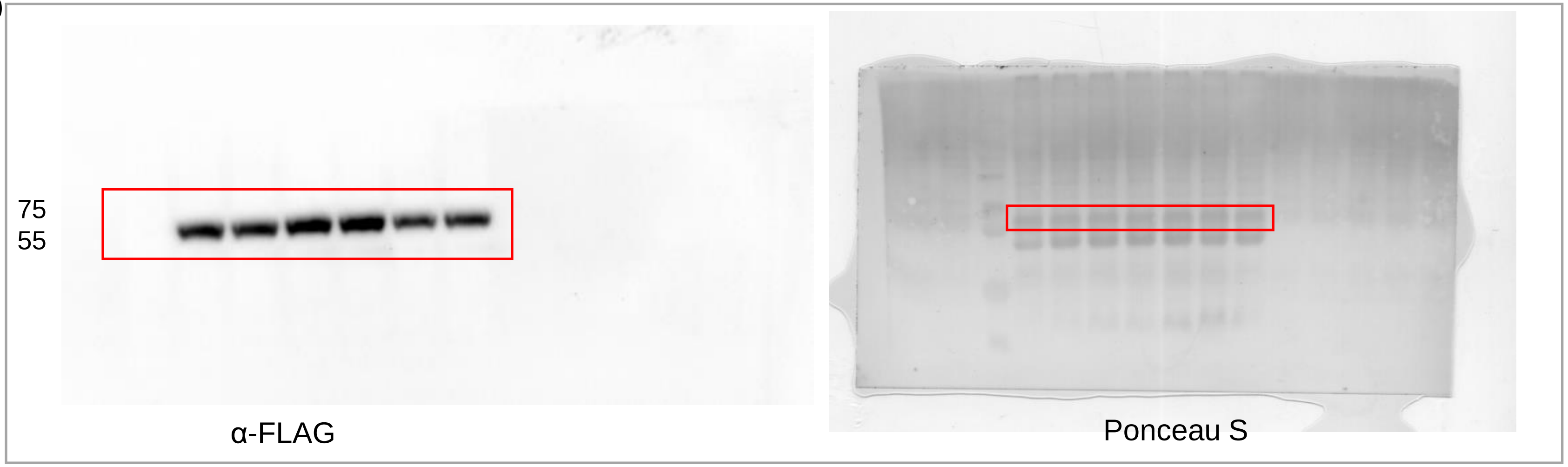

Fig. S13

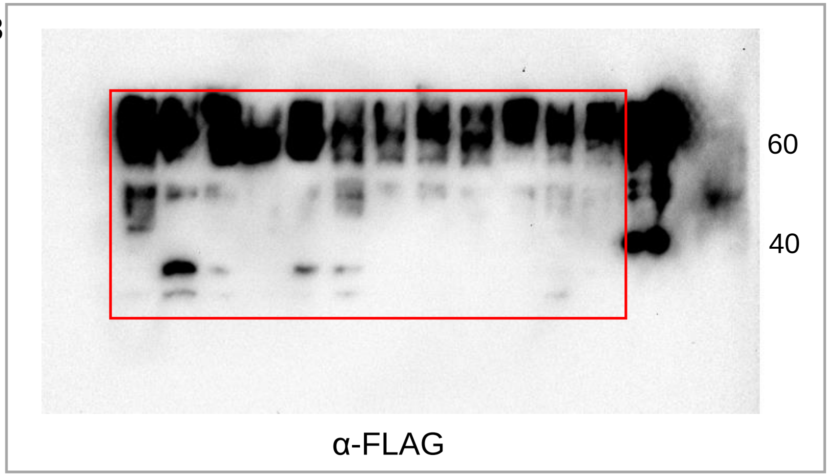

Fig. S14

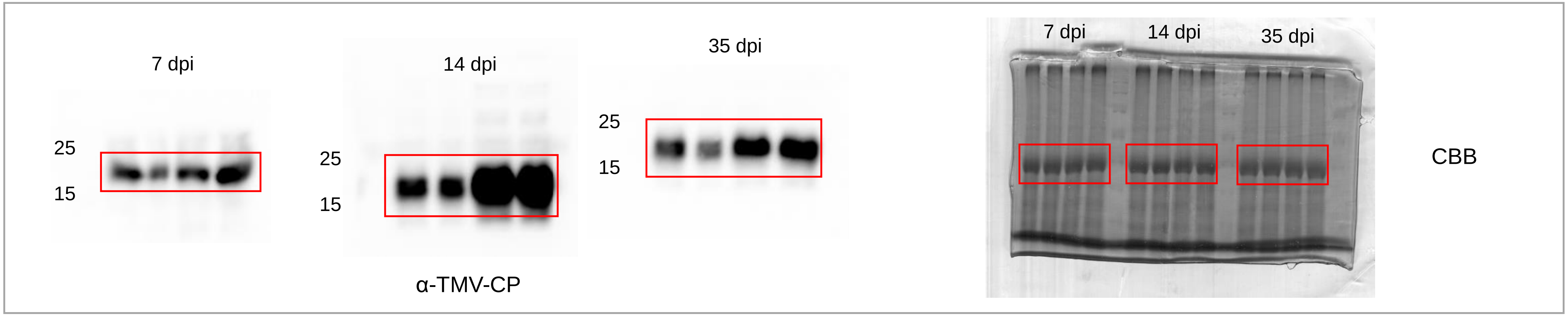

Fig. S15

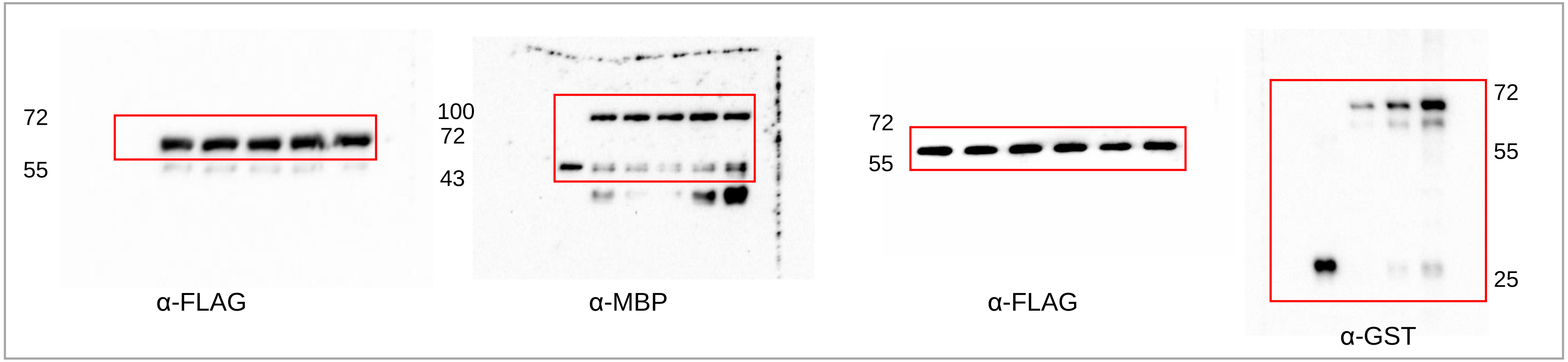

Fig. S16B

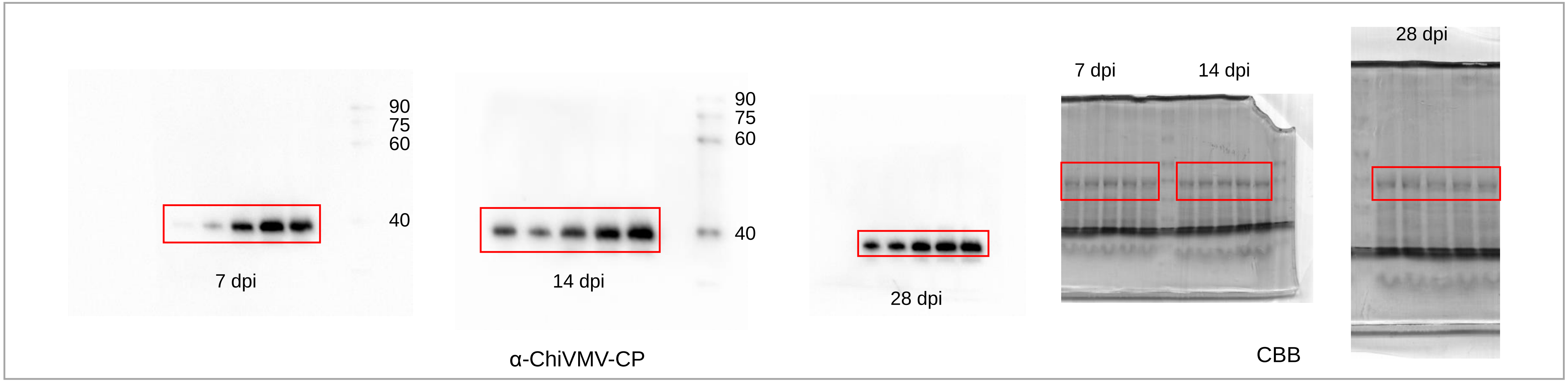

Fig. S16D

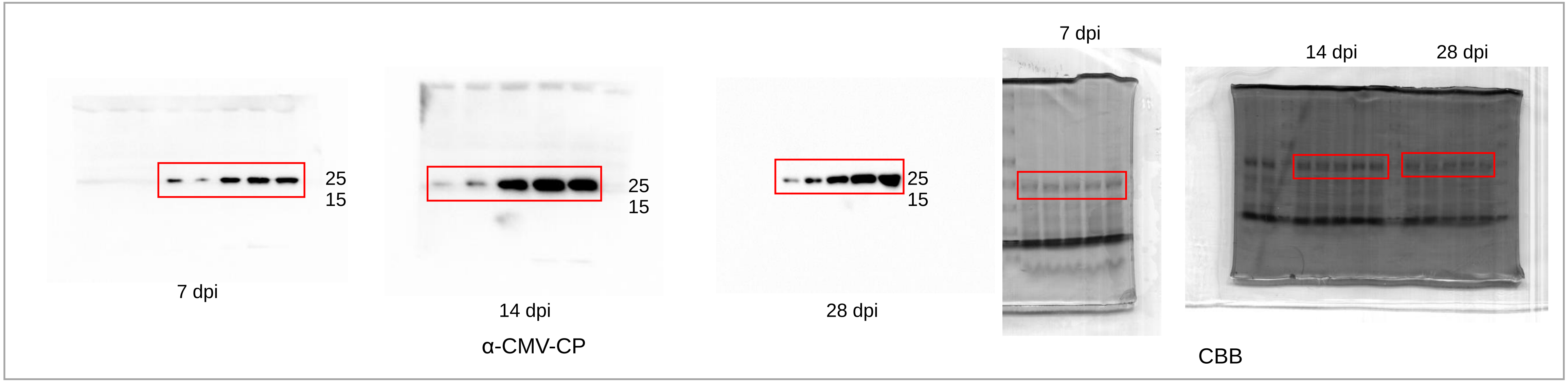

Supplement: Supplementary file 2 — Supporting Information [file ADVS-12-e06243-s001.pdf]
